# Solar Drinking Water Disinfection (SODIS) to Reduce Childhood Diarrhoea in Rural Bolivia: A Cluster-Randomized, Controlled Trial

**DOI:** 10.1371/journal.pmed.1000125

**Published:** 2009-08-18

**Authors:** Daniel Mäusezahl, Andri Christen, Gonzalo Duran Pacheco, Fidel Alvarez Tellez, Mercedes Iriarte, Maria E. Zapata, Myriam Cevallos, Jan Hattendorf, Monica Daigl Cattaneo, Benjamin Arnold, Thomas A. Smith, John M. Colford

**Affiliations:** 1 Department of Public Health and Epidemiology, Swiss Tropical Institute (STI), University of Basel, Switzerland; 2 Project Concern International (PCI), Cochabamba, Bolivia; 3 Centro de Aguas y Saneamiento Ambiental (CASA), Facultad de Tecnología, Universidad Mayor de San Simón (UMSS), Cochabamba, Bolivia; 4 Instituto de Investigaciones Biomédicas (IIBISMED), Facultad de Medicina, Universidad Mayor de San Simon, Cochabamba, Bolivia; 5 Centers for Occupational and Environmental Health and Family and Community Health, School of Public Health, Berkeley, University of California, Berkeley, California; Aga Khan University, Pakistan

## Abstract

**Background:**

Solar drinking water disinfection (SODIS) is a low-cost, point-of-use water purification method that has been disseminated globally. Laboratory studies suggest that SODIS is highly efficacious in inactivating waterborne pathogens. Previous field studies provided limited evidence for its effectiveness in reducing diarrhoea.

**Methods and Findings:**

We conducted a cluster-randomized controlled trial in 22 rural communities in Bolivia to evaluate the effect of SODIS in reducing diarrhoea among children under the age of 5 y. A local nongovernmental organisation conducted a standardised interactive SODIS-promotion campaign in 11 communities targeting households, communities, and primary schools. Mothers completed a daily child health diary for 1 y. Within the intervention arm 225 households (376 children) were trained to expose water-filled polyethyleneteraphtalate bottles to sunlight. Eleven communities (200 households, 349 children) served as a control. We recorded 166,971 person-days of observation during the trial representing 79.9% and 78.9% of the total possible person-days of child observation in intervention and control arms, respectively. Mean compliance with SODIS was 32.1%. The reported incidence rate of gastrointestinal illness in children in the intervention arm was 3.6 compared to 4.3 episodes/year at risk in the control arm. The relative rate of diarrhoea adjusted for intracluster correlation was 0.81 (95% confidence interval 0.59–1.12). The median length of diarrhoea was 3 d in both groups.

**Conclusions:**

Despite an extensive SODIS promotion campaign we found only moderate compliance with the intervention and no strong evidence for a substantive reduction in diarrhoea among children. These results suggest that there is a need for better evidence of how the well-established laboratory efficacy of this home-based water treatment method translates into field effectiveness under various cultural settings and intervention intensities. Further global promotion of SODIS for general use should be undertaken with care until such evidence is available.

**Trial Registration:**

www.ClinicalTrials.gov NCT00731497

*Please see later in the article for Editors' Summary*

## Abstract 2

Daniel Maeusezahl and colleagues conducted a cluster-randomized controlled trial in rural Bolivia of solar drinking water disinfection, and find only moderate compliance with the intervention and no evidence of reduction in diarrhea among children.

## Editors' Summary

**Background:**

Thirsty? Well, turn on the tap and have a drink of refreshing, clean, safe water. Unfortunately, more than one billion people around the world don't have this option. Instead of the endless supply of safe drinking water that people living in affluent, developed countries take for granted, more than a third of people living in developing countries only have contaminated water from rivers, lakes, or wells to drink. Because of limited access to safe drinking water, poor sanitation, and poor personal hygiene, 1.8 million people (mainly children under 5 years old) die every year from diarrheal diseases. This death toll could be greatly reduced by lowering the numbers of disease-causing microbes in household drinking water. One promising simple, low-cost, point-of-use water purification method is solar drinking water disinfection (SODIS). In SODIS, recycled transparent plastic drinks bottles containing contaminated water are exposed to full sunlight for 6 hours. During this exposure, ultraviolet radiation from the sun, together with an increase in temperature, inactivates the disease-causing organisms in the water.

**Why Was This Study Done?:**

SODIS has been promoted as an effective method to purify household water since 1999, and about 2 million people now use the approach (www.SODIS.ch). However, although SODIS works well under laboratory conditions, very few studies have investigated its ability to reduce the number of cases of diarrhea occurring in a population over a specific time period (the incidence of diarrhea) in the real world. Before any more resources are used to promote SODIS—its effective implementation requires intensive and on-going education—it is important to be sure that SODIS really does reduce the burden of diarrhea in communities in the developing world. In this study, therefore, the researchers undertake a cluster-randomized controlled trial (a study in which groups of people are randomly assigned to receive an intervention or to act as controls) in 22 rural communities in Bolivia to evaluate the ability of SODIS to reduce diarrhea in children under 5 years old.

**What Did the Researchers Do and Find?:**

For their trial, the researchers enrolled 22 rural Bolivian communities that included at least 30 children under 5 years old and that relied on drinking water resources that were contaminated with disease-causing organisms. They randomly assigned 11 communities (225 households, 376 children) to receive the intervention—a standardized, interactive SODIS promotion campaign conducted by Project Concern International (a nongovernmental organization)—and 11 communities (200 households, 349 children) to act as controls. Households in the intervention arm were trained to expose water-filled plastic bottles for at least 6 hours to sunlight using demonstrations, role play, and videos. Mothers in both arms of the trial completed a daily child health diary for a year. Almost 80% of the households self-reported using SODIS at the beginning and end of the study. However, community-based field workers estimated that only 32.1% of households on average used SODIS. Data collected in the child health diaries, which were completed on more than three-quarters of days in both arms of the trial, indicated that the children in the intervention arm had 3.6 episodes of diarrhea per year whereas the children in the control arm had 4.3 episodes of diarrhea per year. The difference in episode numbers was not statistically significant, however. That is, the small difference in the incidence of diarrhea between the arms of the trial may have occurred by chance and may not be related to the intervention.

**What Do These Findings Mean?:**

These findings indicate that, despite an intensive campaign to promote SODIS, less than a third of households in the trial routinely treated their water in the recommended manner. Moreover, these findings fail to provide strong evidence of a marked reduction of the incidence of diarrhea among children following implementation of SODIS although some aspects of the study design may have resulted in the efficacy of SODIS being underestimated. Thus, until additional studies of the effectiveness of SODIS in various real world settings have been completed, it may be unwise to extend the global promotion of SODIS for general use any further.

**Additional Information:**

Please access these Web sites via the online version of this summary at http://dx.doi.org/10.1371/journal.pmed.1000125

- The *PLoS Medicine* editors wrote an editorial arguing that water should be a human right
- The World Health Organization provides information about household water treatment and safe storage http://www.who.int/household_water and about the importance of water, sanitation, and hygiene for health http://www.who.int/water_sanitation_health/en/index.html (in several languages)
- The SODIS Reference Center provides detailed information about solar water disinfection (in several languages)
- The SODIS Foundation in Bolivia provides practical information for the roll-out of solar water disinfection in Latin America (in Spanish and English)
- Project Concern International provides information about its campaign to promote SODIS in Bolivia (in Spanish)
- The Water Supply and Sanitation Collaborative Council (WSSCC) is a global multi-stakeholder partnership organization with a goal of advocating to achieve sustainable water supply and sanitation for all people

## Introduction

Globally, 1.8 million people die every year from diarrhoeal diseases the vast majority of whom are children under the age of 5 y living in developing countries [1]. Unsafe water, sanitation, and hygiene are considered to be the most important global risk factors for diarrhoeal illnesses [2].

Recent systematic reviews concluded that interventions to improve the microbial quality of drinking water in households are effective at reducing diarrhoea, which is a principal source of morbidity and mortality among young children in developing countries [3]–[5]. One widely promoted water disinfection method with encouraging evidence of efficacy in laboratory settings is solar drinking water disinfection (SODIS) [6]. Global efforts are underway to promote SODIS as a simple, environmentally sustainable, low-cost solution for household drinking water treatment and safe storage (www.who.int/household_water, www.sodisafricanet.org). SODIS is currently promoted in more than 30 countries worldwide (www.sodis.ch) and in at least seven Latin American countries through the SODIS Foundation including in Bolivia.

Despite this widespread promotion, evidence of the effectiveness of SODIS from field studies is limited. The three reported SODIS trials to date implemented the intervention at the household level, two of them in highly controlled settings that ensured very high compliance [7]–[9]. The highest reduction in incidence (36%) was recorded in a trial carried out among 200 children in an urban slum in Vellore, India [9].

Because SODIS is a behavioural intervention designed to reduce infectious diarrhoea, disease transmission and its interruption likely have community level dynamics [10]. In addition, because SODIS is typically rolled out in practice through community rather than household level promotion, there is an urgent need for effectiveness data from such settings. We conducted a community-randomized intervention trial to evaluate the effectiveness of SODIS in decreasing diarrhoea in children <5 y in rural communities in Bolivia.

## Methods

### Ethics Statement

**Figure 1:**
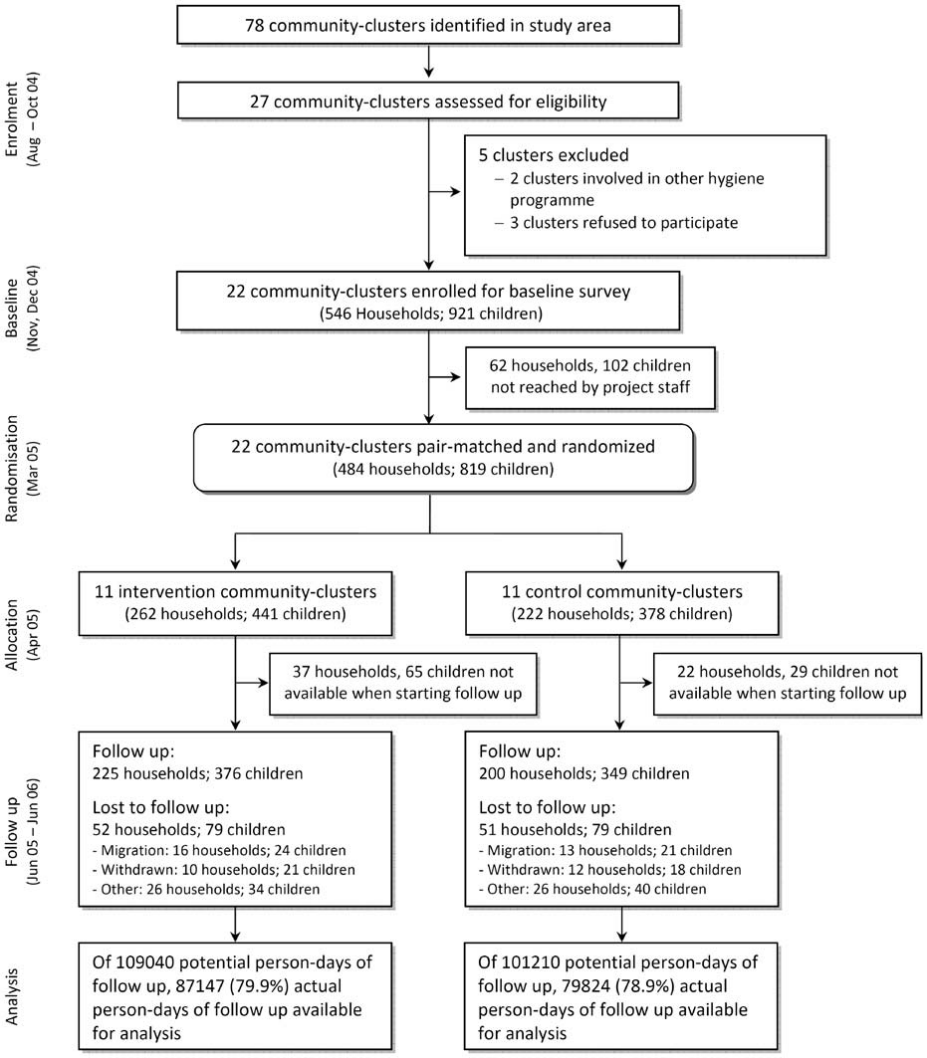
Community-randomized trial flow diagram on point-of-use SODIS in Totora District, Bolivia.

The study was approved by the three human subjects review boards of the University of Basel, Switzerland, the University of California, Berkeley, and the University of San Simon, Cochabamba, Bolivia. The Cochabamba and Totora municipal authorities also approved the study and informed consent was obtained from community leaders and male and female household heads prior to implementation of the study. Informed consent was obtained before randomisation to the treatment arms (Figure 1). Mildly ill children from households participating in the study were provided with and instructed to use oral rehydration salts, or they were referred by field staff to the local health system where clinical services were provided free of charge. The project provided transport and treatment costs for those patients. All project staff completed training on research ethics (www.fhi.org/training/sp/Retc/). Project staff comprised all project personnel of all project partners. Field staff comprised all personnel working in our laboratories and at our Totora field station including data enumerators and data- and project-management staff, supervisors, and community-based field workers living in the study communities. The trial protocol (Text S1) and the CONSORT statement checklist (Text S2) are available online as supporting information.

### Site and Population

Our trial, the Bolivia Water Evaluation Trial (BoliviaWET), was conducted in an ethnically homogeneous Quechua setting in rural Totora District, Cochabamba Department, Bolivia. Our study was part of a comprehensive SODIS roll-out programme in collaboration with Project Concern International, a nongovernmental organisation (NGO). Most of the local residents are farmers, typically living in small compounds of three buildings with mud floors, with five or more persons sleeping in the same room. Our own surveys showed that 15% of homes have a latrine or other sanitary facilities and that most residents defecate in the nearby environment.

Drinking water is typically stored in 10-l plastic buckets or open jerry cans of 5–20 l in the household. Baseline assessments of the drinking water quality in the home indicated a median contamination of thermotolerant coliforms (TTC) of 32 TTC/100 ml (interquartile range (IQR) = 3–344; *n* = 223). Samples of at least one water source per community were tested for *Giardia lamblia* and *Cryptosporidium parvum*. The two parasites were detected in 18/24 and 11/23 water samples, respectively.

Parasites were detected by using immunomagnetic separation and PCR techniques [11]. Piped water, when available, is not chlorinated.

### Design

Twenty-seven of 78 communities in the study area fulfilled the selection criteria (geographically accessible all year round; at least 30 children <5 y; reliance on contaminated drinking water sources). Two communities were excluded because of other ongoing health and hygiene campaigns, and three communities withdrew participation before baseline activities because of a change in political leadership. Community health workers undertook a census and identified households with at least one child <5 y. All children <5 y were enrolled in the participating villages.

We pair-matched communities on the incidence of child diarrhoea as measured in an 8-wk baseline survey [12]. The intervention was then assigned randomly to one community within each of the 11 consecutive pairs. This assignment was done during a public event because key political stakeholders were worried about possible backlash, public outcry, or a drop-off in group participation, which would result from providing some members with a new benefit while others got “nothing.” It was agreed that a public drawing event was necessary to increase perceived fairness among the participating district and municipal authorities. Three authorities, the district head (Alcalde), representatives of the Ministries of Health and Education, and the deputy of the farmers union (Central Campesina), each drew one of two balls (with community codes inscribed that were randomly assigned beforehand) representing paired communities from a concealed box. It was agreed that the first draw assigned the community to the intervention arm. The group allocation was immediately recorded in a protocol by an independent witness. Subsequently, the witness disclosed the sequence, informed the community members and the authorities present in the town hall, and all drawers signed the protocol.

We explicitly chose community-level randomization because important components of the intervention (i.e., community efforts to encourage adoption of the SODIS-method) would occur at the community level. Randomization below the community level would not reflect the reality of scale-up programme implementation, and we would not have captured the potential community-level reinforcement of the behaviour change. Furthermore, community-level randomization is considered ethically optimal, because participants expect to equally benefit from interventions within their community [13]–[15]. Additionally, we believed cross-contamination (of the intervention) between the intervention and control communities was minimised by vast geographical dispersion of the communities. Control communities knew from the beginning of the study that they would receive the intervention as part of the NGO's development plans after study completion. It was not possible for the NGO to carry out the intervention in all the communities at the same time, thus making randomization feasible and acceptable to the three ethical review boards overseeing the study.

Sample size was calculated according to methods outlined by Hayes and Bennett [16], assuming an incidence rate (IR) in the control villages of five episodes/child/year [17], and accounting for clustering, the number of episodes, and the expected effect. We assumed a coefficient of between-cluster variation (*k*) of similar studies, between 0.1–0.25 (as cited by Hayes and Bennett) and a minimum of 10 child-years of observation per cluster [16]. We calculated that nine pairs of clusters were required to detect a difference of at least 33% in the IR between the control and intervention arms with 80% power, *k* = 0.20 and an alpha level of 0.05. Anticipating a drop-out of at least one cluster per arm and a loss of follow-up of individuals, the final sample size was adjusted to 11 pairs with 30 children per community cluster. We powered the study to detect a 33% reduction in diarrhoea incidence after reviewing the evidence base for point-of-use water treatment at the time of the study's inception in 2002 [18].

### Implementation of the Intervention

The SODIS intervention was designed according to the published guidelines for national SODIS dissemination (http://www.sodis.ch/files/TrainingManual_sm.pdf). Promotion activities were targeted at primary caregivers and all household members (biweekly), whole communities (monthly), and primary schools (three times) by the NGO as part of its regional community development programme. Eleven communities (262 households and 441 children) were randomized to the intervention; 11 communities (222 households, 378 children) served as a control group (Figure 1). The implementation scheme and detailed description of the intervention in the intervention arm (and the control arms after study end) are described in Figure S1. For a period of 15 mo an intensive, standardised, and repeated interactive promotion of the SODIS method was implemented in the intervention communities beginning 3 mo before the start of follow-up.

Within the intervention arm, participating households were supplied regularly with clean, recycled polyethyleneteraphtalate (PET) bottles. The households were taught through demonstrations, role plays, video, and other approaches to expose the water-filled bottles for at least 6 h to the sun. NGO staff emphasized the importance and benefits of drinking only treated water (especially for children), explained the germ–disease concept, and promoted hygiene measures such as safe drinking water storage and hand washing as they relate to the understanding of drinking water and the faecal–oral route of transmission of pathogens (Figure S1). During household visits the NGO staff encouraged all household members to apply the method, answered questions, and assisted mothers and primary caregivers to integrate the water treatment into daily life. The same intervention (in terms of contents and messages) was supplied to the communities in the control arm by the NGO-staff at the end of the study (Figure S1).

### Outcome

The primary outcome was the IR of diarrhoea among children <5 y, defined as number of diarrhoea episodes per child per year obtained from daily assessment of individual diarrhoea occurrence. We applied the WHO definition for diarrhoea of three or more watery bowel movements or at least one mucoid/bloody stool within 24 h [19],[20]. We defined a new episode of diarrhoea as the occurrence of diarrhoea after a period of 3 d symptom-free [20]–[22]. An episode of diarrhoea was labelled “dysentery” if signs of blood or mucus in the stool were recorded at any time. We also calculated the longitudinal prevalence (number of days a child suffered diarrhoea divided by the number of days of observation) because of its closer relation to severity, growth faltering, and mortality than diarrhoea incidence [19],[23]. Severe diarrhoea was defined as the occurrence of diarrhoea on more than 10% of the observed days [24].

### Data Collection and Field Staff

The primary outcome was measured by community-based field workers who were recruited nearby and who lived one per community during data collection periods. The field workers were extensively trained in interviewing and epidemiological observation techniques, data checking, recording, and in general approaches to community motivation. Community-based field workers were randomly rotated between communities every 3 mo. Child morbidity was reported by the closest caregiver using the vernacular term “K'echalera,” which had been established previously to correspond to the WHO definition of diarrhoea [25]. Mothers or closest caretakers kept a 7-d morbidity diary recording daily any occurrence of diarrhoea, fever, cough, and eye irritations in study participants [25]. Community-based field workers visited households weekly to collect the health diaries, and supervisors revisited an average 7% of homes. Discrepancies between supervisors and community-based field workers' records were clarified during a joint home revisit. Child exposure risks were also assessed by community-based staff interviewing mothers once during baseline and twice during the 1-y follow-up.

Compliance with the SODIS method was measured using four different subjective and objective indicators. Three of the indicators were assessed by field staff independent from the implementing NGO: (i) the number of SODIS-bottles exposed to sunlight and, (ii) the number of bottles ready-to-drink in the living space, and (iii) the personal judgment about families' user-status was provided by community-based field workers living among the families in the intervention arm. Judgement criteria for this main compliance indicator study included observing regular SODIS practice and bottles exposed to sun or ready to drink in the kitchen and being offered SODIS-treated water upon request. The fourth SODIS-use indicator was based on self-reporting and caregivers' knowledge of and attitudes toward the intervention that was assessed at the beginning (i.e., 3 mo after start of the intervention) and at the end of the 12-mo follow-up period.

### Statistical Analysis

An intention-to-treat analysis was applied comparing the IR of diarrhoea between children <5 y in intervention and control communities. Diarrhoea prevalence (PR) and severe diarrhoea (SD) were additionally analysed. Generalized linear mixed models (GLMM) were fitted to allow for the hierarchical structure of the study design (pair-matched clusters). In contrast to our original trial protocol we selected the GLMM approach rather than generalized estimating equations (GEE) because recent publications indicated that the latter method requires a larger number of clusters to produce consistent estimates [26].

The crude (unadjusted) model included only the design factors and the intervention effect [12],[27]. Further models included potential confounders (selected a priori: child's age, sex, child hand-washing behaviour, and water treatment at baseline). Following an evaluation of the best fit, the GLMM included the log link function for negative binomial data (IR) and logit for binomial data (PR and SD). Denoting the link function of the outcome *Y* by *g*(*E*(*Y*)), the crude and adjusted models were: *g*(*E*(*Y*_ijk_)) = μ+*B*_i_+τ_j_+ξ_ij_, and *g*(*E*(*Y*_ijk_)) = μ+*B*_i_+τ_j_+ξ_ij_+*x'b* where *Y*_ijk_ denotes the observed outcome value for the *k*th individual from a community allocated to the *j*th intervention, in the *i*th pair, μ is the general mean, *B*_i_ is the random effect of the *i*th pair ≈*N*(0, σ^2^_p_), τ_j_ is the fixed effect of the SODIS intervention, and ξ_ij_ is the random effect of the interaction of the *i*th pair with the *j*th intervention applied to the community ≈*N*(0, σ^2^_pt_) (signifying the within-pair cluster variance and used as error term for τ_j_), *x* is the vector of potential confounding factors, and *b* the vector of the corresponding regression coefficients.

The intracluster correlation coefficient (ICC) and the coefficient of between-cluster variation (*k*) were calculated after data collection to validate the degree of clustering and our assumptions for the sample size. ICC and *k* were estimated from the unscaled variance of the IR's GLMM. To estimate the uncertainty of ICC and *k*, we obtained the 95% credible region (Bayesian equivalent of 95% confidence interval [CI]) through an analogous Bayesian hierarchical regression [28]. Noninformative priors were used. The statistical analyses were performed using SAS software v9.1 (PROC GLIMMIX, SAS Institute Inc.) and WinBUGS v1.4 (Imperial College and MRC).

## Results

### Participant Flow and Recruitment

Among the 1,187 households in the 22 communities there were 546 that met the inclusion criteria (Figure 1). The median number of participating households with children <5 y per community was 22. Because of political unrest and national election campaigns in 2005 a period of 6 mo passed between the baseline and the start of follow-up. Subsequently, 62 households (102 children) were no longer traceable before randomisation, and 59 households (37 intervention, 22 control) were lost before data collection had started. The loss to follow-up was balanced in intervention and control arms. Data were obtained from 376 children (225 households) in the intervention and 349 children (200 households) in the control arm, thus reaching our originally planned sample size.

Follow-up started in June 2005 and ended in June 2006. During the 51 wk of the study, information on the occurrence of diarrhoea was collected for 166,971 person-days representing 79.9% and 78.9% of the total possible person-days of child observation in intervention and control arms. We excluded from the potential observation time the experience of 94 children who dropped out before the start of follow-up. National festivities, holidays, and political unrest over the entire year amounted to further 9 wk during which outcome surveillance needed to be suspended. The main reasons for incomplete data collection were migration (28%) and withdrawal (67%). Supervisors reevaluated the outcome during 984 unannounced random home visits, and discrepancies between community-based field workers' and supervisors' records were found for five (0.5%) of all visits.

### Baseline Characteristics

At baseline the households in the different study arms were well balanced on multiple other factors suggesting successful randomisation (Table 1). The main types of water sources for household chores and drinking were similar in both arms as was the distance to the source (median distance 50 m and 30 m in the control and intervention arms, respectively). Storing water for longer than 2 d was more common among the intervention (26.8%) than the control arm (13.9%). Nearly 30% of all households reported treating water regularly before drinking. Boiling was the most common water treatment before the trial (20.2% in both arms).

**Table 1:** Baseline community and household characteristics of a community-randomized trial of SODIS.

Category	Description	*n* Children or Households	Control 11 Clusters	*n* Children or Households	Intervention 11 Clusters
**Demography**	Community size: *n* of households [mean (SD)]	—	50 (20)	—	58 (20)
	Household size: *n* of household members [mean (SD)]	N = 222	6.2 (2.1)	N = 262	6.3 (2.6)
	*n* of children <5 y per household [mean (SD)]	—	1.8 (0.7)	—	1.7 (0.8)
	*n* of children <5 y per community [mean (SD)]	—	35.3 (6.6)	—	41.4 (9.9)
	Female household head [*n* (%)]	—	20 (9.0)	—	14 (5.4)
	Closest child caregiver (female)	—	223 (99.5)	—	266 (99.6)
	Age of closest child caregiver (y) [mean (SD)]	—	31(9)	—	30 (10)
	*n* of children <1 y	—	65 (4.7)	—	67 (4.1)
	*n* of children <5 y	—	369 (26.6)	—	426 (25.9)
**Education**	Household chief: reported years of education [mean (SD)]	N = 167	4.1 (2.6)	N = 178	4.2 (2.4)
	Closest child caregiver: reported years of education [mean SD)]	N = 179	2.5 (1.9)	N = 198	2.7 (1.8)
**Socio-economic **	Main occupation of the household chief as farmer	N = 208	180 (86.5)	N = 228	207 (90.8)
**variables**	Ownership of truck, car, or motorbike	—	12 (5.8)		14 (6.2)
	Ownership of radio	—	129 (86.1)		194 (85.1)
	Ownership of bicycle	—	109 (52.4)		121 (53.1)
	Ownership of television	—	24 (11.5)		15 (6.6)
	*n* of rooms in the house [mean (SD)]	—	2.9 (1.4)		2.8 (1.2)
**Water management **	Spring as source of drinking water	N = 208	100 (48.1)	N = 228	136 (59.6)
**and consumption**	Tap as source of drinking water	—	108 (51.9)	—	129 (56.6)
	River as source of drinking water	—	46 (22.1)	—	29 (12.7)
	Rain as source of drinking water	—	31 (14.9)	—	71 (31.1)
	Dug well as source of drinking water	—	31 (14.9)	—	37 (16.2)
	Distance to water source (m) [median (Q1, Q3)]	—	50 (7.5, 100)	—	30 (6, 150)
	Container for water collection: plastic bucket	—	189 (90.9)	—	205 (89.9)
	Container for water collection: jerry can	—	165 (79.3)	—	156 (68.4)
	Container for water collection: bottles	—	32 (15.4)	—	36 (15.8)
	Container for water collection: jar/pitcher	—	13 (6.3)	—	20 (8.8)
	Container for water collection: barrel	—	10 (4.8)	—	25 (10.9)
	Child's consumption of untreated water (glasses/day) [mean (SD)]	M = 318	1.2 (1.2)	M = 359	1.2 (1.4)
	Treat water before drinking	N = 208	59 (28.4)	N = 228	67 (29.4)
	Store water for >2 d	—	29 (13.9)	—	61 (26.8)
	Water storage container: jerry can	—	23 (11.1)	—	49 (21.5)
	Water storage container: plastic bucket	—	17 (8.2)	—	37 (16.2)
	Water turbidity in water storage container >30 NTU	—	13 (11.2)	—	24 (18.8)
**Sanitation**	Reported *n* of interviewee's hand washing per day [mean (SD)]	N = 177	3.8 (1.7)	N = 200	4.1 (1.8)
	Reported *n* of child hand washing per day [mean (SD)]	M = 348	2.5 (1.2)	M = 376	2.6 (1.4)
	Child washes hands: before eating	—	228 (65.5)	—	270 (71.8)
	Child washes hands: when hands are dirty	—	62 (17.8)	—	56 (14.9)
	Child washes hands: other occasions	—	58 (16.7)	—	50 (13.3)
	Latrine present	N = 208	27 (13.0)	N = 228	38 (16.7)
	Use of latrine by the interviewee (day or night)	—	15 (7.2)	—	20 (8.8)
	Feces visible in yard	N = 202	121 (59.9)	N = 219	124 (56.6)

Data shows numbers and percentages unless otherwise specified. Baseline data from December 2004.

Abbreviations: 30NTU, threshold for efficacious pathogen-inactivation of the SODIS method; M, number of children; N, number of households; NTU, nephelometric units; SD, standard deviation.

### Intervention and Attendance

The NGO conducted 210 community events and 4,385 motivational household visits in intervention communities; 3,060 visits occurred in the households with children <5 y followed up and analysed for the study, and 1,325 household visits took place in homes that were not taking part in the study. Study households attended a median of nine community events (IQR = 5–12) and were visited by the SODIS-programme team a median 11 times at home (IQR = 7–18). To ensure a sufficient number of PET bottles, the NGO provided as many SODIS-bottles as required by participants (mean 955 bottles/community).

### Diarrhoeal Illness in the Control and Intervention Arm

Children in the SODIS-intervention arm reported a total of 808 episodes or a mean of 3.6 per child per year-at-risk (Table 2). In the control arm there were 887 episodes and an annual mean of 4.3 per child per year. In both arms median length of episodes was 3 d. The unadjusted relative rate (RR) estimate (0.81, 95% CI 0.59–1.12) suggested no statistically significant difference in the number of diarrhoea episodes between the SODIS and control arms of the study (Table 3). In an analysis of the longitudinal prevalence of diarrhoea we found no significant treatment effect (odds ratio [OR] = 0.92, 95% CI 0.66–1.29). Furthermore, no strong evidence was detected for the reduction of odds of severe diarrhoea cases (OR = 0.91, 95% CI 0.51–1.63) and dysentery (OR = 0.80, 95% CI 0.55–1.17).

**Table 2:** Diarrhoea episodes, length of illness, and days ill with diarrhoea.

Health Condition	Class or Parameter	*n*	Control	*n*	Intervention
**Diarrhoea illness overview**		**Children**		**Children**	
Days under observation	Median (Q1, Q3)	349	263 (213, 274)	376	263 (222, 273)
Days at risk	Median (Q1, Q3)	349	246 (192, 265)	376	247 (202, 265)
*n* Episodes	Median (Q1, Q3)	349	1 (0, 3)	376	1 (0, 3)
*n* Dysentery episodes	Median (Q1, Q3)	349	1 (0, 2)	376	1 (0, 2)
Days spent ill	Median (Q1, Q3)	349	4 (0, 11)	376	4 (0, 12)
Episode length (d)	Median (Q1, Q3)	349	3 (1, 5)	376	3 (2, 5)
Days under observation	Total		79,829		87,140
Days at risk	Total		75,077		82,682
*n* Episodes	Total		887		808
*n* Dysentery episodes	Total		460		431
Days spent ill	Total		3,111		3,038
**Diarrhoea incidence**	**Age class**	**Children**	**IR**	**Children**	**IR**
*n* Episodes/(child×year at risk)	<1	16	7.8	15	11.1
	1–2	67	7.1	70	5.5
	2–3	67	4.3	82	3.8
	3–4	77	3.2	75	2.8
	4–5	71	3.4	80	2.1
	5–6	50	2.7	53	2.5
	**Total** a	349	4.3	376	3.6
**Diarrhoea prevalence**	**Age class**	**Children**	**Mean (SD)**	**Children**	**Mean (SD)**
*n* Days ill/(child×year)	<1	16	27.4 (28.3)	15	42.3 (40.7)
	1–2	67	31.4 (42.2)	70	23.0 (26.1)
	2–3	67	19.0 (47.5)	82	16.4 (28.4)
	3–4	77	11.7 (24.5)	75	7.3 (9.7)
	4–5	71	9.5 (15.1)	80	6.2 (12.4)
	5–6	50	6.9 (11.8)	53	7.7 (10.4)
	**Total** a	349	16.5 (32.8)	376	13.5 (22.4)
**Diarrhoea illness**	**Days spent ill**	**Children**	**Percent**	**Children**	**Percent**
	0 d	97	27.8	126	33.5
	1–2 d	50	14.3	42	11.2
	3–7 d	91	26.1	80	21.3
	8–14 d	49	14.0	59	15.7
	15–21 d	27	7.7	33	8.8
	22–40 d	18	5.2	21	5.6
	>40 d	17	4.9	15	4.0
	**Total**	349	100	376	100
**Diarrhoea illness duration**	**Episode duration**	**Episodes**	**Percent**	**Episodes**	**Percent**
	1 day	250	28.2	191	23.6
	2–3 d	303	34.2	292	36.1
	4–7 d	258	29.1	250	30.9
	8–13 d	54	6.1	59	7.3
	>13 d	22	2.5	16	1.9
	**Total**	887	100	808	100
**Prevalence of other symptoms (d/[child×year])**		**Children**	**Mean (SD)**	**Children**	**Mean (SD)**
Vomit		349	5.5 (13.2)	376	4.0 (8.9)
Fever		349	21.0 (33.0)	376	15.1 (19.8)
Cough		349	41.9 (48.3)	376	30.9 (39.4)
Eyes irritation		349	12.8 (29.8)	376	8.3 (19.5)

a Includes one child per treatment arm with unknown age. SD, standard deviation.

**Table 3:** Effect of SODIS on diarrhoea episodes, longitudinal prevalence, severe diarrhoea, and dysentery episodes.

Outcome	Model	*n* Children	Parameter	RR/OR	95% CI	*p*-Value
***n*** ** Episodes (RR)**	Unadjusted	725	Intervention	0.81	(0.59–1.12)	0.19
	Adjusted	644	Intervention	0.74	(0.50–1.11)	0.14
			Age	0.75	(0.70–0.81)	<0.001
			Sex	1.03	(0.84–1.26)	0.80
			Water treatment	1.05	(0.81–1.36)	0.69
			Hand washing	0.93	(0.85–1.02)	0.13
**Prevalence (OR)**	Unadjusted	725	Intervention	0.92	(0.66–1.29)	0.62
	Adjusted	644	Intervention	0.91	(0.64– 1.30)	0.60
			Age	0.67	(0.61–0.73)	<0.001
			Sex	1.05	(0.84–1.31)	0.68
			Water treatment	1.00	(0.76–1.33)	0.97
			Hand washing	0.94	(0.84–1.04)	0.23
**Severe diarrhoea (OR)**	Unadjusted	643	Intervention	0.91	(0.51–1.63)	0.75
	Adjusted	589	Intervention	1.02	(0.52–2.01)	0.95
			Age	0.52	(0.40–0.67)	<0.001
			Sex	1.12	(0.63–2.01)	0.69
			Water treatment	1.59	(0.81–3.12)	0.18
			Hand washing	0.94	(0.75–1.19)	0.62
**Dysentery (OR)**	Unadjusted	725	Intervention	0.80	(0.55–1.17)	0.23
	Adjusted	644	Intervention	0.75	(0.47–1.18)	0.20
			Age	0.73	(0.67–0.80)	<0.001
			Sex	1.00	(0.80–1.26)	0.97
			Water treatment	1.15	(0.87–1.53)	0.33
			Hand washing	0.91	(0.82–1.01)	0.06

Number of episodes, *n* of episodes per days at risk; prevalence, *n* of days ill per days under observation; severe diarrhoea, diarrhoea during >10% of all days (only children with more than 100 d of observation are included); unadjusted, general linear mixed models, only design factors and treatment are included; adjusted, effects of treatment and covariates; sex: 0, female; 1, male; water treatment: water treatment at baseline, 0, no treatment; 1, treatment (chlorination or boiling or SODIS); hand washing, reported number of child's hand washing per day at baseline.

**Figure 2:**
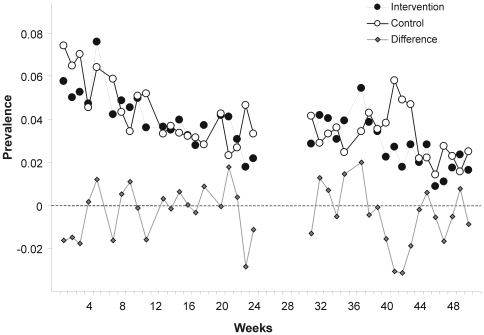
Weekly prevalence of child diarrheal illness. Weekly points are derived from daily prevalence data of each participating child.

A multivariable model adjusting for age, sex, baseline-existing water treatment practises, and child hand washing was consistent in its estimate of effect (RR = 0.74, 95% CI 0.50–1.11). We repeated the analysis by including confounding covariates in the order of occurrence of the variables in Table 3 to confirm that the conclusions were not sensitive to the choice of covariates. None of the models yielded significant results for the effect of SODIS (all *p*-values>0.1) or resulted in meaningful changes in estimates of ORs. Figure 2 shows the relationship between study time and diarrhoea in the control and intervention arm. We found no statistically significant effect of the interaction of time and intervention in a time-dependent model.

The ICC was estimated as 0.0009 with a 95% posterior credible region between (0.0001, 0.0025); *k* was estimated to be 0.27 with a 95% confidence region of (0.11, 0.46).

### Compliance

**Figure 3:**
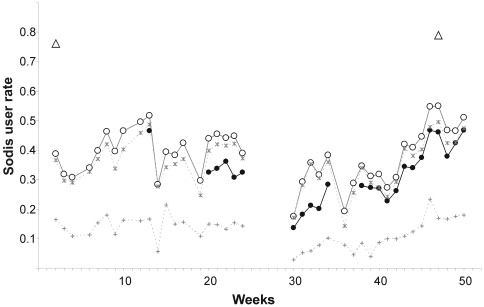
Weekly observed proportion of households using SODIS as point-of-use drinking water purification method. Open triangles, self-reported SODIS use at the beginning (after 3 mo of initial SODIS promotion) and at the end of follow-up; filled dots, SODIS use observed by project staff living in the community (see Methods for definition); open circles, SODIS bottles observed on the roof and/or in the kitchen; stars, SODIS-bottles on the roof; crosses, SODIS-bottles in the kitchen.

Community-based field workers who were living in the communities throughout the study observed a mean SODIS-user rate of 32.1% in the intervention arm (minimum 13.5%, maximum 46.8%, based on their personal judgement) (Figure 3). The mean proportion of households with SODIS-bottles exposed to the sun was 5 percentage points higher than the assessment by community-based field workers. In contrast, almost 80% of the households reported using SODIS at the beginning and end of the follow-up. About 14% of the households used the method more than two-thirds (>66%) of the weeks during observation, and 43% of the households applied SODIS in more than 33% of the observed weeks (Table 4).

**Table 4:** Climatic conditions and SODIS use of a cluster-randomized trial involving 22 rural communities of Totora District, Bolivia.

Category	Description	Control (*n* = 11 Clusters)	Intervention (*n* = 11 Clusters)
Climate	Percentage of sunny days (>6 h sunshine) [median of clusters (min, max)]	70 (57, 78)	67 (44, 77)
	Average duration of sunshine [median of clusters (min, max)]	7.0 (6.3, 8.0)	7.1 (4.5, 8.3)
**SODIS-use**	**Observed level of SODIS use** a	**Percentage of households**	**Percentage of households**
	0.66–1	0%	14%
	0.33–0.66	0.5%	29%
	0–0.33	99.5%	57%

a Proportion of weeks in which SODIS was used, as estimated by community-based project staff at the end of study. Households with <10 wk of observation are excluded.

### Diarrhoeal Illness by Compliance

**Figure 4:**
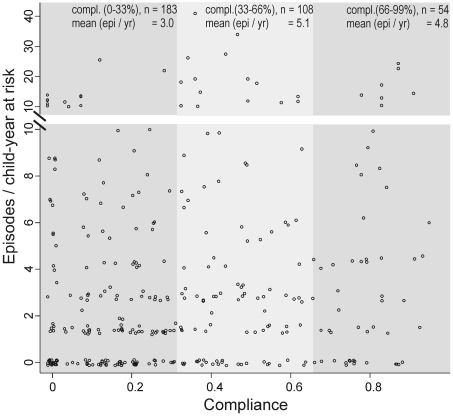
Compliance of using SODIS and child diarrhoea in rural Bolivia. Compliance of SODIS use is estimated as the proportion of weeks a family has been classified as a SODIS user by community-based project staff. Dots, number of episodes per child-year at risk. Small random noise was added to the dots to avoid over plotting. Only children with at least 110 d under observation are included.

No positive effect of compliance (proportion of weeks of observed SODIS use) on the IRs in the intervention arm was observed. The incidence did not decline with the increase of weeks using SODIS (Figure 4). Seasonal variation in compliance was observed. The proportion of SODIS-practising households was consistently below average during weeks 4–16 (January 2005–April 2006), which corresponded to the labour intensive cultivating period from November to May.

The median proportion of sunny days with more than 6 h of sunshine was 70.2% and 67.2% in intervention and control communities, respectively, consistent with the technical and climatic conditions necessary for the proper functioning of the ultraviolet SODIS purification process [29] during the study (Table 4).

## Discussion

We conducted a community-randomized trial within the operations of an ongoing national SODIS-dissemination programme, which provided an intensive training and repeated reinforcement of the SODIS intervention throughout the study period. In this context of a “natural experiment” we found a RR of 0.81 for the IR of diarrhoea episodes among children assigned to SODIS compared to controls. However, the CI was broad and included unity (RR = 0.81, 95% CI 0.59–1.12) and, therefore, we conclude that there is no strong evidence for a substantive reduction in diarrhoea among children in this setting. Subsequently, we discuss the primary outcome in the context of other study findings, and explain why we hypothesize that the true effect—if there is any—might be smaller.

First, the estimate for the longitudinal prevalence of diarrhoea was substantially smaller (OR = 0.92, 95% CI 0.66–1.29) than the estimate for incidence and there is some evidence that prevalence is a better predictor in terms of mortality and weight gain than incidence [23]. The absence of a time-intervention interaction in our time-dependent analysis suggested no increased health benefits with the ongoing intervention. Furthermore, within the intervention arm, there was no evidence that increased compliance was associated with a lower incidence of diarrhoea (Figure 4). However, we interpret this post hoc subgroup analysis cautiously because compliant SODIS users might differ in important ways from noncompliant users. A compliant SODIS user might be more accurately keeping morbidity diaries, whereas less compliant families may tend to underreport diarrhoeal illness. Or, households with a high burden of morbidity might be more likely to be compliant with the intervention. Both of these scenarios could lead to an underestimation of the effectiveness of SODIS.

Further, analysing the laboratory results from 197 randomly selected stool specimens also did not provide convincing evidence for an intervention effect: the proportion of *C. parvum* was lower in the intervention children (5/94 versus 2/103), but other pathogens were found at similar proportions in intervention and control children (*G. lamblia*, 39/94 versus 40/103; *Salmonella* sp., 2/94 versus 3/104; *Shigella* sp., 3/94 versus 3/104). In further exploring the occurrence of other illness symptoms we found the prevalence of eye irritations and cough to be lower in the intervention group compared to the control group. This difference could be the result of the hygiene component in the intervention that increased hygiene awareness among the treatment communities. An alternative explanation is that the lack of blinding led to biased (increased) health outcome reporting in the intervention group.

Due to the nature of the intervention neither participants nor personnel were blinded to treatment assignment. Ideally, blinding to the intervention allocation should apply to the NGO staff administering the SODIS intervention and our enumerators assessing outcomes [30]. Although the former could not be blinded in our study (for obvious reasons), the latter would inevitably be able to identify the intervention status of the cluster through the visible display of bottles to sunlight in the village or directly at the study home during home visits. These problems are consistent with nearly all household water treatment interventions [5] and other public health cluster randomized trials [31],[32]. Schmidt and Cairncross [33] recently argued that reporting bias may have been the dominant problem in unblinded studies included in a meta-analysis reporting a pooled estimate of a 49% reduction of diarrhoea in trials investigating the effects of drinking water quality interventions [5]. However, their review of only four available blinded trials showing no effect demonstrates weak support for contrast. In addition, all of the blinded trials exhibited analytical shortcomings or had very broad CIs suggesting very low power. In the absence of blinding—unavoidable in many behavioural change interventions or household water treatment studies—we believe that data collection independent from the implementation is a crucial factor. Future reviews should include reporting on such additional quality parameters.

In our study the lack of blinding may have reduced motivation in the control communities. However, the number of households lost during follow-up and the number of days under observation were almost identical in both arms. Additionally, the control communities knew that they would receive the intervention after study end. Finally, a reduction of diarrhoea frequency of 20% might be insufficient to be well perceived, i.e., have a noticeable impact in a population with a high burden of child diarrhoea and will, thus, not result in a sustainable behavioural change. Faecal contamination in about 60% of the yards indicates a highly contaminated environment with presumably a large potential for transmission pathways other than consuming contaminated water. This simultaneous exposure to a multiplicity of transmission pathways may explain why we found no significant diarrhoea reduction due to SODIS.

On the other hand, our result of a 19% reduction in diarrhoeal episodes appears to be roughly consistent with results of the two other SODIS trials both from Maasai cultural settings conducted by Conroy and colleagues among children <6 y and 5–16 y of age. They report a 16% reduction (in <6 y olds, 2-wk prevalence of 48.8% in intervention, and 58.1% in control group) [8] and a 10.3% reduction in the 2-wk diarrhoea prevalence (in 5–16 y olds) [7]. However, these randomized controlled trials were undertaken in a socio-cultural setting assuring a 100% compliance (as stated by the authors) in water treatment behaviour through social control by Maasai elders who promoted the method [7],[8]. In the results presented in these studies adjusted models with post hoc selected covariates were presented (i.e., no unadjusted models were provided). These trials were carried out in conditions of heavily contaminated drinking water and very high diarrhoea rates—important considerations when attempting to generalize these results. The only other—quasi-randomized—trial to estimate the effect of solar water disinfection was carried out in the urban slum in Vellore and resulted in a remarkable reduction of diarrhoea among children <5 y (IR ratio, 0.64; 95% CI 0.48–0.86) despite 86% of SODIS users also drinking untreated water [9].

To our knowledge this is the first community-randomized trial and the largest study so far to assess the effectiveness of the SODIS method under typical social and environmental conditions in a general rural population setting where children drink untreated water.

Our study was sufficiently powered to detect a 33% reduction in the effectiveness of the SODIS intervention, and we accounted for clustered design in our analysis. On the basis of a post hoc sample size calculations using the model-based estimate for the between-cluster variability (*k* = 0.27), we would have needed a study 2.5 times larger for a 20% difference to be significant.

The implementing NGO, which had global experience in disseminating SODIS, adapted a campaign to the local and cultural needs and also involved the public health and educational system in the roll-out. This comprehensive SODIS campaign resulted in a mean SODIS usage of 32% on any given study day. In using the SODIS-use indicator on the basis of the personal judgement of community-based staff, we intended to measure actual use in combining objective, visible signs of use (e.g., bottles exposed to sunlight) with proxies more responsive to actual treatment behaviour (e.g., SODIS water can be offered to drink upon request). We consider this a restrictive, more conservative definition of SODIS use compared to that in other studies, which recorded reported use [9] or the number of bottles exposed to sunlight [34]. Both are indicators that can easily and reliably be measured, but which are prone to over-reporting due to low specificity for actual use. Further studies will need to validate different compliance indicators and formally assess the dimension of reporting bias.

It is possible that respondents would like to please field staff and over-report use out of courtesy. Also, observing exposed bottles on the roof may overestimate use (Figure 3), because some households were noted anecdotally to have placed bottles on the roof to avoid discussions with the SODIS-implementing NGO staff. Figure 3 is indicative of this phenomenon, as reported use at the beginning and reported use and satisfaction with the method at the end of the study reached the 80% mark—a usage figure consistent with other studies relying on reported compliance [9] and evaluation reports from grey literature. We conclude that self-reported SODIS use may overestimate compliance and a combination of reported and objectively measurable indicators provides more accurate SODIS-compliance data.

There are limitations to our study. As in other studies [24],[35], we observed a decline in the reporting of child diarrhoea during the observational period in both arms (Figure 2). If true, seasonal variation of diarrhoea could be one possible cause; increased awareness leading to more attention to basic hygiene and hence to illness reduction may be another reason. Alternatively, the pattern could be due to survey fatigue.

Despite a comprehensive and intensive intervention promotion campaign, we detected no strong evidence for a significant reduction in the IR of diarrhoea in children <5 y in families using SODIS in our trial in a typical setting in rural Bolivia. We believe that clearer understandings of the discrepancy between laboratory and field results (obtained under typical environmental and cultural conditions), the role of compliance in effectiveness, and a direct comparison of SODIS to alternate drinking water treatment methods are needed before further global promotion of SODIS.

## Supporting Information

Alternative Language Abstract S1 Spanish translation of the abstract by MC.
(0.03 MB DOC)

Figure S1 SODIS promotion and implementation scheme (based on Perera et al. [36]).
(2.14 MB PDF)

Text S1 Trial protocol.
(0.52 MB PDF)

Text S2 CONSORT statement checklist.
(0.10 MB PDF)

## Abbreviations

CI: confidence interval
GLMM: generalized linear mixed model
ICC: intracluster correlation coefficient
IQR: interquartile range
IR: incidence rate
NGO: nongovernmental organisation
OR: odds ratio
RR: relative rate
SODIS: Solar drinking water disinfection
