# Supplementary material for: Solar Drinking Water Disinfection (SODIS) to Reduce Childhood Diarrhoea in Rural Bolivia: A Cluster-Randomized, Controlled Trial
Source: PLoS Med. 2009 Aug 18;6(8):e1000125. doi: 10.1371/journal.pmed.1000125 (PMC2719054; doi:10.1371/journal.pmed.1000125)
Supplement: Alternative Language Abstract S1 — Spanish translation of the abstract by MC. (0.03 MB DOC) [file pmed.1000125.s001.doc]

**Antecedentes**

Desinfección solar de agua (SODIS) es un método de desinfección de agua de bajo costo y a nivel de punto de consumo, que esta siendo diseminado a nivel global. Estudios de laboratorio sugieren que SODIS es altamente eficaz inactivando patógenos provenientes del agua. Previos estudios de campo han proporcionado limitada evidencia acerca de la efectividad de SODIS reduciendo diarrea.

**Métodos y resultados**

Realizamos un ensayo clínico aleatorizado por conglomerados en 22 comunidades rurales en Bolivia para evaluar el efecto de SODIS en la reducción de diarrea en niños menores de 5 años. La promoción estandartizada de SODIS así como una campaña de educación de higiene fue realizada por una ONG local en viviendas y escuelas primarias de 11 comunidades. Las madres de los niños participantes completaron un diario de salud durante un año. Dentro del grupo de intervención 225 viviendas (376 niños) fueron capacitadas para exponer al sol botellas de polyethylene terephthalate llenas de agua. Once comunidades (200 viviendas, 349 niños) sirvieron de control. Durante nuestro estudio registramos 166’971 personas-días de observación que representan 79.9% y 78.9% del total posible de personas-días de observación de niños en los respectivos grupos intervención y control. El promedio de adherencia a SODIS fue 32.1%. La incidencia de enfermedades gastrointestinales reportadas en niños en el grupo intervención fue 3.6 comparado con 4.3 episodios/año en riesgo en el grupo de control. La taza relativa de diarrea ajustada por la correlación intra- grupos (comunidades) fue 0.81 (95% CI 0.59-1-12). La mediana de la duración de diarrea fue de 3 días en ambos grupos.

**Conclusiones**

A pesar de la extensa campaña de promoción de SODIS encontramos una adherencia moderada a la intervención y no encontramos evidencia de una reducción significativa en la diarrea infantil. Estos resultados sugirieren que existe la necesidad de generar mayor evidencia de cómo la eficacia de este método casero de desinfección de agua, bien establecida en el laboratorio, puede ser traducida en efectividad en el campo bajo diferentes entornos culturales e intensidades de intervención. Además, la promoción global de SODIS para el uso general debe ser conducida con cuidado hasta que dicha evidencia sea proporcionada.
