# Supplementary material for: Solar Drinking Water Disinfection (SODIS) to Reduce Childhood Diarrhoea in Rural Bolivia: A Cluster-Randomized, Controlled Trial
Source: PLoS Med. 2009 Aug 18;6(8):e1000125. doi: 10.1371/journal.pmed.1000125 (PMC2719054; doi:10.1371/journal.pmed.1000125)
Supplement: Figure S1 — SODIS promotion and implementation scheme (based on Perera et al. [36]). (2.14 MB PDF) [file pmed.1000125.s002.pdf]

SODIS promotion and implementation scheme

Time	11 control community-clusters (222 households)		11 intervention community-clusters (262 households)		
	Community level	Household level	District level*	Community level	Household level
6 weeks baseline (Nov - March 05)	Baseline, pair-matching of control and intervention communities based on the diarrhoea incidence in children <5 years old, random assignment of the SODIS intervention to one of the community pairs§				
3 months before starting one-year follow-up (April - June 05)			A	A; B	
Follow-up: 1-6 months (June - Dec 05)		G	C	A; B; D	G; E; F
Follow-up: 7-12 months (Jan - June 06)		G	C	A; B; D	G; E; F
Intervention: 3 months (July - Sept 06)	A; B; D	E			

Symbol	Promotion activity	Content, topic number
A	Introduction to and consolidation of SODIS and related water, sanitation, hygiene, and health issues	1; 2; 3; 4; 5
B	Community event (monthly)	1; 4; 5; 6
C	School event (two monthly)	1; 8
D	Motivational micro-project	7
E	Two weekly household visits	Address day-to-day problems with SODIS-application and management. No specific hygiene messages.
F	Weekly SODIS monitoring	Observational; by community-based staff (independent from the SODIS-implementing NGO)
G	Health monitoring	Health diary kept by mothers; collected by community-based staff (independent from the SODIS-implementing NGO)

* Municipality, health - and school system

§ The standard field operations of the implementing NGO (following published guidelines) were adjusted to the needs of our trial in three aspects: i.) the NGO accommodated the outcome of the randomisation to their regional SODIS implementation plans, ii.) the frequency of household visits was increased to bi-weekly instead of monthly to accommodate likely irregular attendance at community events of remotely situated households, iii.) NGO staff was trained in documenting and record-keeping of their own field activities and attendance and main reactions of their audience.

Topic No	Content
1	SODIS and water disinfection at household level: Understanding i.) the use of SODIS in areas with microbiolologically contaminated drinking water, ii.) the synergistic effect of UV-A light and heat in pathogen-inactivation, iii.) application of the SODIS method at home. The importance of safe water consumption for family health. Alternative household water treatment methods like boiling and chlorination were mentioned and not explained in detail.
2	Sources of and value of water: Conveying the importance of water for life: for humans, animals and plants. Application of metaphors (e.g. growing of plants and raising children). Sources for drinking water (rain, surface, underground). Basic needs and volumes of water intake for human consumption and the consequences of dehydration.
3	Waterborne and faecal-oral contamination: sources of contamination, drinking waterborne contamination and infection pathways, protection of water sources for good health, barriers for faecal-oral infection route and the potential role of SODIS as a barrier to transmission
4	Safe water storage and transport: cleaning drinking and storage vessels and protection/covering of storage containers and the advantage of SODIS combining safe storage, transport and cover.
5	Diarrhoea-pathogen relationship: sequels of diarrhoea, handwashing in relation to personal-, food- and drinking water handling incl. Cleaning SODIS bottles
6	Videos: Animated SODIS promotion cartoon (www.sodis.ch). Showing popular movies to attract poeple to the community events
7	Mini-projects in support of SODIS-application: i.) small garden (gardening and watering plants is like caring for a child), ii.) small trade (buying and selling food stuff for financing SODIS-women events), iii.) water system maintenance: cleaning existing gravity water systems, iv.) community-soccer tournament
8	School events: Training pupils to use the SODIS manuals and SODIS comic books; perform participative role plays, and viewing SODIS videos (www.sodis.ch) to understand the role of SODIS in intervening in the diarrhoea-pathogen relationships, reducing waterborne contamination, and enhancing personal hygiene.
