# Supplementary material for: Solar Drinking Water Disinfection (SODIS) to Reduce Childhood Diarrhoea in Rural Bolivia: A Cluster-Randomized, Controlled Trial
Source: PLoS Med. 2009 Aug 18;6(8):e1000125. doi: 10.1371/journal.pmed.1000125 (PMC2719054; doi:10.1371/journal.pmed.1000125)
Supplement: Text S1 — Trial protocol. (0.52 MB PDF) [file pmed.1000125.s003.pdf]

Trial Protocol:

A cluster-randomized, controlled trial of solar drinking water disinfection (SODIS) to reduce childhood diarrhoea in rural Bolivia.
Acronym: BoliviaWET - Bolivia Water Evaluation Trial

Project Summary

More than one third of the population in rural and in peri-urban areas of developing countries has no access to sufficient or clean drinking water free of pathogens. Thus, waterborne gastroenteritis remains a major infrastructural and public health problem particularly, as effective treatment (filtration, chlorination, treatments plants) is often beyond financial means or environmental resources used for water purification (fire-boiling, burning carbon-based fuels) become scarce in those communities. In this context solar disinfection of drinking water is especially appealing using a combination of irradiation by direct sunlight and solar heating to kill the water-borne pathogens in contaminated drinking water. To date, the efficacy of the SODIS technology as a home-based, low-cost intervention to provide safe drinking water in low income countries is well established, and a large-scale promotion and dissemination program is under way in seven Latin American countries. The principal objective of this study is to evaluate the effectiveness of home-based solar water disinfection (SODIS) in reducing the burden of gastrointestinal illness in children under 5 years in rural villages participating in a country-wide Bolivian SODIS program. We will conduct a community (cluster)-randomized controlled trial following a cohort of children <5 in each community. Totally, 22 communities will be selected from among those districts designated by the country-program to receive the SODIS intervention. A pair-matched design will be employed where communities are first ranked according to their baseline incidence of diarrheal disease and the intervention then assigned within each of the 11 consecutive pairs of communities randomly to one of them. In each cluster, 30 children (660 in total) will be enrolled and followed up for 12 months. Data on diarrheal illness will be obtained from morbidity diaries kept by mothers and validated through weekly home visits. Stool samples will be collected during the baseline morbidity surveys and at times of a diarrheal episode in a child during follow-up. Water quality monitoring of raw water sources used for drinking water and of water samples after treatment with the SODIS device will be conducted systematically. Mothers of participating children will be interviewed at baseline and during the trial with regard to current water use, behavioral and environmental exposures of their child in the home and within the community. This study will specifically estimate; i.) how much of the efficacy of the SODIS technology established in laboratory experiments and in two tightly controlled phase-III trials can be retained as effectiveness i.e. under program conditions, ii.) the preventive fraction of all-cause child-diarrhea attributable to SODIS. In addition, pathogen-specific attributable risks of diarrheal illness will be calculated. The project is organised by the University of California, Berkeley, with its substantial experience in water intervention trials in US and it benefits from the tradition of North-South collaboration in public health research of the Swiss Tropical Institute, Basel, Switzerland. It is run jointly with the Universidad Mayor de San Simon which coordinates the Bolivian SODIS program..

Sites:

University of California (P.I. : John M. Colford, MD, PhD)
Swiss Tropical Institute, University of Basel, Switzerland (P.I.-.Subcontract.: Daniel Maeusezahl MSc PhD MPH
Centro de Aguas y Saneamiento Ambiental, Universidad Mayor de San Simon, Bolivia; (study site field office)

Introduction to revised application: Solar Water Disinfection: A Randomized Intervention Trial

(**Annual update 2004**)Sod-NIH-01-nihform-v8.doc, 2004,dm)

This is a resubmission of our proposal to evaluate the effectiveness of SODIS (Solar Disinfection), a simple, cost-effective point-of-use water purification technology that is based on solar radiation and that is applicable in developing countries as well as in disaster situations. We propose to conduct a community (cluster)-randomized controlled trial among 660 children below the age of five in 22 communities that participate in the country-wide SODIS dissemination program in Bolivia.

The present proposal has been adapted in various ways based on the suggestions previously made by the reviewers and our preliminary field study conducted earlier this year in Bolivia. We have made improvements in the development and cultural adaptation of the survey instruments, pretested informed consent procedures, piloted the training and operations of local field staff and developed procedures to successfully conduct home interviews and collect human fecal and water specimens for our microbiological investigations. We have retained the principal study design, study procedures and approaches to analysis which have been favorably commented on by the reviewers and present the strengths and motivation of this proposal below:

Unsafe drinking water remains a significant source of gastroenteritis in developing countries. Solar disinfection of drinking water has recently been identified as one of the three most promising low-cost interventions for the control of diarrheal disease (chlorination and hygiene improvements are the other two modalities) (World Water Day, 2001). The SODIS program provides an innovative and simple solution with the potential for a global impact particularly in the developing world. Although its efficacy has been proven in repeated laboratory tests by different groups (Joyce et al. 1996b, McGuigan et al. 1998, Reed et al. 2000, Wegelin et al. 1994) (Appendix H), its effectiveness when applied to communities has not been rigorously evaluated (Appendix B). This evaluation trial is thus proposed at a unique time. There is limited information available about the health impact of this method from two prior trials among an isolated Maasai community in Kenya (Conroy et al. 1996, Conroy et al. 1999). These trials do not provide sound and generalizable data on which to evaluate the health effects of SODIS under real world conditions; the Kenyan trials were all conducted in the same single community where tight cultural norms force participants to adhere exclusively to the treatment (SODIS intervention). Thus, the results from these closely controlled trials reflect ideal 'experimental' conditions, and ignore potential inconsistencies in human behavior (such as multiple sources of drinking water) and shortcomings that are expected in large scale programs and everyday life. We include the responses from WHO and the local government in Bolivia regarding this proposal that emphasize the public health significance of this trial (Appendix K). Such an evaluation is now timely because large-scale dissemination programs are likely to occur in the next decade. These dissemination programs could be modified or expanded based on the results of this proposed investigation.

Our recently completed pilot work in Bolivia, as well as the CDC-funded projects testing chlorination devices using a similar project design (Mintz et al. 2001, Quick et al. 1996, Quick et al. 1999) confirm that the proposed trial is operationally and logistically feasible in the rural Bolivian highlands of Totora District. In the US, our group has completed two prior randomized, controlled drinking water intervention trials and is currently leading two similar additional trials. All four of these studies were federally funded (CDC, EPA, and NIH) and competitively awarded. Through these four trials we have gained extensive experience in the design and management of large drinking water intervention studies. One manuscript of our work has been published, one is in press and two others are under preparation (Appendix G). Our group has published several other manuscripts related to drinking water research (Appendix G). Additionally, our group has broad prior experience in the conduct of international health studies.

One major development since our original submission has been the successful completion of a pilot study in Bolivia. The first goal of this pilot study was to refine and test our survey instruments based on ethnographical assessments of illness and disease perception, water management and risk behavioral patterns among the Quecha-speaking study population. Additionally, we conducted a cross-sectional baseline survey about drinking water usage among 142 families (with 868 family members) including water testing and stool specimen investigations. Recent diarrhea was reported in one of four children (24.6%). Stool specimen were obtained from 74 children (52.1%) of which 18 (24.3%) reported a current diarrhoeal episode. Parasitological and bacteriological stool diagnostics were successfully performed. A comparison of the water quality of community water sources used for drinking water and in-home water samples indicated a significantly higher pollution of in-home stored water for human consumption. This study demonstrated that our systems for coordinating interviewing, stool and water laboratory analysis were feasible in rural Bolivia, and generated support from the local governmental and public health authorities (Appendix K).

Below we provide summaries of our responses to the comments made by the reviewers after the original submission. Full details on the modifications and responses are provided within the body of the proposal.

Study design and the potential for confounding : For the control of confounding, we apply randomization at all levels (for the selection of villages and participants and for the allocation of the intervention). Additionally, we have matched villages on the pre-intervention outcome (diarrhea rate) as suggested by one of the reviewers. These two strategies (randomization and matching) are the most effective ways to reduce the variance of our effect estimate and to remove the potential bias due to confounding. In studying the effect of our intervention, confounding could result, as noted by the reviewer, if we differentially assigned treatment to those villages with either higher or lower inherent rates of diarrhea. However, by matching the villages on the baseline incidence of diarrhea the potential for confounding by differential initial conditions is greatly reduced. In further response to the reviewer, we now will further reduce the potential for mismatching villages by matching them on the basis of an estimate of diarrhea rates for eight weeks (previously based on a single 7 day recall diary) (Section D.6.b.). We will apply our newly adapted and pretested daily morbidity diaries combined with weekly home visits. This will reduce any misclassification bias in the pairing of villages prior to random assignment of the intervention. Based on our own experience in Bangladesh and Bolivia (Appendix F and F2) and that of others (Samanta and Van Wijk 1998), we will assess village characteristics that could be related to compliance by recording factors such as existing collective community efforts (e.g. commitments to community development, income generating projects, community banking accounts, cultural factors), to adjust for them in the analysis stage.

Ability to detect a reduction in disease incidence: We have clarified our presentation of impact measures. Because of our choice of outcome measure, specifically the absolute reduction in the annual incidence of child diarrhea (episodes/child/year), our study is powered to detect a smaller reduction (1.67 episodes/child/year) than that detected in the Kenyan trials (2.1 epi/ch/yr). These calculations are detailed in the body of the grant (Section D.8.). An important difference in the presentation of the impact measures is that the Conroy study in Kenya focused on the two-week period prevalence of diarrhea whereas the outcome for our study will be annual incidence rates of diarrheal. Conroy observed a 9.3% rate reduction in their Kenyan under-5-population comparing incidence rates in control and intervention groups (58.1% *vs* 48.8%) (Conroy et al. 1999). By transforming the Conroy data on two-week period prevalences into a reduction of the annual incidence for child diarrhea (episodes/child/year) their findings suggest an average reduction of 2.1 episodes/child/year for a child in the intervention *versus* the control group (12.1 *versus* 14.8 epi/ch/yr). This reduction in absolute number of annual episodes of diarrhea compares favorably to the 1.67 episodes/child/year that our trial is designed to detect. The difference in relative magnitude of reduction between the studies in Kenya and our proposal, thus, derives from the different baseline rates of childhood diarrhea in the two settings.

SODIS intervention in control communities and impact of negative results : Because the SODIS method has previously been shown to be highly efficacious (Joyce et al. 1996b, McGuigan et al. 1998, Reed et al. 2000, Wegelin et al. 1994) an important question arises about withholding the intervention in control villages. The intervention is not being withheld, however, because of the trial design but rather because of the framework of the Bolivian National SODIS Dissemination Program. The trial is taking advantage of the natural experiment that occurs as the intervention is introduced. The reviewers raised a valuable concern that a delay of the intervention in control communities may compromise participation rates. Our preliminary works among eight villages in the area showed that the population is well informed and in agreement with the two-year development plans. We neither found evidence for resentment concerning villagers' willingness to collaborate nor did they respond by pleasing the study team.

The Latin American SODIS Program is the first large-scale endeavor to promote and disseminate solar purification of drinking water in eight Latin American countries during an initial phase of five years (Appendix B). The program's secondary aim is to gain necessary experience to promote the SODIS methodology worldwide subsequent to this initial phase. Although our trial could have only limited impact on the continuation of this initial 5-year program, we believe that trials such as ours will provide the evidence needed for WHO and the international community to enforce or withdraw their current strong support of solar water disinfection as an innovative technology (Word Water Day, 2001, Appendices K) before further expansion. The result of this trial will be shared with the SODIS program management and the Government of Bolivia, - both are part of the study team (Section C4 and Appendix K)-, to allow for rapid decision taking. The publication and dissemination of results stemming from this trial can have a significant impact on the further promotion of this low cost technology or on the selection of alternate modalities.

Evaluation of compliance and process monitoring: Monitoring compliance of the use of SODIS is vital part in this proposal and was not made explicit in the previous submission. In response to the reviewers' suggestion we detail three approaches to evaluate compliance and determinants of use of SODIS in the body of the text. In brief, specific observations and questions were added to the existing survey tools that are administered during the weekly household visits (e.g. presence/absence of SODIS bottles on the rooftop, ability to offer SODIS water for refreshment). Secondly,

we developed during the pilot work a prototype of a radiation-sensitive, color-fading marker that measures cumulative exposure of bottles to sunlight in short- (14days), medium- (3mth) and long-term (12mth use) as a proxy-measure for sustained use (Appendix F). Thirdly, "defaulters", those who abandon the use of solar disinfection, will be surveyed individually. Similarly, repeated group discussions with groups of both users and defaulters will provide insights in motives and determinants (supportive and negative) of the SODIS method (Appendix J). These series of individual case studies and group discussions will provide information on rejection, dissatisfaction and motivation for the SODIS method and thus, generate valuable information to improve the effectiveness of the SODIS Program.

Cost and cost effectiveness analysis: In response to the reviewers' suggestions we have developed and detailed in the body of the proposal a cost effectiveness assessment with the aim to estimate the unit cost per episode of diarrhea averted by SODIS (Section D6e). This entails assessing direct (e.g. medication, care) and indirect household costs (e.g. time lost from work) as well as performing a cost description of the net savings per episode of diarrhea averted. This will allow us to generate a specific measure of economic and social impact of the SODIS intervention. In preparation for this formal cost effectiveness analysis of the SODIS method in the previous six months we completed a cost-of-illness study in which we were able to include a preliminary cost analysis among villagers who partake in the Bolivian National SODIS program. According to these estimates, which also include an assessment of the monetary value estimate of the time spent to undergo the training and formation we calculate an annual cost range per family for lowest and highest lifespan of the bottles between $1.20 and $2.10. Based on this experience we have developed cost monitoring with questions in both the weekly surveillance and the four-monthly home interview instruments (Appendix J).

Microbiological testing for specific stool pathogens : The collection and microbiological testing of random sample of 220 children at baseline and at first subsequent diarrheal episode is an essential component in this project. We have incorporated one reviewer's suggestion to repeat microbiological testing of stool specimen of the entire subsample at the mid-point of the project and will thus, be able;- i.) to assess the prevalence of asymptomatic bacterial, viral and parasitic infections at two different seasons, ii.) to describe the proportion of diarrhea attributable infection in the different treatment arms, and, iii.) to estimate the pathogen-specific attributable risk for diarrhea. In our pilot study in Mizque we successfully obtained stool samples of 80 children and completed microbiological testing in 74 (92.5%) of them. Protozoal and helmintic infections were recorded in 48.6% (36/74) (*G.lamblia* 33.8%, *E.hist/dispar* 23.0%). In 18 children (24.3%) a current diarrheal episode was reported. Bacterial infections were found in 5 children (*Salmonella* sp 2x, Shigella 1x, *E.coli* 2x). Infection status (protozoa and/or helminths) did not correlate with report of diarrheal illness (OR=0.3, CI-95%=0.08-1.1). In our trial, results from the individual stool investigations will be correlated with the data on symptoms from the health diaries and direct interviews and data on environmental and in-home water quality.

Multiple transmission pathways and their effects on estimates of the preventable fraction: The enteric pathogens that will be identified in this study can be transmitted to humans through multiple pathways (e.g., food, person to person, and water). These transmission pathways are interdependent. It is important to assess the impact of improving water quality through SODIS in the context of the multiple pathways of disease transmission. The data collected in this trial will allow us to examine the preventable fraction estimates due to SODIS use, by developing and analyzing mathematical models of disease transmission in the context of the 22 villages under study. Our group has extensive experience with such disease transmission models and will incorporate the field data into such a model. Several of our publications for such disease transmission models are included in the appendiceal material (Appendix G).

Budget and Personnel : This project involves teams at three sites: UC Berkeley, the Swiss Tropical Institute (STI), and University of San Simon in Bolivia (UMSS). Scientific research and administrative staff are needed at all three performance sites and the team's combined and complementary expertise has been favorably commented on by the reviewers. All paid scientific staff of the Swiss Tropical Institute (STI) and the Bolivian staff (who are on the STI payroll) will spend considerable amounts of their working time on site in Bolivia. The data management will be done at UMSS, most of the analysis will be performed at UCB and STI. A disease modeling expert (Dr. Eisenberg) from our UC Berkeley team was added to the project. Based on the reviewers' comment administrative costs at the UC Berkeley site and the STI were reduced and the Bolivian-based team was strengthened with a communication specialist and a local water engineer who will be permanently based on site.

A. Specific Aims

Diarrheal diseases are recognized as the cause of a tremendous burden of illness in developing countries. Despite this recognition, the annual burden of gastrointestinal disease is estimated at 4 billion episodes and results in 3.2 million deaths in children worldwide (Bern et al. 1992, Ford 1999). The burden of waterborne gastrointestinal illness has been estimated at 1.2 billion episodes per year (Ford 1999). In Bolivia (where this present proposal to evaluate a simple, low-cost solar-disinfection (SODIS) water treatment modality through a randomized, controlled trial would be

carried out) one of six rural children experiences diarrhea at any moment in time (Esrey 1996). Throughout Latin America, as elsewhere in low-income countries, the supply of drinking water is precarious in rural and low-income peri-urban areas. The gravity and urgency of the problems arising in the developing world from the lack of safe drinking water may explain why many previously attempted interventions were widely applied even before their clinical or public health effectiveness had been demonstrated rigorously.

Numerous locally adapted water interventions and low cost technologies to provide safe drinking water from treatment plants in low income countries have been proposed and tested (Cairncross 1989). At the household level, water disinfection methods such as chlorination or boiling have been promoted (Blake et al. 1993, Quick et al. 1996, Quick et al. 1999). However, limitations in the supply of chlorine, scarcity of firewood or high costs of fuel contribute to the fact that a substantial part of the rural and peri-urban population will continue to drink contaminated water unless affordable and effective in-home interventions are identified.

We propose to formally test an affordable and efficacious technique of in-home drinking water treatment that could, if applied widely, significantly reduce the burden of waterborne diarrheal diseases in many part of the developing world. This technique relies on solar disinfection of water and will be referred to hereafter as SODIS. The SODIS method is simple, inexpensive, and effective. SODIS uses solar energy to inactivate or destroy pathogens present in drinking water. The method consists of filling translucent bottles with water and exposing them to the sun for approximately five hours (Appendix A). The water is disinfected through the combined action of ultraviolet radiation and heat. The efficacy of the methodology was established originally through extensive laboratory experiments (Joyce et al. 1996b, Sommer et al. 1997, Wegelin et al. 1994). SODIS has been field tested for its technical feasibility and cultural acceptance in nine different countries (Hobbins et al. 2000a, Wegelin 1998). SODIS was shown in a well controlled randomized trial in a secluded African community to reduce diarrheal incidence both in children under five years of age and in adolescents (Conroy et al. 1996, Conroy et al. 1999, Conroy et al. 2001). SODIS has undergone nine years of development and intensive laboratory and field testing, including that of Conroy *et al.*'s field trial in Kenya (1996). Today, the Latin American SODIS Promotion and Dissemination Program in eight Latin American countries will provide the first large scale intervention program in four South American countries (Bolivia, Ecuador, Paraguay, Peru) and four Central American countries (Nicaragua, Salvador, Honduras, Guatemala) for the next five years. (Appendix B). The existence of this promotion and dissemination program provides a unique opportunity to rigorously evaluate the ability of SODIS to reduce the burden of waterborne infectious diseases.

High efficacy (i.e. the desired result of an intervention under ideal conditions) does not necessarily imply high effectiveness (intended benefit observed in "real world" situations), and there is a need to examine closely the steps that lead to effectiveness in the community. It is crucial to assess how much of the efficacy of SODIS established in previous experimental studies and limited field trials will be retained as effectiveness in the real world. The findings of the proposed trial will influence decisions regarding the extent and method of future dissemination of SODIS. This evaluation is timely since plans for large scale implementation of the methodology are being readied in Bolivia (Appendix C) or are now considered by major donors such as UNICEF in Bangladesh or ministries of health, such as the government of Bangladesh in the Bangladesh Arsenic Mitigation Water Supply Project (BAMWSP)(The World Bank 1998).

We propose to evaluate SODIS through a cluster-randomized, controlled trial in the Cochabamba area of Bolivia. The trial will be conducted in 22 villages in which the families of 30 children under the age of five years will be enrolled in each village. Both the villages and the children within them will be randomly selected from census data. The annual incidence of diarrhea will be measured in the 22 villages (11 pairs) where the SODIS technique will be introduced. Daily measurements (recorded each week) of diarrheal episodes will be made by community-based field staff. Analysis will be conducted with mixed effects models adjusting for the clustered nature of the data. In addition to the measurement of the incidence of disease, we will also collect specimens of stool and home drinking water for extensive measures of pathogen occurrence as was done in our recent pilot studies in Mizque. In particular, our estimates of viral waterborne diarrheal diseases in this setting will represent new information since viral disease occurrence has received little attention. The data will allow us to estimate the relative contributions of specific viral, bacterial, and parasitic pathogens in the etiology of diarrheal disease in this community-based setting. A trial of this kind has not been conducted previously in Bolivia or Latin America. This distinguishes our study from the few prior local hospital-based studies on the etiology of diarrheal disease (Ise et al. 1994, Lopez et al. 1989, Townes et al. 1997, Utsunomiya et al. 1995).

The enteric pathogens that will be identified in this study can be transmitted to humans through multiple pathways (e.g., food, person to person, and water). Our team, as well as many others, has shown that these transmission pathways are interdependent (Eisenberg et al. 1996). For example, increased transmission through poor hygiene coupled with poor sanitation may result in increased contamination of the water source. Alternatively, a water treatment program

implemented in a community with poor hygiene may have little impact on incidence, because individuals will be exposed to high levels of pathogens from other transmission pathways. To examine the effects of this pathway interdependency we propose to further utilize the data we will be collecting for the intervention trial to develop mathematical models that will provide estimates of the preventable fraction for the different conditions identified in the 22 villages under study.

Relevant and recent background work by members of our proposed tri-national (Switzerland, Bolivia, United States) research team have included:

- demonstrating the feasibility of an active diarrhea morbidity surveillance system using trained community fieldworkers (as would be done in the proposed trial) (Appendix F);
- successful monitoring of a large cohort of children in a remote setting;
- pilot testing of data collection instruments, quality control mechanisms for data management, and other operational logistics (Appendix J);
- successfully achieving full Institutional Review Board approvals for waterborne disease intervention trials as well as in infectious disease research in a variety of domestic and international settings.

The importance of waterborne gastrointestinal illness throughout the developing world, the existence of a cheap and effective intervention (SODIS), the concurrent limited dissemination program for SODIS, the need for a controlled evaluation of the effectiveness of SODIS under actual field conditions, and the experience of our tri-national collaborative research team in successfully conducting large scale drinking water intervention and observational studies in both the United States and the developing world encourage us to propose the following randomized controlled trial in which our specific aims are to:

- Evaluate the hypothesis that SODIS reduces the incidence of gastrointestinal illness in 660 children under the age of five years in rural Bolivia that are randomly selected from 22 villages ;
- Define, through an extensive microbiologic testing component, the baseline rates of pathogen-specific diarrheal illnesses and the pathogens responsible for the differences in diarrheal illness between active and control groups;
- Document the actual use and acceptance of SODIS by participants in the study;
- Assess the cost-effectiveness of SODIS and the social and economic impact of SODIS at household level;
- Examine through mathematical disease modelling the effects of the presence of multiple transmission pathways within a village on the preventable fraction estimate due to the introduction of SODIS.

B. Background and Significance

The importance of microbiological safety of drinking water has been recently reviewed with a global perspective that emphasizes the importance of waterborne transmission of disease both for the developed and developing countries (Ford 1999). This review leaves no doubt about that i) diarrhea is the leading cause of *morbidity* worldwide and is the fourth leading cause of *mortality;* ii) approximately 1.2 (30%) of 4 billion episodes of infectious diarrhea annually occur from contaminated water; and iii) this illness burden affects approximately 80% of children under the age of five in the developing world (Murray and Lopez 1997). These figures indicate the urgency for sustainable mitigation of waterborne disease risk and explain why numerous locally adapted water interventions and low cost technologies to provide safe drinking water in low income countries have been proposed and intervention programs been launched. Many of those interventions, ideally, would only widely be applied at a population level after their clinical and public health effectiveness had been proven. In the case of SODIS, although extensive laboratory testing proving its efficacy has been carried out (Joyce et al. 1996b, Reed et al. 2000, Wegelin et al. 1994), only few community-based studies on the health effects of the method have been conducted to evaluate its effectiveness (Conroy et al. 1996, Hobbins et al. 2000b).

This background section briefly:

- describes general concerns about drinking water and the specific situation in Bolivia;
- introduces the SODIS methodology for solar disinfection of drinking water;
- reviews recent studies of low cost domestic water treatment interventions;
- indicates the significance of gastrointestinal infections in Bolivia; and
- provides the rationale for the selection of the specific pathogens to be investigated in this study.

B.1. Concerns about drinking water situation in general terms and in Bolivia

More than one billion people depend on potentially contaminated surface water sources (such as rivers and streams) (World Health Organisation 1992). The census of 1992 estimates that only 54% of the Bolivian population has access to safe drinking water. This situation was accentuated during the 1997/98 El Niño-induced drought which caused a 50% to 60% reduction in the available surface water for the department of Cochabamba (Sarmiento 1998). Provision of a reliable year-round supply of drinking water remains a great problem for most of the populations in low income countries, as effective treatments (filtration, chlorination, treatments plants) are often beyond the financial means of the community (Huttly et al. 1997, Sommer et al. 1997). Similarly, other means of purification such as boiling water for drinking by burning wood or burning carbon-based fuels outdoors or indoors in poorly ventilated dwellings carries other adverse financial, economic and health effects. For example, a recent review on indoor air pollution and acute lower respiratory infections indicated a clear dose-response relationship between exposure to biomass fuel exposure and the risk of acute respiratory infections (Smith et al. 2000). Due to the geological setting, the situation is particularly dire in the Altiplano region of Bolivia. In these highlands, with elevations between 2-4000 meters, gravity-dependent water systems can often not be provided and the possibilities for drilling wells are limited. A study showed that in 95% of samples from either natural sources or delivered water samples, there were at least 10 fecal coliforms/ 100 ml (WHO standard guidelines are 0 FC/100ml, (World Health Organisation 1985)) (Romero et al. 1992). Even in major Bolivian cities, such as La Paz, Santa Cruz and Cochabamba, water supplies are not reliable due to intermittent chlorination and risk of infiltration of the distribution systems. Studies from neighboring Peru or Equador confirm that chlorination in municipal water systems was insufficient and contributed to the spread of *Vibrio cholerae* (Ries et al. 1992, Swerdlow et al. 1992, Weber et al. 1994).

In this Bolivian context, representative of so much of the developing world, solar disinfection of water is especially appealing because it is a low-cost, home–based water treatment method using a combination of irradiation by direct sunlight and solar heating to kill the waterborne pathogens in contaminated drinking water. Solar disinfection is applicable for both the 84% of the population that is rural and without adequate safe water supplies as well as for the 16% of the population that is urban and may temporarily be exposed to contaminated municipal water sources.

B.2. SODIS Solar disinfection of drinking water

Solar water disinfection (SODIS) provides a simple, efficient and sustainable drinking water treatment option. SODIS is a simple technique essentially consisting of a disposable translucent plastic bottle of 1-2 liters volume in which water is treated. (Appendix A). It is a point-of-use water treatment method that avoids secondary water contamination that commonly occurs through storage (Mintz et al. 2001). Disinfection is achieved by the combination of solar radiation and solar heating (McGuigan et al. 1998, Wegelin et al. 1994). The synergistic effect of radiation of UV-A and light of wavelengths of 320-450nm inactivates and destroys pathogenic microorganisms present in the bottle. In areas of full sunshine, bottles blackened half on the bottom enhance thermal absorption.

Recent studies in the (high altitude) Andean region characterized by increased UV radiation indicate the same efficacy of the methodology even without a dominant thermal component (Brofferio 2000, CdA 1997). The synergistic effects of radiation and temperature over an average of 5 hours of sunshine (temperature increase to 55°C during mid-afternoon) have been shown to remove microorganisms including fecal coliforms, bacteria (*Vibrio cholerae)* (Joyce et al. 1996b, McGuigan et al. 1998, Sommer et al. 1997), *E.coli*, Enterococci (Reed 1997) Pseudomonas, salmonella sp.), viruses (rotavirus, enteroviruses, polio virus, bacteriophage f2) and a number of parasites (helminth eggs) and resistant parasite cysts (e.g. Cryptosporidia, *Giardia lamblia*)(Sommer et al. 1997, Zerbini 2000).

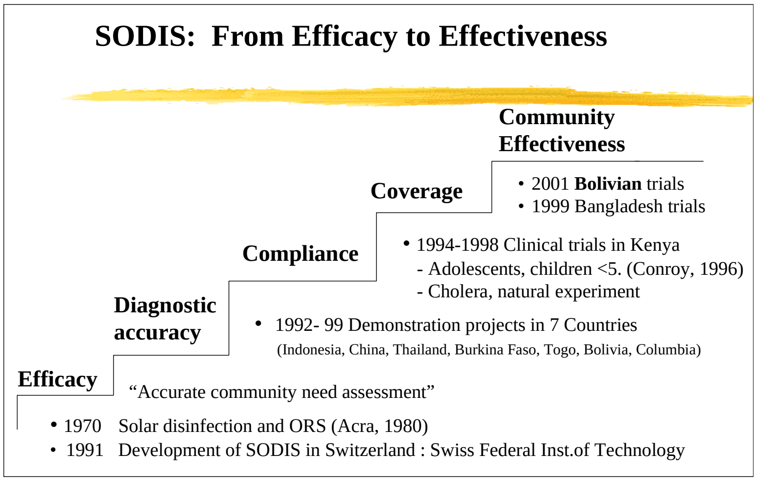

Figure 1 Development of the SODIS method

The SODIS bottles (translucent plastic bottles) are filled with water at the water source and exposed to sunlight at the home. SODIS water treatment occurs at the household (point of use) in small volumes of water for immediate use 'straight from the bottle' with no risk of

intermediate contamination through storage. The efficiency and acceptance of this methodology in different cultural settings has been proven in seven demonstration projects in Central and South America, Africa and Asia (Figure1.) www.sodis.ch, (Mintz et al. 2001, Wegelin 1998).

The evaluation of the health impact of such a simple, low cost water treatment technology and its effectiveness in reducing waterborne diseases at community level is both timely and essential at this stage where a large scale promotion in four South American and four Central American countries is planned in the next five years (Appendix B, C) (Wegelin and Sommer 1998).

B.3. Recent studies of low cost domestic water treatment interventions

Numerous technical designs and solutions such as protected community wells, pumps and communal standpipes have been developed and introduced to provide clean drinking water to rural and peri-urban communities in low income countries (Cairncross and Feachem 1993, Quick et al. 1999, Reiff et al. 1996). The inherent weakness in these approaches is the high chance of contamination on the way from the source to the home (Ahmed et al. 1998) or through inadequate storage at home (Mintz et al. 1995, Myo Han and Moe 1990, Vanderslice and Briscoe 1993). A study in Peru during a recent cholera epidemic that compared mean fecal coliform counts from household-stored water samples found levels that were one-thousand-fold higher than those found in water from municipal taps (Swerdlow et al. 1992). These results are similar to those we describe in our pilot study for this project (Section C1). Mintz and colleagues illustrate in their review how traditional drinking water storage vessels from various cultural settings in Africa, the Americas and Asia contribute to the spread of disease and showed that narrow-mouthed vessels consistently prevented secondary contamination and the spread of disease (Mintz et al. 1995, Reller et al. 2001, Roberts et al. 2001). Subsequently, their group developed a point-of-use water treatment method that relied upon chlorination of a 20 liter water container with a narrow mouth and outlet (Quick et al. 1996). The group successfully tested the device in a randomized study in 127 households of two native communities in El Alto, Bolivia and reported significant reduction of mean number of diarrheal episodes for children <1 year old and adolescents (5-14 years) (Quick et al. 1999). Although their interventions appear to be promising, a large scale implementation is hampered by i) the need for production of special storage containers and ii) its dependency on local production of sodium hypochlorite, and iii) the community distribution of the disinfectant. A 'real life' intervention on a larger implementation scale is planned to test its community effectiveness. The authors' proposal to investigate the social marketing strategies of chlorine and local vessel production as well as community mobilization to document the efficacy of the approach have not yet been evaluated (personal communication, R. Quick, CDC). Although this strategy reduces the risk of secondary contamination, i.e. it moves the source closer to consumption, the sustainability of the approach may be hindered by the several intermediate steps that this chlorination method requires (Quick et al. 1999).

No such intermediate steps are required in the application of SODIS (previous section). Nine years of development have been invested in the SODIS idea to prove its efficacy. What remains undone is a rigorous evaluation of its actual effectiveness (Figure 1). Preparations for the first multi-country dissemination program for the promotion of SODIS have started in Bolivia in 2000 (Appendix C) (See section B.5. and C.2.). This first large scale programmatic step was preceded by a number of demonstration projects and two clinical trials by Conroy's group in Ireland (Conroy et al. 1996, Conroy et al. in press). Other demonstration projects in nine countries, including Indonesia, China, Thailand, Burkina Faso, Togo, Bolivia, Columbia and Bangladesh, confirmed both the water users' interest as well as the acceptance and affordability of the technology in different socio-cultural, economic and environmental settings (Hobbins et al. 2000a, Wegelin 1998, Wegelin and Sommer 1998). These studies also indicated that widespread promotion and marketing was needed to build up local expertise and raise awareness of SODIS among different actors in the water sector at national and international level. SODIS is not yet well-known and understood by policy makers. In 2001, SODIS was officially recognized by WHO as one of the most promising low-cost interventions for diarrheal disease control (www.worldwaterday.org/report).

No clinical trials were performed before Conroy *et al.* conducted a series of three trials among children in a secluded Maasai community in Kenya between 1994 and 1998. In their first randomized controlled trial with 206 children aged 5-16 years they reported a statistically significant 10% reduction in the incidence of diarrhea and a 24% reduction of severe diarrhea in the solar-intervention group. The endpoint (presence or absence of diarrhea in the last 14 days) was verbally assessed using a semi-quantitative measure (Number of bowel motions "more than fingers on one hand"); no distinction between episodes was made (Conroy et al. 1996). A subsequent and bigger one-year study among Maasai children <5 years showed a similar reduction of 9.3% of all cause-diarrheal illness; a higher reduction rate was expected because of the known higher rate of symptomatic waterborne infection occur in children under 5 years (Conroy et al. 1999). With the occurrence of a cholera outbreak in their study area in 1997/98 the group was able to demonstrate the efficacy of solar water disinfection for cholera prevention in children below the age of 6 years

(OR=0.12, CI-95%=0.002-0.65) but not in adolescents or adults. To obtain information on cholera morbidity a retrospective assessment was employed using vernacular terminology to describe cholera-like diarrhea (Conroy et al. in press). Throughout their trials the investigators were not able to substantiate their outcome (verbal reports of diarrheal illness) by microbiological investigations. However, water quality testing of the surface waters used for solar disinfection was performed and the occurrence of waterborne bacteria, viruses and parasites in stool specimens in children <5 years was observed in a pilot-study (Joyce et al. 1996a).

The Kenyan trials demonstrated both the adequacy of solar disinfection and the plausibility of the effects within the small and tightly controlled Maasai environment. Our proposed study represents something quite different, specifically an attempt to measure the health impact under the actual program conditions under which the widespread dissemination of SODIS is planned. This will more closely reflect the effectiveness of SODIS upon integration into the public health system (Habicht et al. 1999, Tanner et al. 1993).

The previous preliminary small-scale experimental studies and trials described above served to develop the SODIS technology as a potent and efficacious intervention. However, high efficacy does not necessarily imply high effectiveness, and there remains a pressing need to examine closely the steps that lead to effectiveness in the community. It is thus crucial to assess how much of the efficacy of SODIS established in previous experimental studies as well as in the tightly controlled clinical field trials can be retained as effectiveness in the real world. Such an assessment is timely since plans for large scale implementation of the methodology are currently being readied in Bolivia (Appendix C) or are now considered by major donors such as UNICEF in Bangladesh and Bolivia and ministries of health (The World Bank 1998).

B.4. Significance of gastrointestinal infections in Bolivia; rationale for specific pathogen testing

General. Diarrheal disease account for nearly 20% of the deaths in children below five years of age in Latin America (Murray and Lopez 1994). In 1998, Bolivia developed a program for epidemiological surveillance as well as a health information system to detect changes in the state of health of the population. Data from a national survey from 1994 indicate a two week prevalence of diarrhea of 29.9% for children under five with little differences between urban and rural settings (30.9% vs. 28.7%). The same source reports a two-week prevalence of bloody diarrhea of 4.4% in urban and 7.7% in rural settings (p<0.0001) (ENDSA, 1994/S.I.N.S.). One local hospital-based empiric study indicated that 20% of patients presenting with diarrhea required admission and they estimated a diarrhea-attributable mortality of 7% (Ise et al. 1994). We obtained data for the Cochabamba region that indicate acute diarrhea incidence rates ranging from 16.5% – 37.8% in children under five which are consistent with these high rates (S.N.I.S, Systema Nacional de Informacion de Salud).

Bacterial agents. *Salmonella*, *Shigella*, *Campylobacter* and different strains of enteropathogenic *E.coli* are the most common causes of bacterial diarrhea: Only a small number of hospital-based studies report pathogen-specific findings: A study in patients from La Paz, Santa Cruz and Cochabamba investigated the etiology of bloody diarrhea and identified a bacterial agent in 41% of the 133 cases with *Shigella sp* being the most frequent agent diagnosed in 29% of the specimen, *Campylobacter* sp in 9%. *S. flexneri* was the most frequent of the isolates (72%). Enteropathogenic *Escherichia coli* strains were not investigated (Townes et al. 1997). The more comprehensive bacteriological study by Utsunomiya *et al.* in La Paz and Sucre isolated enteropathogenic *E.coli* strains in 18% of the cases (N=1234) and low prevalence of *Shigella* sp (6%), *Salmonella* sp (3%) and *V.cholerae* (3%) (Utsunomiya et al. 1995). In our pilot study for this project we found similarly low prevalences of these organisms, approximately 5% (Section C and Appendix F). These studies indicate the importance of bacterial infections among symptomatic cases. One local Bolivian community-based study confirms these findings and found a prevalence of 28% for bacterial agents in diarrheal cases with *Campylobacter* and enterotoxic *E.coli* (ETEC). This study also identified rotavirus in 6.2% of their sample. A study in neighboring Brazil identified enteropathogens in more than 50% of all symptomatic cases and in 17% of healthy control patients recruited from pediatric clinics (Seigel et al. 1996).

Viral agents. There are four major families of viruses associated with viral gastroenteritis relevant for this study setting: rotavirus, enteric adenovirus, astrovirus and caliciviruses which include Norwalk and Norwalk-like viruses. Viral gastroenteric infections are a leading cause of morbidity and mortality in young children in developing countries (Bern et al. 1992, Cunliffe et al. 1998). Rotaviruses are the principal known etiologic agents of severe diarrhea among infants and young children worldwide. In a recent published review of 43 studies on the etiology of diarrhea in developing countries rotavirus was detected in a median of about 24% in hospitalized and outpatient clients, more than 80% were children aged one. The same study noted that rotavirus is detected all-year-round with distinct seasonal peaks (Cunliffe et al. 1998). These figures compare well with the 22.5% detection rate of rotavirus in a Bolivian hospital-based study (Lopez et al. 1989) and a 42% prevalence among diarrhea cases in a local Cochabamba hospital (Dr

Panozo, unpublished report 1998). A study from an urban Brazil found rotavirus in 30.6% of the symptomatic cases but also in 13.7% healthy controls (Seigel et al. 1996).

- *Adenoviruses* are the second leading cause of severe gastroenteritis in small children (Bajolet and Chippaux-Hyppolite 1998, Prado and O'Ryan 1994, Ruuskanen et al. 1997). These viruses have not been researched much in the developing country setting although their transmission route does not differ from other significant viruses (such as rotavirus) and includes the fecal-oral, waterborne and that of small-droplet aerosols (Ruuskanen et al. 1997). Adenoviruses were found in 3% of asymptomatic children in South Africa (Steele et al. 1998).

- *Astroviruses* have been shown to be important etiological agents associated with gastroenteritis in children in developing countries (Steele et al. 1998). In the very few studies available astroviruses were identified in 7% of all acute diarrhea cases in South Africa and in 4% in a recent study in Bangladesh (Unicomb et al. 1998). The authors found a correlation between the detection of astroviruses and the duration of diarrhea. Astroviruses were found in 15% of specimens from patients with persistent diarrhea, 4% in acute diarrhea, but only in 2% of healthy controls. No studies have been conducted in Bolivia or Latin America to allow a comparison of the relative roles of these different viral agents or their role in the waterborne transmission of gastroenteritis.

- *Caliciviruses* (including Norwalk and Norwalk-like viruses). Norwalk like viruses (NLV) have been recognized today as a major cause of epidemic gastroenteritis in all age groups, and worldwide (Berke et al. 1997, Wolfaardt et al. 1997). Very little is known of the endemic situation in developing countries; Toranzos *et al*. identified NLV in all their samples of spring and surface water in a study in Cochabamba, Bolivia (Toranzos 1986). NLV appear to be responsible for about 4% of sporadic cases of gastroenteritis in South Africa (Wolfaardt et al. 1997). Today simplified PCR methods are available to identify NLV in water and stool specimens (primers developed by CDC).

Parasites. Parasitic agents are commonly associated with gastroenteritis and also play a major role in the developed world(Guerrant et al. 1990, Levy et al. 1998, Pruss 1998). Earlier studies in Bolivia indicated a very high prevalence of parasitized children among the indigenous population in high altitude areas (Basset et al. 1986) as well as in the tropical parts of the country (Cancrini et al. 1988). These studies consistently indicate the presence of protozoal infections of more than 80% of the children surveyed with multiple infections among 70% (Cancrini et al. 1988). *Giardia lamblia* and *E.histolytica* were very commonly found in more than 30% of the children with helminth infections being more frequent (91%, mainly *Ascaris lumbricoides*) in the Altiplano region (Basset et al. 1986) than in lower laying areas (20%, ascaris and trichuris) (Cancrini et al. 1988). More recent community-based studies confirm these high parasitic rates in under fives, particularly ascaris infections (42%) and giardia infections (23%). In our pilot study, we found 54% of 83 Bolivian children with at least one parasitic infection.

B.5. The SODIS Program and rational for the study setting in Bolivia

The Latin America SODIS Program: The main objective of this program is to promote and disseminate solar water disinfection (SODIS) on a large scale in eight Latin American countries within the next five years. (Appendix B). The implementation strategy is based on training and promoting local leaders to disseminate the method within the communities. The five year project aims to introduce SODIS as a household water treatment method for one million users. The implementation of the national programs will progress gradually from a pilot scheme initiated in Bolivia (Appendix C). Ample time remains for incorporation of lessons learned in our proposed trial before the widespread dissemination of SODIS.

Implementation of SODIS is demand-driven and based on a network of public and private institutions and organizations active in the water, sanitation or health/health education sector. In Bolivia, the network is called 'Red SODIS' (Spanish: network) and involves a large number of non-governmental organizations and private organizations in this first phase of the program implementation (Appendix C). PCI, has adopted the SODIS method as part of its community development program in water, sanitation and hygiene. It is within the framework of the PCI Program on "*Salud integral*" that we propose to conduct this trial (see also letter of approval, Appendix K)

The PCI Program on "*salud integral*": PCI is a US-co-funded NGO based in Bolivia since 20 years conducting community development programs (http://www.pci-bolivia.org). The current community development called "*salud integral*" consists of an activity profile including basic education, water and sanitation, mother and child health and health promotion in general, and agricultural production. PCI is intending to scale up its activities in the field of water, sanitation and hygiene education and has thus, teamed up with the foundation SODIS to joint the Bolivian national SODIS dissemination program. The Bolivian National SODIS Program provides the ideal conditions needed to conduct the first rigorous evaluation of the evidence that the SODIS technology works as an efficacious disease control tool to interrupt the transmission of water-related diseases at the community level. Together with the experience from the Kenyan trials this project would provide the data needed for the Bolivian (and, later, other governments) as well as large

donor agencies (UNICEF, World Bank, WHO) to embark on large-scale implementation of SODIS to reduce diarrheal disease burden.

C. Preliminary Work

C.1. Laboratory and field studies applying SODIS

During the past year our research group has completed several studies relevant to the proposed intervention trial. Significant milestones of our progress in developing the SODIS method as an effective point-of-use water treatment are briefly summarized below. Selected results are found in the Appendices E,F,G. They include:

i) laboratory radiation experiments on the inactivation of encysted forms of Cryptosporidia and *Giardia lamblia* (Zerbini 2000) and an assessment of the safety of radiation-exposed PET bottles (Wegelin et al. 2001); ii) field experiments on the efficiency of the SODIS method to inactivate waterborne agents in high altitudes in the Altiplano area of Sacabamba, Bolivia (CdA 1997), iii) pilot studies in rural Bangladesh using SODIS in a arsenic groundwater mitigation program in Bangladesh (Hobbins et al. 2000a), iv) our recently completed pilot work for this trial in eight villages in rural Bolivia (Appendix F and F2); and v) a pilot study for a randomized controlled trial of an in-home drinking water intervention in the US (Appendix G).

i.) Laboratory radiation experiments: Laboratory studies that illustrate the efficacy of SODIS to inactivate bacterial, viral and parasitic agents have been presented in section B.2. The two key publications of our group on the initial radiation experiments are attached (Appendix H). *Cryptosporidium* oocysts and *Giardia lamblia* cysts are notoriously difficult to inactivate and resist conventional water treatment (Payment 1999). In our recent study on the inactivation of the two resistant parasites we simulated natural irradiation and temperature conditions in a merry-go-round photoreactor. The results showed that 87% destruction of previously viable *C. parvum* oocysts will only be achieved after 10 hours of exposure to radiation levels twice that of sunlight. *G. lamblia* cysts, however, were inactivated at a rate of 83.5% after an exposure to full sunlight over 5 hours using the SODIS methodology which corresponds to the exposure of SODIS bottles over the mid-day hours. To quantify inactivation rates a fluorescence activated cell counter (FACS) was used. This study concluded that SODIS may not be efficient in destroying *C. parvum* but will effectively kill *G. lamblia* cysts after the exposure during two mid-day periods of full sunshine (Appendix E). A series of inoculation experiments underway in Bolivia will elucidate the effects of varying radiation levels at different altitudes and the role of oxygen in facilitating the inactivation process in the SODIS bottle. In another recently published study we were able to demonstrate that exposure to sunlight of PET bottles did not change the quality of the water. No photo-products or additives from the plastic material are released into the inside of the bottles during the photochemical aging of the bottles (Wegelin et al. 2001). Finally, for monitoring the use of SODIS, we developed during the pilot work a prototype of a radiation-sensitive, color-fading marker that measures cumulative exposure of bottles to sunlight in short- (14days), medium- (3mth) and long-term (12mth use) as a proxy-measure for sustained use (Appendix F). The fading is measured against a calibrated standard by field staff during each home visit. This information will be combined with interviews and group discussions on issues of compliance (see below iv.)

ii.) SODIS at high altitudes : A field experiment in the high altitude region of Sacabamba completed a series of feasibility studies in Bolivia (CdA 1997). This study conducted in seven large cities concluded that SODIS is applicable all over Bolivia. The minimum thresholds of 4000 Wh/m^2 mean monthly radiation needed for SODIS to work efficiently without the synergistic effect of temperature (i.e. when water temperature of 50 degree C. are not reached) is achieved throughout the year. (Appendix E).

iii.) Pilot study in Bangladesh : In 2000 we conducted a preliminary field study in 300 households of 15 villages in rural Bangladesh. This study was initiated to investigate the possibilities to introduce SODIS as a potential means to mitigate the prevailing arsenic crisis for the 20 million Bangladeshi who rely on groundwater supplies that are arsenic contaminated (concentration >0.05mg arsenic per liter (USEPA standards: 0.005mg/l) (Hug et al. 2001, Nickson et al. 1998). The objective of this project was to implement a community-based diarrhoea surveillance and to assess the feasibility to conduct a larger trial to test the effectiveness of SODIS. The experience of this study provided valuable insight about the acceptance, efficiency, practicability and health aspects of SODIS (Appendix F). For this purpose we conducted different monitoring programs from which we developed survey instruments (e.g. a weekly diarrhoeal morbidity monitoring form and an exposure assessment questionnaire) to measure covariates. We have demonstrated in this pilot study that we are able to establish a surveillance system to monitor the frequency of diarrhoeal episodes for all household members based on fortnightly visits to the households. Over 13 rounds of data collection interviewers were able to obtain data on diarrhoeal episodes from on average 89% of all the household members during each household visit. Water quality of raw water sources and drinking water samples from SODIS users and non-user households were routinely tested for fecal coliforms. The DelAgua kit, a portable field water testing

kit was successfully used for qualitative and quantitative assessment of fecal coliforms. It is a standard device used at the Centro de Aguas y Saneamiento Ambiental and will also be used in the proposed study.

iv.) Preliminary results from the pilot study in Bolivia : In our recently completed pilot study in eight rural villages in Mizque we applied similar approaches to field-test operations and logistics for this proposed trial. In particular, we tested the proposed procedures to obtain an informed consent (Section D.3 and E, Appendix M), surveyed 142 families with children <5 (total 868 individuals), and conducted water testing of water samples from household stored water and community water sources and, finally, conducted stool specimen investigations in a sub sample of the study participants. Based on the experiences made, we refined and tested our survey instruments that were developed based on the Bangladeshi experience (Section C3.).

- *Enrollment and consent* : For this pilot study we selected eight small villages that were typical for the area with varying proximity from the main road and different water supplies (river, spring, irrigation channel). We obtained consent from the local municipality and subsequently from the village leaders (dirigentes) before approaching individual families. The informed consent form and procedural protocol to obtain individual consent are provided in Appendix M.

- *Baseline characteristics* : We enrolled all families with children <5 years (N=142). Only two families declined from participating. The average household size was 6.2 (SD=1.9) and 54.3% of the families had more than one child below five. More than 75% of both parents had at least 5 years of schooling (primary school level); only 8.4% of the male household heads and 20.4% of the mothers were found to be illiterate (Appendix F, Table 1.). None of the families reported difficulties participating in the study.

- *Health outcome and stool laboratory* : In total, 35 (24.6%) of the children were reported experiencing an episode of diarrhea at the time of the interview. The frequency of reported diarrhea steadily decreased from 35.1% in infants (defined as <12 months for this analysis) to 0% in children below 5. (p=0.016) (Appendix F, Table below). We successfully obtained stool samples of 80 children and completed microbiological testing in 74 (92.5%) of them (see also Appendix F, Table 2/3.). Bacterial infections were found in 5 children (Salmonella sp 2x, Shigella 1x, E.coli 2x). Protozoal and helmintic infections were recorded in 48.6% (36/74) and less often in symptomatic cases than in asymptomatic cases (OR=0.3, CI=0.08 – 1.11). Diagnostic procedures may explain this finding: 94% of the stool samples of symptomatic children were recorded as liquid in the laboratory. However, same aliquots of liquid and solid fecal matter were used for stool microscopy rendering liquid and thus, diluted stool samples less likely for parasite detection.

Reported child diarrhoea and parasitic infection status of 140 children <5 years from Mizque, Bolivia; 2001

		Children's age (months)				
	**Total**	**0-11**	**12-23**	**24-35**	**36-47**	**48-60**
Reported diarrhoea status						
- Diarrhea	35 (25%)	**20** (35.1)	**12** (30)	**3** (13.6)	(0)	(0)
- No Diarrhea	105 (75%)	**37** (64.9)	**28** (70)	**19** (86.4)	**13** (100)	**8** (100)
**Total**	**140**	57	40	22	13	8
Stool diagnostics						
- *Entamoeba hist./dispar*	17 (23.0%)	**2** (7.4)	**6** (27.3)	**4** (26.6)	**2** (33.3)	**3** (60)
- *Giardia lamblia*	23 (31.1%)	**1** (3.7)	**6** (27.3)	**9** (60)	**3** (50)	**4** (80)
- Protozoa	34 (45.9%)	**3** (11.1)	**10** (45.5)	**12** (80)	**5** (83.3)	**4** (80)
- Helminths *	7 (9.5%)	**0** (0)	**4** (18.2)	**2** (13.3)	**1** (16.7)	**0'** (0)
- Any Infection **	36 (48.6%)	**3** (11.1)	**11** (50)	**13** (86.7)	**5** (83.3)	**4** (80)
- None	38 (51.4%)	**24** (88.9)	**10** (45.5)	**2** (13.3)	**1** (16.7)	**1** (20)
**Total**	**74**	27	22	15	6	5

* *Hymenolepis nana* (5) *Ascaris lumbricoides* (2) ** MH-Chi2 test of trend : p=0.0001

() column percent

- *Water quality :* Water samples were collected from seven community water sources (CWS) frequented by the villagers for the collection of drinking water and from 68 household water containers. Buckets, jars, canisters and clay pots were the predominant types of storage containers. The table below illustrates that water quality (measured as fecal coliform count (cfu) in 100ml water) varied by type and location of community water source (ranging from 0 – 34 cfu). Water quality of in-home water samples was significantly worse than that of community water sources with mean cfu counts ranging from 2.3 to 210. More than 58% (36/62) of all in-home water samples tested positive for fecal coliforms indicating a secondary contamination of drinking water during storage.

Comparison of Water Quality of Water Samples obtained from In-home and from Community Water Sources (CWS), Mizque; Bolivia

	In-home water samples					CWS *
Village	All measurements			Measurements > 0-cfu		
	Nr	Nr > 0 cfu	mean Nr cfu	mean Nr cfu	Std Dev	Nr cfu
Buenavista	14	8 (57.1%)	21.0	36.75	70.3	4 (river)
Centro Aguadita	6	4 (66.7%)	2.3	3.5	2.6	0 (spring-tank)
KewinaKasa	12	4 (33.3%)	14.2	42.5	62.1	0 (spring-tank)
Kuri (alto, centro)	8	5 (62.5%)	22.4	35.8	75.6	9 (river bed)
Vina Perdida	10	10 (100%)	209.6	209.6	476.4	34 (irrigation channel)
Lampesillos	9	2 (22.2%)	22.8	102.5	137.9	0 (spring-tank)
Yunguillas	3	3 (100%)	76.0	76	45.3	1 (irrigation channel)

( ) Percent cfu measurements >0 cfu /100ml

* mean of three repeated measurement per type of community water source (in brackets)

Water was reported to be collected on average 4 times per day, mainly in buckets (74.5%) and small canisters (17%) and more than 60% of all households were storing drinking water for more than 24 hours.

- *Ethnomedical concepts of child diarrhea :* We conducted a qualitative assessment to culturally adapt our instruments and to understand child diarrheal illness in the local Quecha context and to facilitate addressing the topic in our interviews. This exploration revealed nine different terms and local beliefs about etiology and treatment. “Kechalera” is the general term in the quechua language for liquid stool. Sub types specify bloody, mucoid and prolonged diarrheas (Appendix F, Table 3.). The ethnomedical terms are today considered as well defined entities in the traditional culture of the Quechua, but without a specific equivalent in the biomedical pathology. Ongoing analysis will determine those terms for inclusion in the survey instruments that most closely resemble biomedically defined diarrhea.

- *Monitoring the use of SODIS* : For monitoring the use of SODIS a radiation-sensitive, color-fading marker will be further developed as a practical technical monitoring tool (see above). In addition, SODIS users and defaulters, those who abandon the use of solar disinfection, will be surveyed individually and in groups to provide insights in motives and determinants (supportive and negative) of the SODIS method (Appendix J). These series of individual case studies and group discussions will generate valuable information to improve the effectiveness of the SODIS Program.

v.) <u>Water intervention trials in the US</u> : Finally, our group completed and published (in press) a pilot study that was conducted in the context of the recent debate in the US about the extent of waterborne transmission of gastroenteritis through municipal water supplies. This study was done to facilitate a larger randomized controlled trial and established that for the forthcoming trial, successful blinding of subjects was possible, and a blinding index of 0.66 (threshold value at 0.5) could be achieved (Colford *et al.* Appendix G, http://www.cdc.gov/ncidod/eid/vol8no1/colford.htm.) Additionally, our group is leading three other drinking water intervention (all are controlled, randomized, and blinded) trials in the United States: HIVWET (HIV Water Evaluation Trial among 100 HIV+ persons, field collection recently completed) in San Francisco; BIGWET (among a randomly selected population based sample of 1300 persons in Davenport, Iowa); and ELDERWET (among 600 seniors in Sonoma, California, enrollment began 9/01, 30 households enrolled to date). All of these studies were/are federally funded with no commercial support and were competitively awarded. Details of these studies are in Appendix G.

C.2. Development of data collection instruments

Draft data collection instruments for the first submission of this proposal were developed based on various forms from several previous field studies in Zimbabwe, Tanzania and more recently from our study on SODIS in

Bangladesh and in Bolivia. Final drafts of these instruments in English are currently developed incorporating findings of the 2002 study (Appendix F-2).These instruments will be submitted for IRB clearance at all study sites before their use in this trial. However, actual interviews are performed using the instruments written in local Quechua language (previous versions from 2001 in Appendix J). They were translated into the local Quechua language and back-translated to English to ensure accuracy of the translation and clarity of contents. They include a screening and enrolment questionnaire and five principal data collection instruments: i) a questionnaire for baseline assessment of socio-demographic characteristics of the study child and household including questions on water management and use; ii) a weekly observational protocol to monitor water, hygiene or sanitary conditions at the community and in the household; iii) a weekly follow-up questionnaire on gastrointestinal symptoms to be administered by the community-based field staff; iv) a questionnaire for assessing exposure risks at four different times during the study period; v) We also developed an illustrated household morbidity diary for self-administration and recording of diarrheal illness for the study child and household members. This serves to validate our weekly diarrheal morbidity assessment through a maternal interview. Similar participatory instruments have been successfully used previously (Stanton and Clemens 1987) and their validity and use in the application of diarrheal disease research has been advocated recently (Goldman et al. 1998, Kalter et al. 1991). A similar tool has also been successfully applied by our group in a recent study on gastrointestinal illness in HIV population in the US (Colford et al. Appendix G). These instruments have been shown to be at the same time appropriate for the use by locally trained personnel and capable of measuring the outcomes and exposures of interest. Questionnaires were developed using English templates from previous studies and were then discussed with the local Bolivian field team. Interviewers and data collectors were trained during a one-week workshop organized locally. Overall, our pilot study and previous experience demonstrated that with adequate personnel and with the instruments that we developed, data collection was complete, operationally and logistically feasible to obtain in a rural setting and over a prolonged period of time.

C.3. Study site and choice of study design

Study area and population: The trial will be conducted in Totora, a section of the province of Carrasco, one of the 16 provinces in the department of Cochabamba. The capital of this region is the city of Totora (approx. 1600 habitants). The communities involved in this project are situated in a radius of about 30 km around the city of Totora. These communities have an estimated total population of 13,000. They are situated in an area that is unsuitable for well sinking or gravity water supplies. About 20% of the rural population are children <5 years and 28.5% are 5-14 years old. The average family size is 4. The incidence of diarrhea in children <5 years of age is estimated from different national studies at 4-5 episodes/child/year. (S.N.S.I.)(Prado and O'Ryan 1994, Quick et al. 1999).

The SODIS Program in Bolivia: The SODIS diffusion program has been developed since 1997 through a number of experimental field studies on the efficacy and acceptability of SODIS in Sacabamba (CdA 1997). The SODIS Foundation and later in 1999 the Centro de Aguas y Saneamiento Ambiental and developed different educational materials such as videos, tapes with tales for children, flip charts for community awareness raising and booklets for household distribution (Appendix I) and radio broadcasts. PCI, one of the partners in the SODIS Network Bolivia, took active part in distributing the materials and introducing SODIS as part of their program on "*salud integral*". (See section B.5).

The Bolivian SODIS Program was launched with initial funding from the World Bank which is supporting the production of dissemination materials, and an award obtained at the Second World Water Forum, The Hague (Martin Wegelin, 2000). Full funding for the entire Latin American SODIS Program was received in Jan 2001 (Appendix B). Our proposed trial will be conducted within the area of operation of PCI /Cochabamba, one of the partners in the SODIS network. PCI is introducing SODIS in groups of communities continuously over a period of 24 months through trained technicians and community field workers and teachers. PCI is expanding its activities within the area of Totora and has agreed to collaborate with this project allowing a staggered, community-based introduction of the SODIS method. PCI provides us, thus, with the framework to adopt a "stepped-wedge design" for our community randomized controlled trial, where the intervention is introduced step-wise (Kirkwood et al. 1997, Smith and Morrow 1991).

C.4. Research team and network of collaborators

The proposed trial represents a substantial multi-disciplinary effort by teams from Switzerland, Bolivia, and the United States with well-defined roles based on each group's specific expertise in the design, conduct, and analysis of the project. The team from UC Berkeley has extensive field experience resulting from the design and management of the four federally funded large-scale drinking water intervention trials described in Section C1. Each of these trials is targeted to a different population (elderly, HIV-infected, or segments of random populations with no

immunocompromised). These separate studies are being conducted in Walnut Creek (immunocompetent population), San Francisco (HIV positive participants), and Sonoma (elderly participants), California as well as in Davenport, Iowa (immunocompetent participants from the challenged water source on the Mississippi River). The first of these studies (PILOTWET in Walnut Creek) is in press (Appendix G) and was released early by the journal on its web site to permit inclusion in an upcoming report to Congress (http://www.cdc.gov/ncidod/eid/vol8no1/colford.htm).

The Swiss team has a longstanding experience in applied public health research in developing countries. Members of our team have conducted various randomized controlled trial in the SPf66 malaria vaccine development (Acosta et al. 1999, Alonso et al. 1998, Alonso et al. 1994), the effectiveness of impregnated bednets (Binka et al. 1996, Lengeler and Snow 1996), and lately the effects of co-arthemeter in the treatment of schistosomiasis (Utzinger et al. 2000a). Diarrheal disease research and health impact evaluations of water and sanitation interventions studies in Burkina Faso, Cote d'Ivoire, Chad, China, Bangladesh and Zimbabwe, constitute another major field of the group's expertise (Booth et al. 1996a, Booth et al. 1996b, Cissé et al. 1999, Gagneux et al. 1999, Hobbins et al. 2000b, Mausezahl et al. 1996, Pitts et al. 1996, Shier et al. 1996, Tanner et al. 1993, Utzinger et al. 1999, Utzinger et al. 2000b).

The Bolivian group at the University San Simon (UMSS): Since a preparatory visit of the PI/STI in 2003 the Bolivian group is newly composed of the Centro de Centro de Aguas y Saneamiento Ambiental (CASA) at the technical faculty, the Centro de Estadistica Aplicada (CESA), a biostatistics support unit within UMSS, and the Laboratories for Medical Diagnostic (LABIMED) of the Faculty of Medicine.

The Centro de Aguas y Saneamiento Ambiental has a reputation in local water research and water quality monitoring of ground and waste water in the region and the evaluation of water quality of mobile water providers in the peri-urban areas of Cochabamba, (CdA 1999). Their work focuses on the removal of iron and manganese from groundwater for drinking purposes, the monitoring of heavy metal existence in the Ichilo and Caine rivers. Since 1996 their research has been on appropriate technologies for solar disinfection of drinking water (Brofferio 2000) and on the health effects of pesticides in soil and water.

The Centre for Applied Statistics (CESA) is a: unit developed by the programme of University Institutional Cooperation between UMSS and three Belgian Universities. CESA supports the UMSS faculties in planning the research activities and in analyzing and treating the data. The Faculties of Sciences and Technologies, of Medicine and of Agronomy of the UMSS are participating at the first phase of the project. CESA has two missions: i) training in biostatistics and research design and data management, and ii) statistical support to research activities within the UMSS.

Laboratories for Medical Diagnostic (LABIMED) is an operating unit of the Institute for Biomedical Investigations and Social Interactions (IIBISMED) of the Faculty of Medicine. The LABIMED laboratories were created in 1990 with the scope of providing the Faculty with high quality diagnostic laboratory services, facilitating teaching and scientific research. LABIMED is a regional reference laboratory.

As detailed above, our core team has extensive experience in waterborne disease research, the conduct and management of large cohort studies, intervention trials and clinical epidemiological research, and research in developing countries. Together with the direct link to the technical and engineering expertise from the Swiss partner who developed SODIS since 1991 (EAWAG) and with our Bolivian Partners at the University of San Simon (Brofferio 2000, CdA 1997, Sommer et al. 1997, Wegelin et al. 1994, Wegelin and Sommer 1998) the existing team can readily apply the experience in their fields to the currently proposed water evaluation trial in rural Bolivian communities:

John Colford, MD PhD, Assistant Professor of Epidemiology, University of California, Berkeley, School of Public Health and Principal Investigator for this proposal. He will have overall responsibility for the design and conduct of this trial. Dr. Colford was the Principal Investigator of a recently completed pilot randomized trial focusing on blinding of subjects in trials of waterborne diseases that is funded by the CDC-P and the USEPA (Appendix G). He is the Principal Investigator of three additional multi-year federal (US) drinking water intervention trials. He has been involved in state and federal water safety issues for the past six years with invited service on numerous expert panels and working groups. He is a physician (Johns Hopkins 1985) and is board-certified in Internal Medicine (indefinite), Infectious Diseases (through 2006), and has a doctoral degree in epidemiology (PhD, UC Berkeley, 1996). He is the sole instructor in advanced, semester-long graduate courses on epidemiologic methods at UC Berkeley, has directed year-long seminars on clinical trials at UC San Francisco, and teaches a semester-long course on Intervention Trial design at UC Berkeley. He has worked very closely with Dr. Mausezahl (the proposed Project Director) and Dr. Hubbard (the project biostatistician) over the past year in the design of the proposed trial while Dr. Mausezahl was a visiting scholar at UC Berkeley.

Daniel Maeusezahl MSc., PhD., MPH., is a Research Scientist at the Swiss Tropical Institute (STI), University of Basel, Switzerland. He is the PI of the subcontract to the STI and the Project Director for the entire project and will

provide the general oversight for the implementation of the project and its study design. He will have overall responsibility for planning, implementation and the coordination of the collaborating institutions of the project (UCB, STI and Bolivian Partners). For several years he conducted and led extensive applied field research on the impact assessment and evaluation of water and sanitation interventions and wastewater management Sub-Saharan Africa and Asia. In Zimbabwe his scientific work focused on developing a rapid district-based health impact assessment. His research in Burkina Faso and in China dealt with the waterborne transmission of diarrheal disease and hepatitis A. He also piloted the studies on participatory methodologies in water and sanitation that lead to the PHAST (Participatory Hygiene And Sanitation Transformation) component within the WHO "Africa 2000" Initiative. Since 1998 he initiated and directed several studies to investigate health effects of the SODIS methodology in Bangladesh and Bolivia. He gained experience in social and cultural epidemiology and illness behavior in a study that he directed in Switzerland on the health strategies of migrants and determinants of health care utilization; the study was funded by the Swiss National Science Foundation. A project development grant by the Nestle Foundation facilitated the recent pilot work in Bolivia.

Alan Hubbard, PhD is Adjunct Assistant Professor and Research Scientist at UC Berkeley, School of Public Health. He will be the statistician on this project. Dr. Hubbard has taught several graduate-level advanced methods courses at UC Berkeley and authored several publications on longitudinal data analysis. Much of his academic work has centered on methodological improvements in the analysis of data related to HIV+ patients, including improvements to survival estimates, accounting for delay of reporting, and quality adjusted lifetime data. For several years he has been statistical consultant for the California Department of Health Service's Reproductive Epidemiological Section, overseeing the statistical analyses of risk factors and their association with pregnancy and fertility outcomes. Several of these studies involved longitudinal data, such as a repeated measures analysis of menstrual function and stress. He has been the acting statistician in both the clinical trial and FDA approval phases of a California State Department of Health run study of treatment for infant botulism. His background in statistical applications in epidemiology and his expertise in longitudinal data has provided a sound background for the development of the proposed analytic strategies for this project.

Lee Riley, MD is Professor of Infectious Disease and Epidemiology, UC Berkeley School of Public Health). He has extensive (NIH-supported) experience in the conduct of microbiologic investigation in developing countries, particularly in the Americas. He is the U.C. Berkeley co-Investigator microbiologist on the project. He will help to guide all aspects of the collection and testing of human specimens for microbiologic analysis and conduct specific testing for enteropathogenic *E.coli* in his UCB laboratory. He is the senior author on a recent lead article in the *New England Journal of Medicine* describing the molecular epidemiologic techniques used in tracking a clone of *E. coli* in multiple locations in the United States (Manges et al. 2001)

Joseph Eisenberg, PhD MPH, Adjunct Assistant Professor, University of California, Berkeley, School of Public Health, is a bioengineer and a public health modeling expert. He will work with us to construct mathematical models of the different transmission pathways in rural Bolivia to estimate the potential impact of SODIS considering different transmission scenarios based on the empirical data from this project. Dr. Eisenberg has extensive research experience related to the epidemiology of waterborne diseases. He is currently the Project Director of our group's HIV+ cohort drinking water intervention trial at the San Francisco Veterans Affairs Medical Center (SFVAMC) and the water intervention trial using an elderly cohort in Sonoma California. He has collaborations with: 1) The CDC, studying the serological response of HIV+ individuals to Cryptosporidium; 2) The EPA, developing a microbial risk assessment framework from exposure to drinking water; and 3) The World Health Organization, developing a modeling framework to both estimate the global burden of disease contribution from water, sanitation, and hygiene. He has published numerous peer-reviewed articles on infectious diseases modeling (Eisenberg et al. 1995, Eisenberg et al. 1998, Eisenberg et al. 1996)

Thomas Smith, MSc., PhD. is Senior Scientist and the Head of the Biometrics Unit at the Swiss Tropical Institute. He has statistically directed and overseen the antimalarial and malaria vaccine trials in Tanzania and Papua New Guinea and the insecticide-treated-bed net trials in Ghana. His special research interests are in the spatial analysis of malaria transmission and molecular epidemiology of plasmodia infections. Dr. Smith will act as a consultant statistician /epidemiologist, making use of his expertise in tropical epidemiological research to complement the work of Dr Hubbard. His responsibilities will be to advise the principal investigator on details of the design, on optimization of procedures for logistics and data management, and to consultant with Dr Hubbard at the stage of data analysis.

Monica Daigl, MSc. environmental scientist, graduated at the Swiss Federal Institute of Technology (Zurich). She is the research and project assistant to the PI/STI at the Swiss Tropical Institute; she directed a field study on environmental risk factors for diarrhea and malaria in an urban context in Côte d'Ivoire. This study was done within the framework of a Swiss National Science Program on Health and Well-being in Urban and Sub-Saharan context in Africa.

Andri Christen, MSc. Doctoral graduate student in epidemiology at the University of Basel. He is a biologist - medical parasitologist by training with particular expertise in surface water biology and laboratory diagnostics of Noroviruses. He participated in the first Swiss national study on the occurrence of waterborne gastroenteritis. He is the Swiss project coordinator on this project resident in Bolivia and will be responsible for the day-to-day operation of the project including the coordination of all field activities in Bolivia. He is replacing Michael Hobbins who was foreseen as assistant project manager in the 2000/1 submissions of this protocol

Ana Maria Romero Jaldin, Lic., MSc., is the head water microbiologist and on the permanent staff at the Centro de Aguas y Saneamiento Ambiental (CASA). She is a senior lecturer at the Faculty of Science, University of San Simon in Cochabamba, Bolivia and Vice Director of CASA. She is supervising a series of laboratory studies on environmental inorganic and microbiological pollutants and on radiation patterns and their effects on biota and humans in Bolivia. Her specific research focuses on the health effects of heavy metals in sediments and chironomids. She will be primarily responsible for the organization, coordination and supervision of the laboratory components regarding water analyses and stool specimen diagnostics. Together with the Assistant Project Director and the Programme Coordinator 'SODIS Bolivia' she will directly guide the field implementation of the trial protocol. She will have the overall responsibility for the trial's activities at CASA. She will specifically supervise and assure data quality for all water and stool diagnostics and direct all research activities relating to water quality at the Centro de Aguas y Saneamiento Ambiental. She will work closely together with the project's microbiologist on all laboratory aspects regarding stool investigations.

Mercedes Iriarte, MSc. is the water microbiologist and on the permanent staff at the Centro de Aguas y Saneamiento Ambiental teaching various courses on laboratory techniques. She is currently supervising a series of studies on the detection of *Cryptosporidium* and *Giardia* in domestic wastewater and continues with our previous studies on the inactivation of encysted stages of parasites resistant to irradiation. Other work pertinent to the study involve investigating the effects of altitude and the role of oxygen on pathogen inactivation rates in water. Together with the team at CASA, she developed and conducted the pilot studies on SODIS in the Andean region of Bolivia since 1996, - studies that facilitated the current SODIS program. She will be responsible for the organization, coordination and supervision of the laboratory components regarding water analyses and stool diagnostics.

Max Zarate MSc, PhD is a native Bolivian trained as a Water Engineer. He obtained his PhD in Environmental Health Sciences at UC Berkeley. His previous research experience led him to an appointment as Chief Public Health Officer in the local government of Cochabamba where he was responsible for the cholera control and outreach prevention programs. He has extensive scientific and managerial skills along with substantial experience with disease control in the local Bolivian setting. He will help to guide all of the water sampling and testing done during the study.

Martin Wegelin, MSc is the Program Officer and Coordinator of the world-wide SODIS Promotion and Dissemination Project at the Department of Water and Sanitation in Developing Countries (SANDEC), the research department affiliated to the Swiss Federal Institute for Environmental Science and Technology (EAWAG). He was Lecturer in Sanitary Engineering at the University of Dar es Salaam in Tanzania for 3 years and has extensive experience in civil and sanitary engineering in developing countries. His special research interests are in water filtration technologies, solar water disinfection technology and arsenic removal processes. He recently won the first price with his work on the SODIS device and method (Solar Water Disinfection (SODIS) at the second World Water Forum, The Hague. He will advise our project on technical issues pertaining to the SODIS application and will provide the link to other SODIS Program components in other Latin American countries.

Marcel Tanner, MSc., PhD, MPH. is Professor of Epidemiology and Medical Parasitology and the Director of the Swiss Tropical Institute. He is a public health and communicable diseases specialist and directed numerous studies in the fields of malaria, on basic research on transmission, entomological and immunological determinants of clinical disease and the evaluation of different household-level control measures such as insecticide impregnated bednets. He guided the implementation of phase I/III malaria vaccine trials of SPf66. Other specific interests focus on schistosomiasis research on inexpensive community-based methods of identifying places where control measures are needed and morbidity control within existing programs and the epidemiology of health system utilization. He is a consulting member to the Swiss Academy of Sciences, WHO and TDR/WHO and among the board of directors of INCLEN and the Wellcome Trust Tropical Medicine Interest Group, as well as chairman of Tropical Medicine Advisory Board at the University of Heidelberg and the Board of trustees of the Ifakara Health Research and Development Centre, Tanzania.

Robert Quick MD, MPH, is a public health specialist at the Center for Disease Control, Atlanta, who has an invaluable research experience in conducting applied field research in the field of diarrheal disease control in The Americas. Together with his colleagues he implemented several studies on low cost water interventions and diarrheal disease control in Bolivia. He made significant contribution to the understanding of the epidemiology and the control of

the cholera epidemic in Latin America. Dr Quick will make his expertise from his local research available during the study and will provide an essential discussion platform for the interpretation of the results at the end of the trial.

Luis Villaroel, BSc, MSc, is the Director of the Centre for Applied Statistics (CESA) at the UMSS and will have the overall supervision of the activities of the CESA biostatistics unit incl. the work of Gonzalo Duran Pachenco.

Gonzalo Duran Pachenco, BSc, MSc, is a statistician with a biology background and training in Belgium in Statistic (Master in applied statistics at the Free University of Brussels). He will be responsible for the data entry and preparation of the data files and will work on data analysis along with the other project statisticians.

Maria Estrella Zapata, MSc, is the Head of the Department of Microbiology at LABISMED/IIBISMED. She will coordinate the activities at IIBISMED/LABIMED. With her collaborators, who are senior staff and Head of the Departments for Virology (**Patricia Rodriguez**, MSc.), and Dept. of Parasitology (Dr **Mary Cruz Torrico**, MSc., MPH) she will be responsible for the supervision of the project's virological, microbiological and parasitological specimen analysis.

C.5. Institutional support and approval

A number of different institutions are involved in the conduct of this study. The letters of support and approval letters from the relevant institutions (Swiss Tropical Institute, University of San Simon, PCI, Cochabamba) involved are found in Appendix K. The status of approvals for the protocol as of July 2003 is as follows;

i.) approved by the Berkeley Committee for the Protection of Human Subject, OHRP IRB number 00000775 (February 27th 2003)

ii.) approved by the Swiss ethical research board in Basel (Ethikkommission Beider Basel) OHRP IRB number 00003565 (March 11th 2003), Annual Ethical Review on March 14th 2004

iii.) approved by the University of San Simon IRB, Bolivia OHRP IRB number 00003509 on April 19, 2004

iv.) The protocol and has been read and approved by local partner institutions including the Fundación SODIS and Project Concern International (PCI)/Bolivia and will next be presented to the local government at the Municipality of Totora District. The letters of institutional clearances are attached (Appendix C).

D. Research Design and Methods

D.1. Overview of the design

The principal aim of the proposed study is to evaluate the ability of a simple, low-cost, home-based water treatment method based on solar disinfection to reduce gastrointestinal illness among children less than 5 years of age in rural Bolivian communities. To achieve this objective we will conduct a pair-matched, cluster-randomized controlled trial following equal numbers of children in each of 22 villages (Appendix L). The intervention to be tested is a method called SODIS (<u>So</u>lar <u>Dis</u>infection) which makes use of the synergistic effects of solar radiation and thermal heating from sunlight. The SODIS Program, by design, disseminates the intervention to whole communities, rather than to individual households or single individuals. The major steps in the randomization process are:

1. 22 villages (from a total of 60 possible villages) will be selected for enrollment from the Totora District;
2. each village will be paired with another village with a similar incidence of baseline diarrhea, as suggested by the reviewer previously;
3. within each pair of villages, one village will be randomly selected for the "treatment" arm and one village for the "control" arm.
4. within each village all families will receive the SODIS intervention. A random sample of 30 children under 5 years of
   age will be enrolled in the study (total of 330 children in each arm of the study, 660 children total for the study). In families with multiple children the youngest child will be enrolled.

Please note that study introduces no delay into the receipt of SODIS by the control villages because control arm communities will receive the SODIS intervention at the end of the trial according to the scheduled implementation plans of the SODIS Program.

The 660 children (all <5 years old at enrollment), will be followed and monitored for the occurrence of diarrheal diseases over a period of 12 months. Data on diarrheal illness will be obtained from daily morbidity diaries kept by mothers/caretakers (*) and through weekly home visits by our project staff. Participating mothers/caretakers of participating children will be interviewed at baseline and repeatedly during the trial with regard to current water management, behavioral and environmental exposures of their child in the household and the community surroundings.

(* Note: the closest caretaker of the child will be contacted as respondent only if the team cannot locate the mother after two unsuccessful visits to the home)

Stool samples will be collected from a random sample of 220 children during the baseline morbidity surveys. The same children will be asked to provide two more samples at the time of their first symptomatic diarrheal episode and after 6 months follow-up. Testing of the stool specimen will be done at LABIMED, University of San Simon (UMSS)/Medical Faculty.

Systematic monitoring of the water quality of raw water sources used for drinking water and of household water samples after treatment with the SODIS device will be conducted. Testing for fecal coliform contamination will be done using portable equipment. More sophisticated analyses for bacteriophages and parasites (*Cryptosporidium* and *G. lamblia*) will be done at the laboratories of the Centro de Aguas y Saneamiento Ambiental/UMSS.

D.2. Inclusion criteria

Subjects: Families with children <5 eligible for the study are eligible to participate if they:

i) live permanently in the village;
ii) consistently use unprotected drinking water sources;
iii) give informed consent;
iv) Children <5 years must be permanently living with their family. In households with more than one child <5 years all the children will be enrolled.

Village clusters are selected from village profiles available from PCI. They apply the following criteria to include communities within the SODIS Program:

i) the village has no treated or improved individual- or functioning communal improved water supplies ;
ii) the village anticipates no drinking water interventions other than those of the Latin American SODIS program (as documented by village water and sanitation development plans and discussion with the municipality or other NGOs;
iii) uniform support for study participation is expressed by the village leadership.

D.3. Informed Consent

The informed consent process is based on IRB-approved procedures for the international SPf66 malaria vaccine trials (Alonso et al. 1994). We have provided a detailed outline of procedures and a letter of consent which we have already piloted (Appendix M, see also Section E.). If the mother of the child is willing and eligible to participate the project staff will go through the consent form with the mother. The consent form will contain all information about the design of the study, activities involved when participating and potential risks and benefits. Signatures of both parents and all household members living permanently there will be required. If the parents agree to participate and have signed the informed consent form the enrollment questionnaire will be filled out at the participant's home (Appendix J). If consent forms cannot be completed during the first visit field staff will arrange for follow-up meetings. The closest caretaker of the child will be respondent only if the team cannot locate the mother after two unsuccessful visits to the home.

D.4. Definition of diarrhea (principal outcome)

An episode of diarrhea will be defined in this activity and throughout the trial as a 24 hours period with three or more non-bloody loose or liquid motions, or one or more bloody, loose or liquid motions and three diarrhea-free days between a new onset. This is in concordance with the WHO recommendations and recent research (Ahmed et al. 1994, Aziz et al. 1990, Morris et al. 1994) and allows the comparison with other studies on diarrheal diseases in developing countries. Concepts of diarrheal illness and disease differ within and between cultures (Pitts et al. 1996, Weiss 1988). For example, a nutritional program in Honduras neglected to use word 'empacho' in their instructions for correct feeding practices of diarrheal cases. The subsequent inclusion of the folk term significantly improved the program's impact (Scrimshaw and Hurtado 1988). Attempts will be made during the pilot phase to assess the cultural perception of diarrheal illness and to elucidate the folk terminology and basic local illness beliefs about diarrhea (Appendix L). We conducted similar studies in previous research to obtain and validate local folk terms for diarrheal illness that resemble most closely the biomedical definition for diarrheal illness mentioned previously (Mausezahl 1996, Pitts et al. 1996).

A start of a new episode of diarrhea will be defined as the first day of diarrhea that occurs after at least three consecutive diarrhea-free days. Similarly, the end of an episode is determined by the onset of three consecutive diarrhea-free days. Our choice of three rather than some other number of disease-free days is based on the work of Morris *et al* who modelled the sensitivity of different definitions of diarrheal episodes and described the definition we

have chosen as ideal in situations of medium to high incidence (specifically greater than six trigger events/child/year) allowing for different lengths of duration and within-child clustering (Morris et al. 1994). Persistent diarrhea is defined as an episode lasting longer than 14 days according to WHO standards (Anonymous 1988); diarrhea episodes of shorter duration will be defined as "acute".

D.5. Evaluation of the outcome measures

Evaluation of differences in the primary outcome measure between the active and control arms will be through relative incidence rates of gastrointestinal illness (total episodes of child diarrhea / total person-time of observation in the control group divided by the same measure in the active group) adjusted for the nature of the cluster sampling. Secondary outcomes include child-level outcome measures such as the total number of days with diarrhea, the mean duration of diarrhea, and the number of visits to health care providers caused by the diarrhea. We also will obtain information to allow an analysis using a combination of symptoms that describe 'highly credible gastrointestinal illness' (HCGI) (Payment et al. 1991) from our weekly diarrhea monitoring household interviews (Appendix J).

Outcome data will be collected through a surveillance system established to measure diarrheal morbidity through daily illness diaries kept by the mother/caretakers of the children, and through weekly home-visits by project staff. A standardized questionnaire will be administered covering the clinical signs and gastrointestinal symptoms of current illness. Environmental (household and village-specific characteristics) as well as behavioral traits (water and food handling practices in the home, presence of pets, latrine arrangements) will also be recorded. Weekly home visits result in a total of 52 environmental and morbidity recording sheets for each child in the study.

Stool specimens will be collected and submitted for microbiologic analysis from a sub-sample of 220 (33%) of the children in the study during baseline, at their first diarrheal episode. In addition, as suggested by the reviewer, the entire sub sample will also be re-examined at a fixed time (at 6 months of follow up). This will allow us to measure three additional pathogen-specific outcomes: the prevalence of asymptomatic infections; the proportion of diarrhea attributable to infection for each organism detected in the study; and differential (pathogen-specific) infection rates in intervention and control group.

D.6. Tasks and approaches

The tasks to accomplish this project are presented below. We further outline our procedures for acquisition and analysis of the study data in section D.9.

D.6. a. Enrolment of trial villages and study subjects from the rural area covered by the PCI program

Communities in the area earmarked to participate in the SODIS component of the PCI program on “*salud integral*” will be contacted by PCI and the Fundación SODIS. Twenty-two communities will be randomly selected from a list of approximately 50 villages designated to be eligible for SODIS implementation by PCI. For participation in this study, community leaders of the randomly selected communities will be contacted by the study team. This team will approach individual village governments in their communities, describe the study and solicit their participation. If the village leadership indicates interest in participating the team will provide the same information about the trial and associated procedures as is given in the individual consent forms: eligibility criteria for households and study children, design of the study, activities involved when participating, potential risks and benefits.

A list of households with children below the age of five years will be available from information on the village profiles collected for each village covered by the PCI program and from the planned initial screening survey. Children <5 will be randomly selected from this list and subsequently visited by project staff. If the mother/caretaker of the child indicates an interest in participating the project staff will go through the eligibility criteria with the participant using a prepared form (Appendix J). If the mother/caretaker of the child is willing and eligible the project staff will go through the consent form with the mother. The consent form will contain all information about the design of the study, activities involved when participating and potential risks and benefits. Signatures of both parents and all household members living permanently there will be required. If the parents agree to participate and have signed the informed consent form the enrollment questionnaire will be filled out at the participant's home (Appendix J). For illiterate participants, the consent will be ready to them and they will be asked to provide permission verbally in front of two witnesses (see also instructions for the consent form in Appendix M). If consent forms cannot be completed during the first visit, field staff will arrange for a follow-up meeting. All information collected from households which are not eligible or decline to participate in the study will be destroyed except for information about exclusion. This information will be used to assess potential biases that might occur from the selection procedure of study subjects. These data will be coded and recorded with no identifying information on the household.

D.6.b. Baseline morbidity surveys in the trial villages to estimate the period prevalence of diarrheal episodes in children <5 years and to facilitate randomization of communities

At the beginning of the study, a prospective eight week survey among households with children <5 years will be conducted in the 22 villages to estimate the proportion (period prevalence) of child diarrheal illness. The usually short duration of a diarrheal episode and the short incubation period for a new episode will allow us to estimate the incidence density of child diarrhea in each cluster (i.e. village-cluster incidence rate) at baseline. The main purpose of obtaining a prevalence estimate is to facilitate matching of study sites according to the occurrence of reported diarrhea in children. This change (previously a 7 day cross-sectional survey but now an eight week longitudinal measurement) was done in order to address a comment in the prior review about improving our strategy to minimize possible differences in confounding factors between villages at baseline. Sites ranked by eight week baseline diarrhea rates are then grouped in pairs and the introduction of the SODIS intervention is randomly allocated to one of them. Thus, 11 sites receive the intervention, 11 other sites serve as controls. This high level randomization of the village-clusters to the SODIS intervention is the single most effective strategy to limit bias in the estimation of the intervention effect (Murray 1998) (cf. also Section D.7.). PCI will adapt to the random allocation of the SODIS intervention and will start activities in the villages of the intervention arm after our baseline survey has been completed.

Additionally, stool specimens will be collected from a random sample of 220 children during the baseline morbidity surveys. In each community specimens from two children for every one-year age group will be collected (ten total per village). We will obtain one stool specimen at baseline and after 6 months, and also one specimen at the onset of their first diarrheal episode and submit it for microbiologic analysis. Stool analysis at baseline and mid-term will allow us to estimate the pathogen-specific infection rates in asymptomatic cases in both intervention arms. Microbiologic testing of stools of the same cohort will be repeated at mid-point of the project to obtain a repeated measure of infection status (regardless of disease status) at another season. By conducting microbiologic testing of the subsequent 220 symptomatic diarrhea cases we will be able to obtain rates of pathogen-specific diarrheal illnesses and pathogens responsible for the differences in diarrheal illness between intervention and control group (see section D.6g. Specimen collection and testing).

D.6. c. Determining the monthly incidence of child diarrhea in both arms of the study

This task will be achieved by monitoring diarrheal morbidity in a cohort of 30 study children (from different households) in each of the 22 village-clusters, (total of 330 children in each study arm, 660 children total). We will obtain information on our main outcome, cluster(village)-level incidence rates of diarrheal episodes through two monitoring procedures for the entire 12 month follow-up period:

1.) Morbidity diaries: Morbidity calendars will be kept by mothers/caretakers of the children. The use of morbidity diaries or 'illness calendars' has recently been reviewed and their usefulness and validity confirmed in prospective studies on child health (Goldman et al. 1998). The mother/caretaker will be given a simple morbidity calendar to keep daily records of the child's morbidity status. This chart was pre-tested among 11 households in our pilot study in Mizque and the graphical illustrations proved culturally valid. The number of bowel movements (from sunrise to sunset), color and consistency of the stool as well as specific symptomatic features of the diarrheal episode were recorded using colored stickers (Appendix J). The usefulness of the tools for monitoring child health has been taken up enthusiastically by mothers and quantitative data on diarrhea frequency were collected (Jul-Aug.2002) in one village.

2.) Weekly home visits by community-based fieldworkers. Specific inquiries about the occurrence of a diarrheal episode using a standardized questionnaire will be made at the time of the fieldworker's weekly home visit. We will use vernacular terms of locally defined diarrheal illnesses derived from ethnographic pilot investigations in Mizque. For each of those illnesses, we will assess indicators for diarrhea as objectively as possible. This includes frequency of bowel movements and the presence of other symptoms (fever, vomiting, dehydration). Signs of dehydration will be assessed by both the number of urinations in last 24 hours as well as by capillary refill time. The different outcomes of the study (see below) will, thus be obtained by 52 measurements during the 12 months of follow-up. The assessment of the impact of SODIS on diarrheal morbidity will be obtained from cluster-level (villages) and individual (child) level outcome measures:

Village-level outcome measures: a *cluster(village)-level incidence rate* will be obtained by dividing the total number of diarrheal episodes in a cluster (village) by the total observation time of the study children in each village. This represents an incidence density measure. Similarly, the *mean number of episodes* of diarrhea per village-cluster will be compared among study groups.

Child-level outcome measures: the *total number of days with diarrhea*, and the *mean duration of diarrhea, and the number of visits to a healthcare provider* will be used for a child-level analysis of the intervention effect. The impact of

SODIS in terms of reduction of severe diarrheal episodes with blood, and/or fever or persistent episode (>14 days) will be assessed in a separate analysis.

D.6.d. Estimation of diarrheal disease preventive fraction of child-diarrheal illness.

Applying the definition of Last, community effectiveness is defined in this setting as the impact of the SODIS intervention on the overall incidence rate of child diarrhea in the community (Last 1995). It can be expressed in two ways:
i.) The relative risk (the ratio of the risk of diarrhea in the intervention group to that in the control group). With this ratio the community protective effect (CPE = (1-RR) x 100) can be obtained which expresses the percent reduction in the overall diarrheal incidence rate as a result of the SODIS intervention.
ii) The number of diarrheal illnesses averted as a result of the intervention (attributable risk) is measured by the difference in incidence density rates between the two study groups. To obtain an absolute number of diarrheal illnesses prevented by the use of SODIS, we multiply the attributable risk with the number of diarrheal cases obtained from the cross-sectional study. (In this calculation we assume that the results from the 22 cross-sectional studies represent the frequency of diarrheal illness in the general population).

D.6.e. Assessing the cost-effectiveness of SODIS and estimating the social and economic impact of SODIS at household level.

We will use cost-effectiveness analysis to complete this objective. Cost-effectiveness analysis compares the net monitary costs of a health care intervention with a measure of clinical outcome or effectiveness such as mortality rates, life years saved or events averted.

The aim is to estimate the unit cost for averting 1 (one) episode of diarrhoea by SODIS. Further we will perform a cost description of the net savings for a household per episode of diarrhoea averted. Multiplying this figure with the preventive fraction obtained from Section D.e we will generate a specific measure of economical and social impact of the SODIS intervention.

Costs will be assessed from the perspectives of the household. Household costs for a diarrhoeal episodes will be determined through a convenient sample of 50 acute and 50 persistent/severe episodes. The following parameters of the participating households will be assessed: a.) *direct costs* for medication, care services, or transportation, and b.) *indirect costs* of caretaker-time lost from work from caring for the sick child, d.) *time lost* for transportation and e.) time lost for a visit (waiting and treatment). Daily logs will be maintained by the household to measure these indicators. We will use local estimates of hourly income for a economically productive women to estimate the hourly wage of the mothers/caretakers. Programme costs for the total episodes of diarrhoea averted by the intervention will include the following costs: a.) for community building/training for the use of SODIS, b.) for actual costs of the SODIS bottles and their handling (e.g. cleaning before distribution), c.) the distribution, and d.) replacement of broken bottles. Research costs will be excluded.

The economic costs of SODIS implementation will be summed over the two SODIS implementation periods (4-6 months (Appendix L)) of the two treatment arms of the trial. The bottles have an estimated life span of one year and will be treated as recurrent costs (i.e. they are not discounted). Financial costs derived from the programme budget as well as economic costs (including volunteer and donations) will be considered in order to a establish cost-effectiveness ratio for the SODIS intervention. The number of episodes prevented by SODIS will be the difference in the observed episodes in the intervention households and the control households from Section D.6.d.)

We will finally conduct a sensitivity analysis computing cost-effectiveness ratios of the SODIS implementation for different replacement costs of SODIS bottles (doubled and tripled per year, and discount rates varied from 3% and 10% which is in agreement with recent economic evaluation practice in low income countries (The World Bank 1993). The net cost per episode prevented will then be calculated by dividing the cost of SODIS implementation by the diarrhoeal episodes averted (cost-effectiveness ratio).

D.6.f. Modeling study to examine the effects of multiple transmission pathways on the preventable fraction estimate.

Due to the properties of the infectious disease process discussed in Section A, it is important to assess the impact of improving water quality in the context of the multiple pathways of disease transmission. We propose to examine the preventable fraction estimates due to SODIS use, by developing and analyzing mathematical models of disease transmission in the context of the 22 villages understudy.

We will develop a standard household-level stochastic model (Ball 1999) that can be parameterized for each of the 22 villages. Four basic transmission pathways will be modeled: within household pathways that account for person-

to-person or person-to-formite-to-person transmission occurring within the household; between household or community-level pathways that account for transmission in communal settings such as schools, play areas, and bathing sites; environmental-to-person pathways that account for exposure of pathogens from environmental sources such as food and water; and person-to-environmental pathways that account for transmission of pathogens to the environment due to factors such as poor sanitation.

This modeling study will be a comparative analysis across each of the villages in the study. To this end, model parameters will be identified for each village. For example, relying on baseline survey information, the villages will be ranked with respect to hygiene and sanitation levels. These rankings will be the basis for assigning parameter values for person-to-person and person-to-environment transmission rates respectively. Once the model parameters are identified for each village, preventable fraction estimates will be obtained from the model by comparing incidence estimates under the conditions of an intervention with incidence estimates under the conditions of no intervention.

D.6.g. Specimen collection and testing (stool and water specimens)

An important component of our proposal is the collection and microbiological testing of stool specimen from study children. Data collection on pathogen-specific infections will be generated to elucidate three issues of particular interest in the current setting : i) the prevalence of asymptomatic bacterial, viral and parasitic infections in rural Bolivian children, ii) the proportion of diarrhea attributable to infection in the different treatment arms, and iii) the pathogen-specific attributable risk for diarrhea.

Prevalence of asymptomatic infection is obtained through the microbiologic testing of the random sample of 220 children at baseline. We will calculate the proportion of pathogen-positive stool samples among those children that were not classified as diarrhea. To obtain the proportion of diarrhea attributable to infection test results will be pooled from all symptomatic cases identified at baseline and at the time of their first episode. Since this will partly involve repeated measurements of the same individual we will account for the possible intra-person correlation by applying General Estimation Equation (GEE) approaches in the analysis (Zeger et al. 1988) (see also section D.9.). We will also explore how individual pathogen might be related to the probability of diarrhea.

These specimens will be tested for common causes of gastrointestinal illness due to viruses, bacteria and parasites. Stool specimens will be collected at baseline from a random sample of the children under five (N=220) and again at their first symptomatic episode later in the study. At the reviewers suggestion we will repeat testing the same cohort (regardless of disease status) at mid-point of the project. Field staff will distribute stool collection kits in the morning and collect them in the evening from the households if the child cannot produce a sample at the time of the visit. They will store the specimen in cooler boxes at 4 degrees C. and transport them to the local research office where refrigeration is possible. From there they are transferred to the central laboratory in Cochabamba within 48 hours. Participants will be notified by the study team about the test results and treatment initiated upon consultation with the medical center. A comprehensive array of tests will be performed at the Medical Faculty of the University of San Simon in Cochabamba.

Bacterial testing will be done for *Salmonella, Shigella, Campylobacter jejuni, Vibrio sp* and *E.coli* using standard microbiologic and biochemical tests. After biochemical identification of *E.coli* from lactose and non-lactose fermenting colonies pathogenic strains of *E.coli* (EPEC, ETEC and EIEC) will be tested for by PCR using a method co-developed by one member of our research team (Prof. Lee Riley, UCB) (Tornieporth et al. 1995).

Viral testing include rotavirus, adenovirus, astrovirus and calicivirus (Norwalk-like virus). With the exception of rotavirus, no comprehensive study of the viral origin of diarrheal illness has been undertaken in Bolivia. We will use commercially available test kits that are highly sensitive and specific: IDEIA Rotavirus, DAKO DIA rotavirus kit, Adenoclone (Meridian) an enzyme immunoassay, astrovirus will be tested using IDEIA astrovirus, DAKO DIA. Norwalk-like viruses will be tested using RT-PCR with primers developed at CDC. RNA isolation will be done by commercial kit (QUIAMP) and identification done by gel-electrophoresis and Ethidium-bromide stain. Parasitological testing will be done using SAF-fixative (sodium acetate acetic formalin) (Yang and Scholten 1977) a method that is routinely used in diagnostic laboratories in Canada and Switzerland (Marti and Koella 1993). The method does not require differential staining and has field tested in numerous of our field work in developing countries (Mausezahl 1996, Utzinger et al. 1999). *Cryptosporidium sp* will be diagnosed using a test kit (Merifluor, Meridian).

Water quality monitoring constitutes the second important component. At the *community-level* we will monitor routinely one natural water source frequented for drinking water collection in each village. Testing for fecal coliforms will be done using a portable test kit (Del Aqua kit) that is used by the Centro de Aguas y Saneamiento Ambiental. The method allows one to quantify fecal coliforms using MPN (Most Probable Number). A100 ml probe is filtered through a selective broth (Oxid's lactose sodium lauryl sulphate) and colony forming units of fecal *E.coli* are counted (Lloyd and Helmer 1991). Given the frequent identification of *Giardia lamblia* in indigenous children (Cancrini et al. 1988,

Quick et al. 1999) we will test one main water source per community qualitatively for the presence of *G.lamblia* and *Cryptosporidium sp* using the Merifluor, Meridian test kit. To correlate our findings of the viral etiologies of child diarrhea we will also test community water samples for the presence of male-specific F+RNA-coliphages. Coliphages are not only a sensitive indicator for human fecal water contamination but have also been shown to be substantially better indicators for the occurrence of viruses in water than thermotolerant coliforms or fecal streptococci (Havelaar et al. 1993). These tests will be done at the Centro de Aguas y Saneamiento Ambiental who apply a modification of the method described by Grabow *et al.* (Grabow and Coubrough 1986).

At the *household-level* the presence of fecal coliforms in stored water samples will be routinely monitored in sentinel households in villages that rank very high and low in overall reported diarrheal morbidity as established at baseline. Water samples from the 220 households selected for the baseline assessment will be collected once again at the time of a symptomatic case of diarrhea in the household. Testing for coliphages in home-stored water will only be done if a viral infection was detected in the stool specimen.

D.7. Confounding factors

We will measure potential confounding factors. At the reviewers' suggestion we now include monitoring village characteristics that could relate to compliance such as measures of community coherence (collective projects, community bank accounts), peoples' attitudes and opinion about SODIS and motivations for its use. Additionally, we will monitor factors that might affect the diarrheal outcomes including secular trends beyond seasonal changes such as diarrheal disease epidemics in the area or any intervention-like activity in the area, regional or national level (such as media educational campaigns).

D.8. Sample size calculation

Sample size calculations were designed for the trial to detect a 33% absolute reduction in the annual diarrheal incidence rate in children below the age of 5 in SODIS users compared to non-users. We consider a reduction of the annual incidence of 5 to 3.33 episodes per child <5 as of significant public health importance (Bern et al. 1992). This absolute rate reduction (1.67 episodes/child/year) is even lower than what was found in the Kenyan SODIS trials (2.1 epi/ch/yr)(Conroy et al. 1999). Conroy observed 2546 diarrhoeal episodes among a total of 4382 fortnight observation periods (58.1%) in the control group (172 children). A total of 2210 episodes among 4323 observations (48.8%) were found in the intervention group (174 children) resulting in their report of a 9.3% reduction of diarrhoea in children below 6 years. By considering 26 repeated measurements of fortnight periods an approximation of the annual incidence, the Conroy findings suggest an average annual reduction of 2.1 episodes/child/year for a child in the control *versus* intervention group (14.6 *versus* 12.7 epi/ch/yr). The magnitude of diarrhea reduction we have powered the study to detect compares favorably to the achievements of other well designed interventions that influenced national diarrheal disease control programs elsewhere in developing countries (Huttly et al. 1997, Stanton and Clemens 1987).

In cluster randomized interventions, within cluster similarities may occur as a result of the spread of disease among cluster members. We have considered this aspect in the design and statistical analysis of this trial: within-cluster similarities decrease the effective sample size in comparison with that required when using individual randomization. We calculated the number of clusters based on different assumptions about cluster size (i.e. the number of subjects in each village and total person-time of observation) and varying dimensions for the coefficient of between-cluster variation of the true diarrheal disease incidence rates in each study condition. The difficulty in calculating sample size for cluster randomized trials is the process of obtaining an estimate of the between-cluster variation. The calculations presented below use baseline assumptions for within- and between-cluster variation of similar studies (Hannan et al. 1994, Simpson et al. 1995). (Table 1). We also illustrate in Table 2 how differing levels of between-cluster variation may affect sample size using procedures for sample size calculations presented in Hayes & Bennett (Hayes and Bennett 1999):

$$c = 2 + (z_{a/2} + z_B)^2 \text{ x } \{(R_0 + R_1)/ y + k^2 (R_0^2 + R_1^2)\}/ (R_0 - R_1)^2$$

where c = the number of clusters within each group, y = person-years of follow up in each cluster and k = coefficient of variation (sd/mean) of the true rates of diarrhea between the clusters (within each group). We assume that k is the same in both treatment arms. Without other data currently available, different (conservative) assumptions are made for the between cluster variation. More accurate estimation of the between-cluster variation (k) of the true rates of diarrhea in the clusters will be obtained from reports of diarrhea from the baseline morbidity surveys and will provide the basis for pair-matching of intervention and control villages. However, we can assume that the exposure to environmental risks of

waterborne infection is similar in the villages involved in this trial and thus, the within-village variation will be greater than the between-village variation with regard to the outcome. As a conservative approach we assume that the between-cluster variation (k) will be k=0.3 or lower.

Calculations presented below are based on an equal number of subjects being investigated in each cluster. This sample size calculation is conservative since it assumes that the data will be summarized at the cluster level prior to analysis. Analysis using mixed models making use of the within cluster information are more efficient than other approaches (Murray 1998).

Table 1: Number of clusters required per trial arm for different person-times of observation and different levels of between-cluster variation.
(Based on a 33.3% reduction of the annual child-diarrheal incidence from 5 to 3.333 episodes/child/year)

**Cluster size**	**Coefficient for between cluster variation**		
**Person years of observation**	**k = 0.1**	**k = 0.25**	**k = 0.34**
5 (5 kids for 12 mth)	7.7	10.8	15.9
10 (10 kids for 12 mth)	5.4	8.4	13.5
15 (15 kids for 12 mth)	4.6	7.6	12.7
20 (20 kids for 12 mth)	4.2	7.3	12.4
30 (30 kids for 12 mth)	3.8	6.9	12.0

Table 2.: Number of clusters per treatment arm for different reductions of the annual child-diarrheal incidence rate and different level of between-cluster variation.
(Based on 10 person-years of observation in each cluster)

**Reduction of mean annual incidence rate**	**Coefficient for between cluster variation**		
Person years of observation	**k = 0.1**	**k = 0.25**	**k = 0.34**
20% (1 episodes)	12.3	21.9	38
33.3% (1.67 episodes)	5.4	8.4	13.5
50% (2.5 episodes)	3.4	4.5	6.5

Design effect: Ignoring clustering in this trial would turn the trial design into that of a classical clinical trial with individual randomization. The required sample size in terms of person-years of observation (y) is calculated as,

$y = (z_{a/2} + z_B)^2 \ x\ (R_0 + R_1) / (R_0 - R_1)^2$, and

would result in 23.6 year of person-time of observation in each cluster and in a design effect of ~2.4. (23.6/10)

From those calculations we conclude the following:

Number of clusters and subjects per cluster: We assume that the incidence rate of diarrhea in the control villages remains constant at 5 episodes/child/year and that we require 80% power (z_a= 0.84) of detecting a significant difference ($p<0.05$; $z_{a/2}$ =1.96) if the intervention reduces diarrheal incidence by 33%. We will achieve a minimum of 10 person-years of observation in each cluster, which amounts to 10 subjects per cluster observed for the duration of 12 months. Finally, considering a conservative between-cluster variation (k=0.2) we would need 9 villages each in the intervention and control group. Based on previous experience in our Bangladesh trial (Hobbins et al. 2000a) we consider a drop out rate of one to two villages per study group and thus adjust final sample size to 22 villages (clusters); 11 in each treatment arm.

Finally, we have included 30 children in each village with the following justification:

i.) few marginal costs arise in investigating more children in each village;

ii.) this allows us to compensate for loss of follow up of individuals;

iii.) the study is powered to maintain its ability to detect even a 16% reduction of diarrheal incidence from 5 to 4.2 episodes at a between cluster variation of k=0.12 (cf. Table 2. detailed Calculations not shown).

Thus, overall 660 households, 30 in each of the 22 villages and each with an index child under the age of 5 years enrolled in the trial will be participating in the study.

The following additional information on different parameters was considered in the sample size calculations above:

1. About 15% of the rural population in the study area are children under 5 years.(Centro Desarrollo Agropec., 1994).

2. The incidence of diarrhea in children <5 years of age is estimated from different national studies at 4-5 (episodes/child/year (S.N.S.I.)(Prado and O'Ryan 1994, Quick et al. 1999).

D.9. Data management and statistical analysis

Descriptive data analysis will be performed and selected demographic and environmental characteristics such age, sex, tribe/religion, household size, socio-economic/educational status, and the availability of water sources will be presented to indicate the effects of randomization and comparability of the intervention groups.

Comparison of rates: Cluster level incidence rates are used as the unit of analysis to compare incidence rates between intervention and control groups. Given the cluster size exceeding N=10 Pearson Chi^2 test can be used to compare cluster or group level incidence rates (Donner and Klar 1994). The calculations of Pearson Chi^2 accounts for the degree of intracluster correlation and the variance inflation due to clustering (Klar et al. 1995).

Bias and Confounding: Randomization procedures throughout the hierarchical structure of this cluster-randomized trial should make intervention groups comparable at baseline at cluster- and village level. Any bias resulting from incomplete randomization or from monitoring specific village factors that relate to compliance as well as unpredictable outside factors (e.g. media) will be dealt with in the analysis.

Evaluation of effect modification will be achieved by including multiplicative interaction terms in a generalization of Poisson regression analysis adapted for the residual correlation of the total number of diarrheal episodes among children in the same cluster (see details below). The effects of well known factors that act as effect modifier such as the presence /absence of sanitary facilities in the compound, or previous exposure to health education will be considered. Preliminary assessment of interaction will be done by stratification and decision will subsequently be made whether to include interaction terms as indicators in the Poisson regression analysis.

Multivariate statistics: Simple comparisons of rates will be done using software that is designed to accommodate the complexity of a cluster randomization design (SUDAAN). Simple Poisson regression cannot be used to make inferences about the effect of treatment in cluster randomization trials since it is constructed on the assumption that the conditional responses of individuals are statistically independent. An extension of the Poisson regression, generalized estimation equation approach (GEE) will be employed (Liang and Zeger 1986). For these regressions, the number of episodes of diarrhea for each child will be the outcome and, although we will report the inference based on robust estimates of the variance, we will assume the correlation within a cluster to be compound symmetry in the estimation procedure. We can possibly reduce the variance of the estimate of the treatment effect by adjusting for both baseline risk factors measured at either cluster level (cluster size, SES) or individual level (e.g. age, sex) (Klar et al. 1995). This becomes particularly important if the distribution of these covariates, by bad luck, are not similarly distributed in the treatment and the control arms of the study. We will also explore the strength of possible effect modification by looking at the inference of multiplicative interaction terms discussed above. We will account for the fact that some children will have different total time at risk due to our definition of an episode discussed in Section D.3 by including an offset term in the regression models that is the natural log of the total time (in days) that a child was at risk of diarrhea. Finally, the regression model will return parameters that can be interpreted as the marginal ratio of diarrhea rates relative to the variable of interest. Specifically, the models will return an estimate of the ratio of diarrhea rates in populations that have the treatment versus those with the controls, all other factors (covariates) being equal in the two populations. If we find significant effect modification with treatment, then the model will return rate ratios specific to levels of the interacting variable(s), e.g., the ratio of rates in treatment versus control among populations with unsanitary facilities.

A secondary analysis will consider estimation of the attributable fraction of diarrhea due to specific pathogens. In this case, the outcome data is the presence or absence of diarrhea among the stool samples and the explanatory variables of interest are the presence/absence of particular pathogens. In this case, we have two levels of replication: 1) the possibility of more than one stool sample (diarrhea) per child and 2) 10 children (with specimen tested) within the

cluster (village). Given the very real possibility residual correlation among the replicates, we again will use a GEE regression approach. In this case, our outcome is binary (presence/absence of diarrheal stool sample) so we will use the GEE generalization of logistic regression. We will choose the variables to adjust for (and those to consider as effect modifiers) similar to that described above (note, that treatment now plays the roll of a potential effect modifier).

D.10. Critical assessment and possible limitations of the approach

The randomized controlled trial is the most powerful and bias-free means for describing associations between interventions and outcome. This design provides the most accurate and critical evaluation of the efficacy of interventions. Accurate data on efficacy allow the calculation of the number of cases (or deaths) averted by an intervention in a given population. This then can be combined with the cost of implementation of SODIS to obtain cost effectiveness estimates.

Study Design. There is no ethical way to conduct this trial in a blinded fashion. We considered use of a cross-over study design, which would virtually remove any intra-person variation of diarrheal outcome due to factors other than the intervention. We are using such a design in our 1300 person water intervention study in Davenport, Iowa (Appendix G). Due to the substantial reduction in variability, sample size requirements would also be reduced. However, we abandoned this design option given the short trial period of 12 months as well as ethical reasons that object to the withdrawal of SODIS from households once begun in those households (i.e. crossover from active to placebo arm could not be justified). We compensate for this by the use of an amply large sample size in our proposed parallel arm design.

Study duration: One strength of the study compared to the one comprehensive study on the chlorination of drinking water (Quick et al. 1999) is the length of follow-up period of 12 months. In contrast to Quick *et al*. this will allow to measure seasonal effects of the SODIS intervention alongside with the seasonal infection patterns of agents causing diarrheal diseases such as rotavirus.

Sample size: Given the relatively large size of the study with 22 clusters and given that randomization will be employed at all levels we hope that residual bias will be measurable. While randomization of even small numbers of villages to intervention and non-intervention group ensures equal probability of assignment for each group, it cannot ensure that all potential sources of bias will be equally distributed. Pending our baseline field work in the trial villages, we have to anticipate a heterogeneity of the occurrence of baseline diarrheal diseases (our outcome) among the different village-clusters. A simple random assignment to the intervention or control group is unreliable to achieve baseline comparability among the study conditions. This applies particularly if the number of clusters in each condition is relatively small, as is in our study (N=11 villages). We thus, employ an *a priori* matching of the villages according to the baseline incidence of childhood diarrheal illness before random assignment of the intervention to one of the pairs.

Diarrheal episodes as outcome measure: Our outcome measure (diarrheal episodes) is subject to the influence of numerous measurable and unknown factors particularly in developing countries. By incorporating the suggestion made in the earlier review for *a priori* matching on the endpoint (baseline incidence of diarrhea) we have attempted to control for the effects of a number of unknown confounding factors as well as some of the factors that are difficult to assess such transient risk factors. We will be able to adjust for any relevant confounding factors that might randomly differ significantly in the groups.

D. 11. Ethical Considerations

All of the communities will be covered by the PCI program in a staggered approach providing the setting of a natural experiment for the study. Thus, every household will have access to the SODIS technology by the end of the program. Each village will be informed about the date of the forthcoming implementation of the SODIS intervention, i.e. villages in the control arm of the trial will know and have agreed upon their "delayed-intervention" status. Compensatory actions or resentful demoralization due to differential treatment of intervention and control villages are thus not an issue of concern. The selected communities will be informed through their village government who meet regularly with the PCI representatives, about the background of the study in broad terms. The village government as well as the community representatives must have given their consent for the whole project. Children will be enrolled in the study following written informed consent of both of their parents (Appendix M). Withdrawal from the trial is possible at any time without any consequences for the child, family and household. All information obtained from the questionnaires will be treated as strictly confidential and will not be passed to any other person or institution outside the research team. All children identified during any survey as suffering from a severe form of diarrhea (fever and/or signs of dehydration present) will be referred to the nearest health facility. Health service delivery to the study participant is provided through the regular health care system. Treatment is provided free of charge and is organized through the office of the District Medical Officer (DMO) together with the district health team. The project team is not involved in any patient

care. However, as services of local health posts are often interrupted due to delayed or inadequate supply of drugs our project will make budgetary provisions to support the local health posts in the project area to ensure these services to the study participants.

E. Human Subjects

This protocol has been developed applying the principles of the Helsinki Declaration (last updated 1983) and adheres to the outline for Good Epidemiologic Practice of the European Epidemiology Group (1999). The protocol has been approved by the Berkeley Committee for the Protection of Human Subject, OHRP IRB number 00000775 (February 27th 2003), by the Swiss ethical research board in Basel (Ethikkommission Beider Basel) OHRP IRB number 00003565 (March 11th 2003) and by the University of San Simon IRB, Bolivia OHRP IRB number 00003509 (April 19, 2004). The protocol and has been read and approved by the local partner institutions incl. the Fundación SODIS by PCI/Bolivia. The letters of institutional clearances are attached (Appendix C).

E.1. Study population

A total of 22 rural Bolivian communities (villages), eleven in the intervention arm and eleven in the control arm, will be enrolled from the area of operation of the a local, US-funded NGO Project Concern International (PCI). The PCI program on community development disseminates the SODIS technology to communities unsuitable for well-sinking or gravity water supply in the Cochabamba area, Bolivia. The PCI Program disseminates the SODIS intervention to whole communities, rather than to individual households or single individuals. In total 660 children under five years of age will be recruited into the study; 30 subjects from each of the 22 communities. Participating households will be recruited during the initial project period and subjects will be followed for twelve months.

Study participants will be selected based on the inclusion criteria indicated below, but only if full parental consent has been granted i.) for the household to participate in the SODIS program and ii.) to perform all project activities including water quality monitoring in the household and specimen collection of children’s stools and iii.) to interview mother/caretaker (*) of the child. A consent form will be signed by both parents and all household members. (* Note: the closest caretaker of the child will be contacted as respondent only if the team cannot locate the mother after two unsuccessful visits to the home)

For this study we will adopt a community (cluster)-randomized controlled trial design. I.e., the implementation plans for the SODIS component of PCI program allows us to introduce SODIS in 22 communities in a random fashion determined by us. Twenty-two villages will be randomly selected from among those districts designated by PCI to receive the SODIS intervention. The SODIS intervention will be randomly assigned to eleven of the villages (the “treatment arm”) whilst the remaining eleven villages serve as controls for the period of the field trial (12 months). Control communities will receive the SODIS intervention subsequently to the trial’s end according to the implementation plans of PCI program.

A cohort of 660 children <5 years, 30 in each community, will be followed and monitored for the occurrence of diarrheal diseases. For the 12-months period of the trial, data on diarrheal illness will be obtained from daily morbidity diaries kept by mothers/caretakers and through weekly home visits by our project staff. Participating mothers/caretakers of the participants will also be interviewed at enrollment, and with a more detailed questionnaire twice during and once at the end of the study.

Stool samples will be collected from a random sample of children during the baseline morbidity surveys. The same children will be asked to provide one more sample at the time of their symptomatic diarrheal illness during follow-up. Water quality monitoring of raw water sources used for drinking water and of water samples after treatment with the SODIS device will be conducted systematically. Mothers/caretakers of participating children will be interviewed at baseline and repeatedly during the trial with regard to current water management, behavioral and environmental exposures of their child in the household and within the community.

E.2. Sources of research material

Data will be obtained through i.) a baseline socio-demographic questionnaire survey, ii.) a baseline diarrheal morbidity survey, iii.) weekly morbidity questionnaires, iv.) a home-maintained illness calendar, and v.) microbiological analysis of stool specimen.

E.3. Recruitment

Communities: (Note: Communities earmarked to participate in the SODIS component of PCI program will have been contacted by PCI prior to this study and will have already agreed to receive the intervention. PCI is one of the implementing agencies for the Latin American SODIS program.) For participation in this study, community leaderships of participating communities of Mizque province, Cochabamba Department, will be initially contacted by the study team, and representatives of the University of San Simon, and PCI. This team will approach individual village governments in their communities, describe the study and ask if they may be interested to participate in this study.

If the village leadership indicates interest in participating the team will provide the same details about the trial and associated procedures for each participant as in the individual consent forms: eligibility criteria for households and study children, design of the study, activities involved when participating, potential risks and benefits.

Individuals: Given the approval of village government, participating households with children under 5 years of age will be visited by a study team member. If an individual indicates an interest in participating the project staff will go through the eligibility criteria with the participant. If the mother/caretaker of the child is willing and eligible the nurse will go through the consent form with the mother. The consent form will contain all information about the design of the study, activities involved when participating, potential risks and benefits. Signatures of both parents and all household members living permanently there will be required. If the parents agree to participate and have signed the informed consent form the enrollment questionnaire will be filled out at the participants' home. If consent forms cannot be completed during the first visit field staff will arrange for a follow-up meeting.

E.4. Screening

Households with children <5 will be known from the PCI community profiles and from the screening survey. Thirty households will be randomly selected from this data base. These households will be visited by a member of the study team and the inclusion criteria assessed. To become a study participant a child under the age of five must fulfill the following criteria:

i.) the family must be living permanently in the village
ii.) both parents and all other adult household members must agree to participate in the study.

All the children <5 years resident in the household will be enrolled.

All information collected from households which are not eligible or decline from participation in the study will be destroyed except information on exclusion. This information will be used to assess potential biases that might occur from the selection procedure of study subjects. These data will be coded and recorded with no identifying information on the household.

E.5. Procedures

(Note: No instructions or additional training in application and use of the SODIS device on part of the project will be given in villages in the intervention arm. Standard introduction of the device through PCI and the Fundación SODIS is a prerequisite for this effectiveness trial and will not be altered for the purpose of the study.
In summary, the SODIS device will consist of a translucent plastic bottle, which is filled with surface water collected for drinking water. The exposure of this bottle to sunlight and radiation for five hours during midday is sufficient to reduce most waterborne micro-organisms by more than 99.% and does not effect the chemical composition of the water. The PCI program introduces the SODIS device to all villagers within a community.)

Once the household consents to participate in the study the mother/caretaker of the study child will be introduced to a simple calendar chart to record the occurrence of a diarrheal episode individually for the study child and all other household members on a daily basis. This form also includes questions regarding water consumption and water management at the household. Information on type of water used, location of water sources and temporal absence of the study child from home will be collected. A community based field worker will visit the household once per week and inquire about the occurrence of gastrointestinal discomfort using an array of symptoms. These questions will be only referred to the study child. He/she will also complete an observational protocol recording environmental factors in the community and the homestead (presence of animals, cleanliness of household yard, water storage).
The mother/caretaker of the study child will also be asked to participate four times in a questionnaire-based interview. Household-based information will be collected on socio-economic situation, water handling and consumption practices, and perception of water quality. Information referring to the study child include nutritional, behavioral (water contacts, playing), and sanitary (personal hygiene, stool disposal) aspects as well as questions on health seeking and perceived health of the mother/caretaker. No such questions will be asked about other household members.

An important component of this research is the collection of microbiological testing of stool specimen from study children with appropriate consent. These specimen will be tested for common causes of gastrointestinal illness due to viruses, bacteria and parasites. Stool specimens will be collected at baseline from about 30% of the under five population (approx. 250 samples) in the participating villages and later during the study from about another 250 symptomatic cases with a diarrheal episodes. Field staff will distribute stool collection kits in the morning and collect them in the evening from the households if the child cannot produce a sample at the time of the visit. They will store the

specimen and transport them to the local research office from where they are transferred to the central laboratory (LABIMED) in Cochabamba. The nurse of the local health facility will be notified about the test results, and he/she will then contact the participant and initiate treatment upon a consultation at the health facility.

Water quality monitoring constitutes the second important component. Water samples of stored and SODIS-treated drinking water (in intervention arm) will be collected from about 10 households in every community (about 30% of the study sample) every two months. In addition samples of the raw water sources reported to supply the households with drinking water will be tested. All households will have consented to water quality monitoring of household drinking water.

E.6. Benefits

There are no known direct benefits to subjects from participating in this research. No compensation or monetary incentives will be paid. One important indirect benefit is an increased awareness created through dealing with water issues in the household and the community context at the same time. We expect a strengthening and empowerment process with regard to water management taking place at community level. After the trial the entire population of the trial villages will have basic means at hand to improve the quality of their drinking water with a device whose efficacy has been previously established. It is not known to date, however, if this treatment will decrease incidence in any disease when applied under field conditions. It is hoped that the research will benefit society by determining whether the quality of this simple home water treatment device is enough to prevent transmission of infectious agents in drinking water.

E.7. Risks

There is minimal physical risk associated with this study. In no study so far has the application of the device resulted in an increased growth of pathogens resulting in an increased risk of infection. There is at worst no improvement of the microbiological water quality. Also testing of the effects of aging and of radiation on the material of the plastic bottles has not been shown to cause any detrimental health effects. At the end of the study the SODIS water treatment devices will remain in use or abandoned as per decision of the household, unless the study indicates significant adverse health effects associated with the use of the device. There are minor risks associated with stool handling procedures. Stool samples are collected only from children, whose stools are usually handled by the mother/caretaker regardless the study. Collection of stool samples, although conducted in private, might cause the individual mother/caretaker some inconvenience and embarrassment due to possible cultural barriers.

E.8. Confidentiality

No individual or household will be identified at any time in any reports or publication of the research. All data and material on the study subjects maintained for the study will be kept under lock-and-key and will be accessible only to project staff. Due to the nature of the intervention this is an open, unblinded trial, i.e. the intervention known to participant and project staff including the investigators.

E.9. Informed consent

Informed consent to participate in the trial relates to the diarrheal disease monitoring and the collection of stool specimen and household water samples only. The community will have consented to the introduction of the SODIS intervention prior to the trial trough their interaction with implementing agency.

If a household, i.e. the parents of the study child and all other household members is willing and eligible to participate in the randomized intervention trial the project staff (representative from Centro del Aguas y Saneamiento Ambiental, LABIMED, PCI and the Swiss Tropical Institute) will provide the consent form to the parents of the study child and all household members. The form will be read and explained to them (literate guardians must read the form). Adequate time will be given and the project representative will be available to answer questions. Both parents must read and sign the form in front of a witness of the community. Consent will also be obtained from all other household members. Repeated home visits will be done to obtain all signatures. Other household members will only participate in maintaining the household morbidity calendar. They will not be asked to fill out any documents or data forms, nor will they be interviewed or have stool samples collected from them. All consent forms are attached (Appendix M).

E.10. Financial Aspects

No payments, compensations or incentives for study participation will be paid.

F. Vertebrate Animals

No vertebrate animals are to be used in the proposed research

G. Literature Cited

Acosta, C. J., C. M. Galindo, D. Schellenberg, J. J. Aponte, E. Kahigwa, H. Urassa, J. R. Schellenberg, H. Masanja, R. Hayes, A. Y. Kitua, F. Lwilla, H. Mshinda, C. Menendez, M. Tanner, and P. L. Alonso. 1999. Evaluation of the SPf66 vaccine for malaria control when delivered through the EPI scheme in Tanzania. *Tropical Medicine and International Health* 4: 368-76.

Ahmed, F., J. D. Clemens, M. R. Rao, and A. K. Banik. 1994. Family latrines and paediatric shigellosis in rural Bangladesh: benefit or risk? *Int.J.Epidemiol.* 23: 856-862.

Ahmed, S. A., B. A. Hoque, and A. Mahmud. 1998. Water management practices in rural and urban homes: a case study from Bangladesh on ingestion of polluted water. *Public Health* 112: 317-321.

Alonso, P. L., M. C. Lopez, G. Bordmann, T. A. Smith, J. J. Aponte, N. A. Weiss, H. Urassa, J. R. Armstrong-Schellenberg, A. Y. Kitua, H. Masanja, M. C. Thomas, A. Oettli, N. Hurt, R. Hayes, W. L. Kilama, and M. Tanner. 1998. Immune responses to Plasmodium falciparum antigens during a malaria vaccine trial in Tanzanian children. *Parasite Immunology* 20: 63-71.

Alonso, P. L., M. Tanner, T. Smith, R. J. Hayes, J. A. Schellenberg, M. C. Lopez, I. Bastos de Azevedo, C. Menendez, E. Lyimo, N. Weiss, and et al. 1994. A trial of the synthetic malaria vaccine SPf66 in Tanzania: rationale and design. *Vaccine* 12: 181-6.

Anonymous. 1988. Persistent diarrhoea in children in developing countries: memorandum from a WHO meeting. *Bull World Health Organ* 66: 709-17.

Aziz, K. M., B. A. Hoque, K. Z. Hasan, M. Y. Patwary, S. R. Huttly, M. M. Rahaman, and R. G. Feachem. 1990. Reduction in diarrhoeal diseases in children in rural Bangladesh by environmental and behavioural modifications. *Trans.R.Soc.Trop.Med.Hyg.* 84: 433-438.

Bajolet, O., and C. Chippaux-Hyppolite. 1998. [Rotavirus and other viruses of diarrhea]. *Bull Soc Pathol Exot* 91: 432-7.

Ball, F. 1999. Stochastic and deterministic models for SIS epidemics among a population partitioned into households. *Math Biosci* 156: 41-67.

Basset, D., H. Gaumerais, and A. Basset-Pougnet. 1986. [Intestinal parasitoses in children of an Indian community of Bolivian altiplano]. *Bull Soc Pathol Exot Filiales* 79: 237-46.

Berke, T., B. Golding, X. Jiang, D. W. Cubitt, M. Wolfaardt, A. W. Smith, and D. O. Matson. 1997. Phylogenetic analysis of the Caliciviruses. *J Med Virol* 52: 419-24.

Bern, C., J. Martines, I. de Zoysa, and R. I. Glass. 1992. The magnitude of the global problem of diarrhoeal disease: a ten- year update. *Bull.World Health Organ.* 70: 705-714.

Binka, F. N., A. Kubaje, M. Adjuik, L. A. Williams, C. Lengeler, G. H. Maude, G. E. Armah, B. Kajihara, J. H. Adiamah, and P. G. Smith. 1996. Impact of permethrin impregnated bednets on child mortality in Kassena-Nankana district, Ghana: a randomized controlled trial [see comments]. *Trop.Med.Int.Health* 1: 147-154.

Blake, P. A., S. Ramos, K. L. MacDonald, V. Rassi, T. A. Gomes, C. Ivey, N. H. Bean, and L. R. Trabulsi. 1993. Pathogen-specific risk factors and protective factors for acute diarrheal disease in urban Brazilian infants. *J.Infect.Dis.* 167: 627-632.

Booth, M., H. L. Guyatt, Y. Li, and M. Tanner. 1996a. The morbidity attributable to Schistosoma japonicum infection in 3 villages in Dongting Lake region, Hunan province, PR China. *Tropical Medicine and International Health* 1: 646-54.

Booth, M., Y. Li, and M. Tanner. 1996b. Helminth infections, morbidity indicators and schistosomiasis treatment history in three villages, Dongting Lake region, PR China. *Tropical Medicine and International Health* 1: 464-74.

Brofferio, S. 2000. SODIS- Water Solar Disinfection Diffusion Project In Bolivia. Scuola Superiore San'anna- Regione Toscana- Caritas Diocesana di Pisa COSPE, Cochabamba.

Cairncross, S. 1989. Water supply and sanitation: an agenda for research. *J.Trop.Med.Hyg.* 92: 301-314.

Cairncross, S., and R. G. Feachem. 1993. *Environmental health engineering in the tropics.* John Wiley & Sons, New York.

Cancrini, G., A. Bartoloni, L. Nunez, and F. Paradisi. 1988. Intestinal parasites in the Camiri, Gutierrez and Boyuibe areas, Santa Cruz Department, Bolivia. *Parassitologia* 30: 263-9.

CdA, C. d. A. 1999. Programma de control y sequimiento de la calidad del agua en servicios de agua para comunidas manores a 5000 habitantes. Ministerio de viviende y servicios basico, Direction general de saneamiento basico, Cochabamba.

CdA, F. d. C. y. T.-U. E. S. 1997. Resumen Proyecto Demonstrativo SODIS en Sacabamba - Bolivia. Centro de Aguas y Saneamiento Ambiental, Facultad de Ciencias y Tecnologia, Universidad Mayor de San Simon, Cochabamba, Bolivia.

Centro de Desarrollo Agropecuario. 1994. PROVINCIA MIZQUE; PROGRAMA GLOBAL DE DESARROLLO. Centro de Desarrollo Agropecuario; Editado por CEDEAGRO, Cochabamba, Bolivia.

Cissé, G., P. Odermatt, and M. Tanner. 1999. *Recherche-action-formation (RAF): Etudes de cas à Ouagadougou (Burkina Faso) et à Nouakchott (Mauritania) sur la problématique de l'utilisation d'eaux polluées en agriculture urbaine.* Birkhäuser, Basel, 1999., Basel, 1999.

Conroy, R. M., M. Elmore-Meegan, T. Joyce, K. G. McGuigan, and J. Barnes. 1996. Solar disinfection of drinking water and diarrhoea in Maasai children: a controlled field trial. *Lancet* 348: 1695-1697.

Conroy, R. M., M. Elmore-Meegan, T. Joyce, K. G. McGuigan, and J. Barnes. in press. Solar disinfection of drinking water protects against cholera in children aged under 6. *NA* .

Conroy, R. M., M. E. Meegan, T. Joyce, K. McGuigan, and J. Barnes. 1999. Solar disinfection of water reduces diarrhoeal disease: an update. *Arch.Dis.Child* 81: 337-338.

Conroy, R. M., M. E. Meegan, T. Joyce, K. McGuigan, and J. Barnes. 2001. Solar disinfection of drinking water protects against cholera in children under 6 years of age. *Arch Dis Child* 85: 293-5.

Cunliffe, N. A., P. E. Kilgore, J. S. Bresee, A. D. Steele, N. Luo, C. A. Hart, and R. I. Glass. 1998. Epidemiology of rotavirus diarrhoea in Africa: a review to assess the need for rotavirus immunization. *Bull World Health Organ* 76: 525-37.

Donner, A., and N. Klar. 1994. Methods for comparing event rates in intervention studies when the unit of allocation is a cluster. *Am.J.Epidemiol.* 140: 279-89; discussion 300-1.

Eisenberg, J. N., E. Y. Seto, A. W. Olivieri, and R. C. Spear. 1996. Quantifying water pathogen risk in an epidemiological framework. *Risk.Anal.* 16: 549-563.

Eisenberg, J. N., W. K. Reisen, and R. C. Spear. 1995. Dynamic model comparing the bionomics of two isolated Culex tarsalis (Diptera: Culicidae) populations: sensitivity analysis. *J Med Entomol* 32: 98-106.

Eisenberg, J. N., E. Y. Seto, J. M. Colford, Jr., A. Olivieri, and R. C. Spear. 1998. An analysis of the Milwaukee cryptosporidiosis outbreak based on a dynamic model of the infection process [see comments]. *Epidemiology* 9: 255-63.

Eisenberg, J. N., E. Y. Seto, A. W. Olivieri, and R. C. Spear. 1996. Quantifying water pathogen risk in an epidemiological framework. *Risk.Anal.* 16: 549-563.

Esrey, S. A. 1996. Water, waste, and well-being: a multicountry study [see comments]. *Am.J.Epidemiol.* 143: 608-623.

Ford, T. E. 1999. Microbiological Safety of Drinking Water: United States and Global Perspectives. *Environ Health Perspect* 107 Suppl 1: 191-206.

Gagneux, S., C. Schneider, P. Odermatt, G. Cisse, D. Cheikh, M. L. Salem, A. Toure, and M. Tanner. 1999. [Diarrhea in urban agricultural workers in Nouakchott in Mauritania]. *Medecine Tropicale* 59: 253-8.

Goldman, N., B. Vaughan, and A. R. Pebley. 1998. The use of calendars to measure child illness in health interview surveys. *Int.J.Epidemiol.* 27: 505-512.

Grabow, W. O., and P. Coubrough. 1986. Practical direct plaque assay for coliphages in 100-ml samples of drinking water. *Appl Environ Microbiol* 52: 430-3.

Guerrant, R. L., J. M. Hughes, N. L. Lima, and J. Crane. 1990. Diarrhea in developed and developing countries: magnitude, special settings, and etiologies. *Rev Infect Dis* 12 Suppl 1: S41-50.

Habicht, J. P., C. G. Victora, and J. P. Vaughan. 1999. Evaluation designs for adequacy, plausibility and probability of public health programme performance and impact. *Int.J.Epidemiol.* 28: 10-18.

Hannan, P. J., D. M. Murray, D. R. Jacobs, Jr., and P. G. McGovern. 1994. Parameters to aid in the design and analysis of community trials: intraclass correlations from the Minnesota Heart Health Program. *Epidemiology.* 5: 88-95.

Havelaar, A. H., M. van Olphen, and Y. C. Drost. 1993. F-specific RNA bacteriophages are adequate model organisms for enteric viruses in fresh water. *Appl Environ Microbiol* 59: 2956-62.

Hayes, R. J., and S. Bennett. 1999. Simple sample size calculation for cluster-randomized trials. *Int.J.Epidemiol.* 28: 319-326.

Hobbins, M., D. Mäusezahl, and M. Tanner. 2000a. Home-based drinking water purification: The SODIS Health Study, Bangladesh. Swiss Tropical Institute, Basel, Berkeley, Rajshahi.

Hobbins, M., D. Mäusezahl, and M. Tanner. 2000b. Interim Report: SODIS Health Study. Swiss Tropical Institute, Basel, Berkeley, Rajshahi.

Hug, S. J., L. Canonica, M. Wegelin, D. Gechter, and U. Von Gunten. 2001. Solar oxidation and removal of arsenic at circumneutral pH in iron containing waters. *Environmental Science & Technology* 35: 2114-2121.

Huttly, S. R., S. S. Morris, and V. Pisani. 1997. Prevention of diarrhoea in young children in developing countries. *Bull.World Health Organ.* 75: 163-174.

INE. 2001. Censo 2001, Población por organizaciones comunitarias y localidades, www.ine.gov.bo.

Ise, T., Y. Tanabe, F. Sakuma, O. Jordan, E. Serrate, and H. Pena. 1994. Clinical evaluation and bacterial survey in infants and young children with diarrhoea in the Santa Cruz district, Bolivia. *J Trop Pediatr* 40: 369-74.

Joyce, T., K. G. McGuigan, M. Elmore-Meegan, and R. M. Conroy. 1996a. Prevalence of enteropathogens in stools of rural Maasai children under five years of age in the Maasailand region of the Kenyan Rift Valley. *East.Afr.Med.J.* 73: 59-62.

Joyce, T. M., K. G. McGuigan, M. Elmore-Meegan, and R. M. Conroy. 1996b. Inactivation of fecal bacteria in drinking water by solar heating. *Appl.Environ.Microbiol.* 62: 399-402.

Kalter, H. D., R. H. Gray, R. E. Black, and S. A. Gultiano. 1991. Validation of the diagnosis of childhood morbidity using maternal health interviews. *Int J Epidemiol* 20: 193-8.

Kirkwood, B. R., S. N. Cousens, C. G. Victora, and I. de Zoysa. 1997. Issues in the design and interpretation of studies to evaluate the impact of community-based interventions. *Trop Med Int Health* 2: 1022-9.

Klar, N., T. Gyorkos, and A. Donner. 1995. Cluster randomization trials in tropical medicine: a case study. *Trans.R.Soc.Trop.Med.Hyg.* 89: 454-459.

Last, J. 1995. *A Dictionnary in Epidemiology*. Oxford University Press, New York.

Lengeler, C., and R. W. Snow. 1996. From efficacy to effectiveness: insecticide-treated bednets in Africa. *Bull.World Health Organ.* 74: 325-332.

Levy, D. A., M. S. Bens, G. F. Craun, R. L. Calderon, and B. L. Herwaldt. 1998. Surveillance for waterborne-disease outbreaks--United States, 1995-1996. *Mor Mortal Wkly Rep CDC Surveill Summ* 47: 1-34.

Liang, K.-Y., and S. L. Zeger. 1986. Longitudinal data analysis using generalized linear models. *Biometrika* 73: 13-22.

Lloyd, B., and R. Helmer. 1991. *Surveillance of drinking water quality in rural areas*. Longman Scientific & Technical, New York.

Lopez, E., F. Barrera, J. Saavedra, E. Cona, and G. Aguilera. 1989. [Rotavirus in the feces of children with acute diarrhea in Cochabamba, Bolivia]. *Rev Chil Pediatr* 60: 34-5.

Manges, A. R., J. R. Johnson, B. Foxman, T. T. O'Bryan, K. E. Fullerton, and L. W. Riley. 2001. Widespread distribution of urinary tract infections caused by a multidrug-resistant Escherichia coli clonal group. *N Engl J Med* 345: 1007-13.

Marti, H., and J. C. Koella. 1993. Multiple stool examinations for ova and parasites and rate of false- negative results. *J Clin Microbiol* 31: 3044-5.

Mausezahl, D. 1996. Measuring the health impact of improved water supply and sanitation facilities in rural Zimbabwe. Pages 210. *Dept. of Public Health & Epidemiology*. University of Basel, Basel, Switzerland.

Mausezahl, D., F. Cheng, S. Q. Zhang, and M. Tanner. 1996. Hepatitis A in a Chinese urban population: the spectrum of social and behavioural risk factors. *Int J Epidemiol* 25: 1271-9.

McGuigan, K. G., T. M. Joyce, R. M. Conroy, J. B. Gillespie, and M. Elmore-Meegan. 1998. Solar disinfection of drinking water contained in transparent plastic bottles: characterizing the bacterial inactivation process. *J Appl Microbiol* 84: 1138-48.

Mintz, E., J. Bartram, P. Lochery, and M. Wegelin. 2001. Not Just a Drop in the Bucket: Expanding Access to Point-of-Use Water Treatment Systems. *Am J Public Health* 91: 1565-1570.

Mintz, E. D., F. M. Reiff, and R. V. Tauxe. 1995. Safe water treatment and storage in the home. A practical new strategy to prevent waterborne disease. *JAMA* 273: 948-953.

Morris, S. S., S. N. Cousens, C. F. Lanata, and B. R. Kirkwood. 1994. Diarrhoea--defining the episode. *Int.J.Epidemiol.* 23: 617-623.

Murray, C. J., and A. D. Lopez. 1994. Global and regional cause-of-death patterns in 1990. *Bull World Health Organ* 72: 447-80.

Murray, C. J., and A. D. Lopez. 1997. Global mortality, disability, and the contribution of risk factors: Global Burden of Disease Study [see comments]. *Lancet* 349: 1436-1442.

Murray, D. M. 1998. *Design and analysis of group-randomized trials*. Oxford University Press, New York, Oxford.

Myo Han, A., and K. Moe. 1990. Household faecal contamination and diarrhoea risk. *Journal of Tropical Medicine and Hygiene* 93: 333-6.

Nickson, R., J. McArthur, W. Burgess, K. M. Ahmed, P. Ravenscroft, and M. Rahman. 1998. Arsenic poisoning of Bangladesh groundwater [letter]. *Nature* 395: 338.

Payment, P. 1999. Poor efficacy of residual chlorine disinfectant in drinking water to inactivate waterborne pathogens in distribution systems. *Can.J.Microbiol.* 45: 709-715.

Payment, P., L. Richardson, J. Siemiatycki, R. Dewar, M. Edwardes, and E. Franco. 1991. A randomized trial to evaluate the risk of gastrointestinal disease due to consumption of drinking water meeting current microbiological standards. *Am.J.Public Health* 81: 703-708.

Pitts, M., J. McMaster, T. Hartmann, and D. Mausezahl. 1996. Lay beliefs about diarrhoeal diseases: their role in health education in a developing country. *Soc Sci Med* 43: 1223-8.

Prado, V., and M. L. O'Ryan. 1994. Acute gastroenteritis in Latin America. *Infect Dis Clin North Am* 8: 77-106.

Pruss, A. 1998. Review of epidemiological studies on health effects from exposure to recreational water. *Int J Epidemiol* 27: 1-9.

Quick, R. E., L. V. Venczel, O. Gonzalez, E. D. Mintz, A. K. Highsmith, A. Espada, E. Damiani, N. H. Bean, E. H. De Hannover, and R. V. Tauxe. 1996. Narrow-mouthed water storage vessels and in situ chlorination in a Bolivian community: a simple method to improve drinking water quality. *Am J Trop Med Hyg* 54: 511-6.

Quick, R. E., L. V. Venczel, E. D. Mintz, L. Soleto, J. Aparicio, M. Gironaz, L. Hutwagner, K. Greene, C. Bopp, K. Maloney, D. Chavez, M. Sobsey, and R. V. Tauxe. 1999. Diarrhoea prevention in Bolivia through point-of-use water treatment and safe storage: a promising new strategy. *Epidemiol.Infect.* 122: 83-90.

Reed, R. H. 1997. Solar inactivation of faecal bacteria in water: the critical role of oxygen. *Lett.Appl.Microbiol.* 24: 276-280.

Reed, R. H., S. K. Mani, and V. Meyer. 2000. Solar photo-oxidative disinfection of drinking water: preliminary field observations. *Lett Appl Microbiol* 30: 432-6.

Reiff, F. M., M. Roses, L. Venczel, R. Quick, and V. M. Witt. 1996. Low-cost safe water for the world: a practical interim solution. *J Public Health Policy* 17: 389-408.

Reller, M. E., Y. J. M. Mong, R. M. Hoekstra, and R. E. Quick. 2001. Cholera Prevention With Traditional and Novel Water Treatment Methods: An Outbreak Investigation in Fort-Dauphin, Madagascar. *Am J Public Health* 91: 1608-1610.

Ries, A. A., D. J. Vugia, L. Beingolea, A. M. Palacios, E. Vasquez, J. G. Wells, N. Garcia Baca, D. L. Swerdlow, M. Pollack, N. H. Bean, and et al. 1992. Cholera in Piura, Peru: a modern urban epidemic. *J Infect Dis* 166: 1429-33.

Roberts, L., Y. Chartier, O. Chartier, G. Malenga, M. Toole, and H. Rodka. 2001. Keeping clean water clean in a Malawi refugee camp: a randomized intervention trial. *Bull World Health Organ* 79: 280-7.

Romero, A. M., M. T. Iniguez, G. Gonzales, and T. Mariza Aanez. 1992. Sanamiento vasico en zonas perifericas de la ciudas de Cochabamba. Universidad Major de San Simone, Cochabamba.

Ruuskanen, O., O. Meurman, and G. Akusjarvi. 1997. Adenovirus. Pages 525-547 in D. D. Richmann, R. J. Whitley, and F. G. Hayden, eds. *Clinical Virology*. Churchill Livingston, US.

Samanta, B., and C. Van Wijk. 1998. Criteria for successful sanitation programmes in low income countries. *Health Policy & Planning* 13: 78-86.

Sarmiento, J. 1998. El Niño Southern Oscillation - ENSO 1997/98 And Risk Management In The Latin American And The Caribbean Region. USAID/OFDA.

Scrimshaw, S. C., and E. Hurtado. 1988. Anthropological involvement in the Central American diarrheal disease control project. *Soc Sci Med* 27: 97-105.

Seigel, R., C. Sant'anna, K. Salgado, and P. de Jesus. 1996. Acute diarrhoea among children from high and low socioeconomic communities in Salvador Brasil. *Int.J.Infect.Dis.* 1: 28-34.

Shier, R. P., N. Dollimore, D. A. Ross, F. N. Binka, M. Quigley, and P. G. Smith. 1996. Drinking water sources, mortality and diarrhoea morbidity among young children in northern Ghana. *Trop.Med.Int.Health* 1: 334-341.

Simpson, J. M., N. Klar, and A. Donnor. 1995. Accounting for cluster randomization: a review of primary prevention trials, 1990 through 1993. *Am.J.Public Health* 85: 1378-1383.

Smith, K. R., J. M. Samet, I. Romieu, and N. Bruce. 2000. Indoor air pollution in developing countries and acute lower respiratory infections in children. *Thorax* 55: 518-32.

Smith, P. G., and R. H. Morrow. 1991. *Methods for field trials of interventions against tropical diseases : a "toolbox"*. Oxford University Press, Oxford ; New York.

Sommer, B., M. A., and S. Y.L. 1997. SODIS - an emerging water treatment process. *J.Water SRT-Aqua* 46: 127-137.

Stanton, B. F., and J. D. Clemens. 1987. An educational intervention for altering water-sanitation behaviors to reduce childhood diarrhea in urban Bangladesh. II. A randomized trial to assess the impact of the intervention on hygienic behaviors and rates of diarrhea. *Am.J.Epidemiol.* 125: 292-301.

Steele, A. D., H. R. Basetse, N. R. Blacklow, and J. E. Herrmann. 1998. Astrovirus infection in South Africa: a pilot study. *Ann Trop Paediatr* 18: 315-9.

Swerdlow, D. L., E. D. Mintz, M. Rodriguez, E. Tejada, C. Ocampo, L. Espejo, K. D. Greene, W. Saldana, L. Seminario, R. V. Tauxe, and et al. 1992. Waterborne transmission of epidemic cholera in Trujillo, Peru: lessons for a continent at risk [see comments]. *Lancet* 340: 28-33.

Tanner, M., C. Lengeler, and N. Lorenz. 1993. Case studies from the biomedical and health systems research activities of the Swiss Tropical Institute in Africa. *Trans.R.Soc.Trop.Med.Hyg.* 87: 518-523.

The World Bank. 1993. *The world health report; Investing in health. World development indicators.* Oxford University Press, New York.

The World Bank. 1998. UNDP-World Bank Water and Sanitation Program Report 1997-98. Pages 44. UNDP-World Bank, Washington.

Toranzos, G. 1986. Présence de virus enterique dans les eaux de consommation à Cochabamba (Bolivie). *Revue international des Science de l'eau* 2: 91-93.

Tornieporth, N. G., J. John, K. Salgado, P. de Jesus, E. Latham, M. C. Melo, S. T. Gunzburg, and L. W. Riley. 1995. Differentiation of pathogenic Escherichia coli strains in Brazilian children by PCR. *J Clin Microbiol* 33: 1371-4.

Townes, J. M., R. Quick, O. Y. Gonzales, M. Linares, E. Damiani, C. A. Bopp, S. P. Wahlquist, L. C. Hutwagner, E. Hanover, E. D. Mintz, and R. V. Tauxe. 1997. Etiology of bloody diarrhea in Bolivian children: implications for empiric therapy. Bolivian Dysentery Study Group. *J Infect Dis* 175: 1527-30.

Unicomb, L. E., N. N. Banu, T. Azim, A. Islam, P. K. Bardhan, A. S. Faruque, A. Hall, C. L. Moe, J. S. Noel, S. S. Monroe, M. J. Albert, and R. I. Glass. 1998. Astrovirus infection in association with acute, persistent and nosocomial diarrhea in Bangladesh. *Pediatr Infect Dis J* 17: 611-4.

Utsunomiya, A., D. Elio-Calvo, A. A. Reyes, E. S. Castro, E. Rodriguez, C. Tress, J. I. de Corzo, E. Hannover, A. Kai, K. Tamura, and et al. 1995. Major enteropathogenic bacteria isolated from diarrheal patients in Bolivia: A hospital-based study. *Microbiol Immunol* 39: 845-51.

Utzinger, J., E. K. N'Goran, H. P. Marti, M. Tanner, and C. Lengeler. 1999. Intestinal amoebiasis, giardiasis and geohelminthiases: their association with other intestinal parasites and reported intestinal symptoms. *Trans.R.Soc.Trop.Med.Hyg.* 93: 137-141.

Utzinger, J., E. K. N'Goran, A. N'Dri, C. Lengeler, X. Shuhua, and M. Tanner. 2000a. Oral artemether for prevention of Schistosoma mansoni infection: randomised controlled trial. *Lancet* 355: 1320-5.

Utzinger, J., E. K. N'Goran, Y. A. Ossey, M. Booth, M. Traoré, K. L. Lohourignon, A. Allangba, L. A. Ahiba, M. Tanner, and C. Lengeler. 2000b. Rapid screening for Schistosoma mansoni in western Côte d'Ivoire using a simple school questionnaire. *Bulletin of the World Health Organization* 78: 389-98.

Vanderslice, J., and J. Briscoe. 1993. All Coliforms Are Not Created Equal - a Comparison of the Effects of Water Source and in-House Water Contamination On Infantile Diarrheal Disease. *Water Resources Research* 29: 1983-1995.

Weber, J. T., E. D. Mintz, R. Canizares, A. Semiglia, I. Gomez, R. Sempertegui, A. Davila, K. D. Greene, N. D. Puhr, D. N. Cameron, and et al. 1994. Epidemic cholera in Ecuador: multidrug-resistance and transmission by water and seafood. *Epidemiol Infect* 112: 1-11.

Wegelin, M. 1998. SODIS News. Pages 1-18. EAWAG/SANDEC, Swiss Federal Institute for Environmental Science & Technology/ Water Sanitation in Developing Countries, Duebendorf; Switzerland.

Wegelin, M., S. Canonica, A. C. Alder, D. Marazuela, M. J. F. Suter, T. D. Bucheli, O. P. Haefliger, R. Zenobi, K. G. McGuigan, M. T. Kelly, P. Ibrahim, and M. Larroque. 2001. Does sunlight change the material and content of polyethylene terephthalate (PET) bottles? *Journal of Water Supply Research and Technology-Aqua* 50: 125-133.

Wegelin, M., S. Canonica, K. Mechsner, T. Fleischmann, F. Pesario, and A. Metzler. 1994. Solar water disinfection (SODIS): Scope of the process and analysis of radiation experiments. *J.Water SRT-Aqua* 43: 154-169.

Wegelin, M., and B. Sommer. 1998. Solar water disinfection (SODIS) - destined for worldwide use? *Waterlines* 16: 30-32.

Weiss, M. G. 1988. Cultural models of diarrheal illness: conceptual framework and review. *Soc Sci Med* 27: 5-16.

Wolfaardt, M., M. B. Taylor, H. F. Booysen, L. Engelbrecht, W. O. Grabow, and X. Jiang. 1997. Incidence of human calicivirus and rotavirus infection in patients with gastroenteritis in South Africa. *J Med Virol* 51: 290-6.

World Health Organisation. 1985. *Guidelines for Drinking -Water Quality*. World Health Organisation, Geneva, Switzerland.

World Health Organisation. 1992. The International Drinking Water Supply and Sanitation Decade: End of Decade Review. World Health Organisation, Geneva, Switzerland.

Yang, J., and T. Scholten. 1977. A fixative for intestinal parasites permitting the use of concentration and permanent staining procedures. *Am J Clin Pathol* 67: 300-4.

Zeger, S. L., K. Y. Liang, and P. S. Albert. 1988. Models for longitudinal data: a generalized estimating equation approach [published erratum appears in Biometrics 1989 Mar;45(1):347]. *Biometrics* 44: 1049-60.

Zerbini, C. 2000. The inactivation of *Cryptosporidium spp* oocysts, and *Giardia lamblia* cysts by solar disinfection". Pages 49. *Dept Public Health & Epidemiology, Swiss Tropical Institute, Basel*. Swiss Tropical Institute, University of Basel, Basel / Switzerland.
