# Supplementary material for: Solar Drinking Water Disinfection (SODIS) to Reduce Childhood Diarrhoea in Rural Bolivia: A Cluster-Randomized, Controlled Trial
Source: PLoS Med. 2009 Aug 18;6(8):e1000125. doi: 10.1371/journal.pmed.1000125 (PMC2719054; doi:10.1371/journal.pmed.1000125)
Supplement: Text S2 — CONSORT statement checklist. (0.10 MB PDF) [file pmed.1000125.s004.pdf]

CONSORT Statement

Checklist of items to include when reporting a cluster randomised trial
(adaptations from standard guidelines in italic)

PAPER SECTION **And topic**	**Item**	**Descriptor**	**Reported in section**
TITLE & ABSTRACT	1	How participants were allocated to interventions (eg random allocation, randomised, or randomly assigned), *specifying that allocation was based on clusters.*	Title
INTRODUCTION Background	2	Scientific background and explanation of rationale, *including the rationale for using a cluster design*	Introduction
METHODS Participants	3	Eligibility criteria for participants *and clusters* and the settings and locations where the data were collected	Methods: Site and population, Design; Fig. 1
Interventions	4	Precise details of the interventions intended for each group, *whether they pertain to the individual level, the cluster level, or both,* and how and when they were actually administered.	Methods: Implementation of the intervention; Fig. S1
Objectives	5	Specific objectives and hypotheses *and whether they pertain to the individual level, the cluster level, or both.*	Introduction
Outcomes	6	Report clearly defined primary and secondary outcome measures, *whether they pertain to the individual level, the cluster level, or both*, and, when applicable, any methods used to enhance the quality of measurements (eg multiple observations, training of assessors)	Methods: Outcome, Data collection and field staff
Sample size	7	How *total* sample size was determined *(including method of calculation, number of clusters, cluster size, a coefficient of intracluster correlation (ICC or k), and an indication of its uncertainty*) and, when applicable, explanation of any interim analyses and stopping rules.	Methods: Design
Randomization -- Sequence generation	8	Method used to generate the random allocation sequence, including details of any restriction (eg blocking, stratification, *matching*) Allocation.	Methods: Design
Randomization -- Allocation concealment	9	Method used to implement the random allocation sequence, *specifying that allocation was based on clusters rather than individuals and* clarifying whether the sequence was concealed until interventions were assigned.	Methods: Design
Randomization -- Implementation	10	Who generated the allocation sequence, who enrolled participants, and who assigned participants to their groups.	Methods: Design
Blinding (masking)	11	Whether participants, those administering the interventions, and those assessing the outcomes were blinded to group assignment. If done, how the success of blinding was evaluated.	Methods: Design; Discussion
Statistical methods	12	Statistical methods used to compare groups for primary outcome(s) *indicating how clustering was taken into account*; methods for additional analyses, such as subgroup analyses and adjusted analyses.	Methods: Statistical analysis
RESULTS Participant flow	13	Flow of *clusters and* individual participants through each stage. Specifically, for each group report the numbers of *clusters and* participants randomly assigned, receiving intended treatment, completing the study protocol, and analysed for the primary outcome. Describe protocol deviations from study as planned, together with reasons.	Results: Participant flow and recruitment; Fig. 1
Recruitment	14	Dates defining the periods of recruitment and follow up.	Results: Participant flow and recruitment; Figs. 1, S1
Baseline data	15	Baseline information for each group *for the individual and cluster levels as applicable.*	Methods: Baseline characteristics; Table 2
Numbers analyzed	16	Number of *clusters and* participants (denominator) in each group included in each analysis and whether the analysis was by intention to treat.	Methods: Statistical analysis; Fig. 1; Table 3
Outcomes and estimation	17	For each primary and secondary outcome, a summary of results for each group *for the individual or cluster level as applicable*, and the estimated effect size and its precision (eg 95% confidence interval) *and a coefficient of intracluster correlation (ICC or k) for each primary outcome.*	Results: Diarrhoeal illness in the control and intervention arm; Table 3
Ancillary analyses	18	Address multiplicity by reporting any other analyses performed, including subgroup analyses and adjusted analyses, indicating those prespecified and those exploratory.	Methods: Statistical analysis; Results: Diarrhoeal illness by compliance; Table 3
Adverse events	19	All important adverse events or side effects in each intervention group.	na
DISCUSSION Interpretation	20	Interpretation of the results, taking into account study hypotheses, sources of potential bias or imprecision and the dangers associated with multiplicity of analyses and outcomes.	Discussion
Generalizability	21	Generalisability (external validity) *to individuals and/or clusters (as relevant)* of the trial findings.	Discussion
Overall evidence	22	General interpretation of the results in the context of current evidence.	Discussion
